# An intramolecular photoswitch can significantly promote photoactivation of Pt(iv) prodrugs[Fn fn1]

**DOI:** 10.1039/d0sc06839j

**Published:** 2021-04-01

**Authors:** Zhiqin Deng, Cai Li, Shu Chen, Qiyuan Zhou, Zoufeng Xu, Zhigang Wang, Houzong Yao, Hajime Hirao, Guangyu Zhu

**Affiliations:** Department of Chemistry, City University of Hong Kong Hong Kong SAR P. R. China guangzhu@cityu.edu.hk; City University of Hong Kong Shenzhen Research Institute Shenzhen 518057 P. R. China; School of Pharmaceutical Sciences, Health Science Center, Shenzhen University Shenzhen P. R. China

## Abstract

Selective activation of prodrugs at diseased tissue through bioorthogonal catalysis represents an attractive strategy for precision cancer treatment. Achieving efficient prodrug photoactivation in cancer cells, however, remains challenging. Herein, we report two Pt(iv) complexes, designated as rhodaplatins {rhodaplatin **1**, [Pt(CBDCA-*O*,*O*)(NH_3_)_2_(RhB)OH]; rhodaplatin **2**, [Pt(DACH)ox(RhB)(OH)], where CBDCA is cyclobutane-1,1-dicarboxylate, RhB is rhodamine B, DACH is (1*R*,2*R*)-1,2-diaminocyclohexane, and ox is oxalate}, that bear an internal photoswitch to realize efficient accumulation, significant co-localization, and subsequent effective photoactivation in cancer cells. Compared with the conventional platform of external photocatalyst plus substrate, rhodaplatins presented up to 4.8 10^4^-fold increased photoconversion efficiency in converting inert Pt(iv) prodrugs to active Pt(ii) species under physiological conditions, due to the increased proximity and covalent bond between the photoswitch and Pt(iv) substrate. As a result, rhodaplatins displayed increased photocytotoxicity compared with a mixture of RhB and conventional Pt(iv) compound in cancer cells including Pt-resistant ones. Intriguingly, rhodaplatin **2** efficiently accumulated in the mitochondria and induced apoptosis without causing genomic DNA damage to overcome drug resistance. This work presents a new approach to develop highly effective prodrugs containing intramolecular photoswitches for potential medical applications.

## Abstract 2

The newly developed Pt(iv) prodrugs, rhodaplatins, contain an internal photoswitch and present up to 4.8 10^4^-fold increased photoconversion efficiency compared to the conventional photocatalyst plus Pt(iv) prodrug photocatalysis platform.

## Graphical abstract

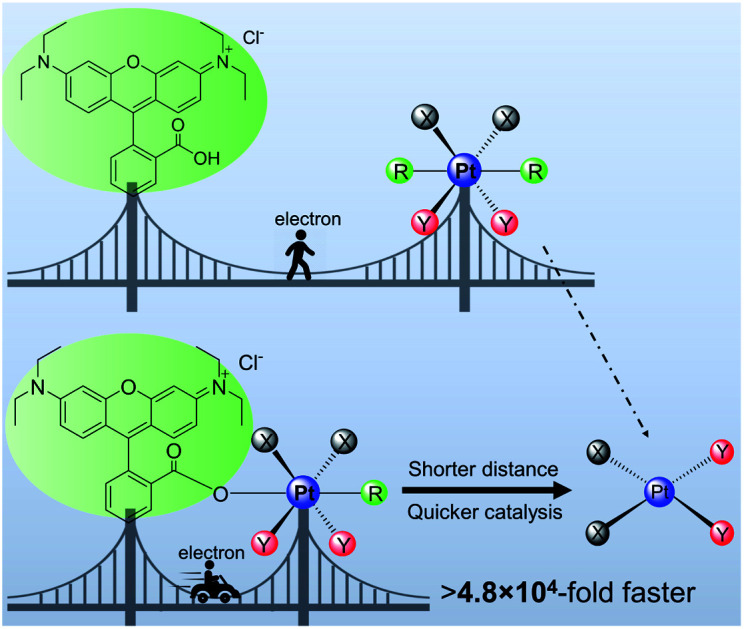

## Introduction

Conventional chemotherapeutic drugs have achieved great success in fighting cancer.^1^ Their therapeutic outcome, however, is limited by severe adverse effects induced by undesired activation of the drugs at nonpathological sites.^2^ Various strategies have been developed to address this limitation, including developing kinetically inert prodrugs that can be preferably activated only in cancer cells,^3^ adding external catalysts to selectively activate drugs at the desired site,^4^ or *in situ* drug synthesis.^5^ The improved biological potential has been observed for these strategies. The activation of these anticancer agents is, however, passively reliant on the intracellular environment of cancer cells; premature or incomplete activation is likely to occur and compromise their anticancer activities. Therefore, drugs that can be specifically and effectively activated at the pathological site are still highly desired for precision medicine.

The emerging concept of combining photocatalysis and bioorthogonal reactions for biological and medicinal applications has drawn much attention.^6^ A common strategy is to use external photocatalysts to activate Pt(iv) anticancer prodrugs in cancer cells to improve the prodrugs' cancer selectivity. For instance, riboflavin has been found to catalytically reduce Pt(iv) prodrugs to active Pt(ii) drugs upon visible light irradiation.^7^ Recently, a ruthenium-based photosensitizer has also been utilized as the photocatalyst to activate Pt(iv) prodrugs.^8^ However, the photocatalysts do not always effectively co-localize with the Pt(iv) substrate in the cancer cells, limiting their photocatalytic efficiency. Another concern is the intracellular stability of the Pt(iv) prodrugs, some of which may be photoactivated outside of cells. Moreover, other intracellular biomolecules may competitively react with the Pt(iv) substrate or catalyst.^9^ Therefore, the current platform of external photocatalyst plus Pt(iv) substrate has its own limitations regarding biological applications. Indeed, the co-treatment strategy has achieved only limited enhancement in cytotoxicity compared with the original Pt(ii) drugs.

To address these limitations, we designed a new class of photoactivatable Pt(iv) prodrugs based on clinical Pt(ii) drugs. The highly stable prodrugs contain an internal photoswitch to realize effective photoactivation in cancer cells. The internalized photoswitch that is colocalized with the Pt center ensures the prodrugs dramatically boosted intracellular activation efficiency and significantly increased photocytotoxicity compared with the external catalyst plus substrate platform. Interestingly, one of the prodrugs precisely located and damaged the mitochondria, an unconventional target of Pt-based complexes. Compared with nuclei, mitochondria lack the function of nucleotide excision repair (NER) and histone protection,^10^ the two main factors responsible for the resistance of cancer cells towards Pt drugs. In addition, inducing mitochondrial DNA damage could initiate mitochondria-mediated cell death pathways.^11^ Therefore, by targeting mitochondria, rhodaplatins may effectively kill cancer cells and overcome Pt resistance. We provide a novel strategy to develop highly effective photoactivatable Pt(iv) prodrugs for controllable and selective activation in cancer cells.

## Results and discussion

**Fig. 1:**
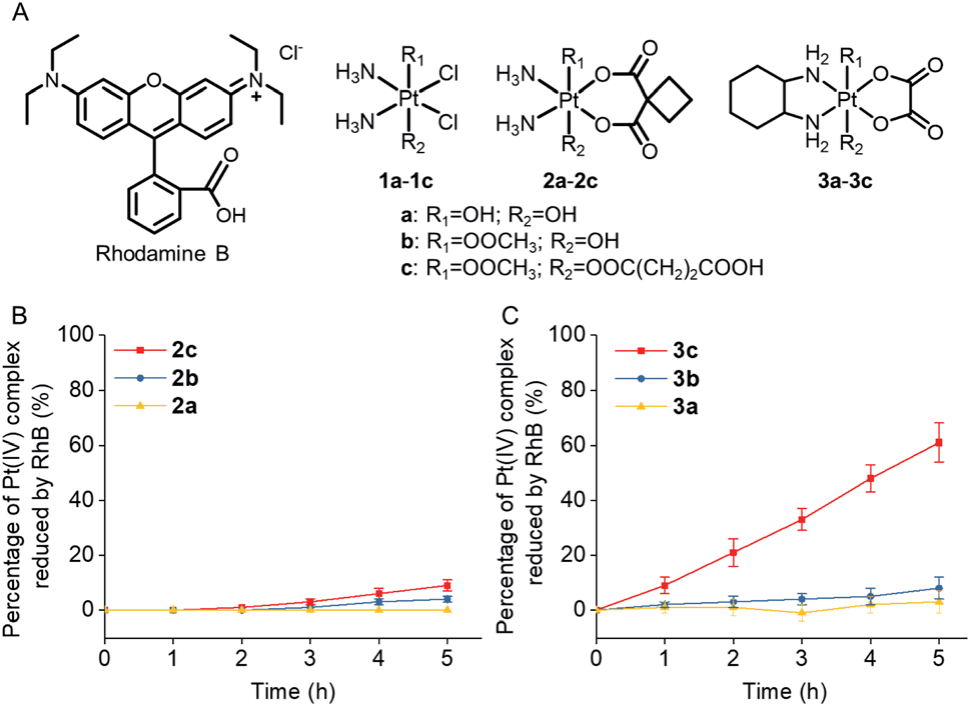
(A) The chemical structures of rhodamine B (RhB) and complexes **1a3c**. The percentage of (B) complexes **2a2c** (10^4^ M) and (C) complexes **3a3c** (10^4^ M) that are catalytically reduced by RhB (10^4^ M) in the presence of sodium ascorbate (2 10^3^ M) upon irradiation (400760 nm, 4 mW cm^2^).

Rhodamine B (RhB, Fig. 1A), a widely used fluorescent dye,^12^ has been employed as a photocatalyst for various reactions.^13^ We noticed that the oxidation potential of the photo-excited RhB (RhB^+^/RhB*: 1.3 V)^13*a*^ is theoretically sufficient to reduce most of the conventional Pt(iv) prodrugs.^14^ Therefore, we first investigated the ability of RhB to catalytically reduce Pt(iv) prodrugs in the presence of a reducing agent upon visible light irradiation. Cisplatin-, carboplatin-, and oxaliplatin-based Pt(iv) prodrugs containing various axial ligands were obtained (complexes **1a3c**; Fig. 1A, S1S6 and Scheme S1[Fn fn1]). The oxaliplatin- and carboplatin-based but not the cisplatin-based Pt(iv) prodrugs were stable in the presence of reducing agents (Fig. S7[Fn fn1]). Thus, the cisplatin-based Pt(iv) prodrugs were excluded from the following studies. For the carboplatin-based Pt(iv) complexes, complexes **2a2c** (10^4^ M) were mixed with RhB (10^4^ M) in PBS buffer (pH 7.4) containing 2 10^3^ M sodium ascorbate. No reduction of the complexes was observed in the absence of light. Upon irradiation with white light (400760 nm, 4 mW cm^2^), although the reduction of **2a** was barely observed, 4% of **2b** and 9% of **2c** were reduced to carboplatin after irradiation for 5 h, and no significant change of RhB was observed during irradiation (Fig. S8S10[Fn fn1]), indicating that RhB could catalytically reduce complexes **2b** and **2c** to carboplatin but with poor catalytic efficiency (Fig. 1B). A similar scenario was observed for the oxaliplatin-based Pt(iv) substrates (Fig. 1C and S11S13[Fn fn1]). Our further study indicated that the catalytic efficiency correlated with the reduction potential of Pt(iv) substrate (Fig. S14, S15 and Table S1[Fn fn1]), and the low electron transfer efficiency between RhB and the Pt(iv) center was the bottleneck that limited the catalytic efficiency of such photocatalysis platform (Fig. S16S18[Fn fn1]).

**Fig. 2:**
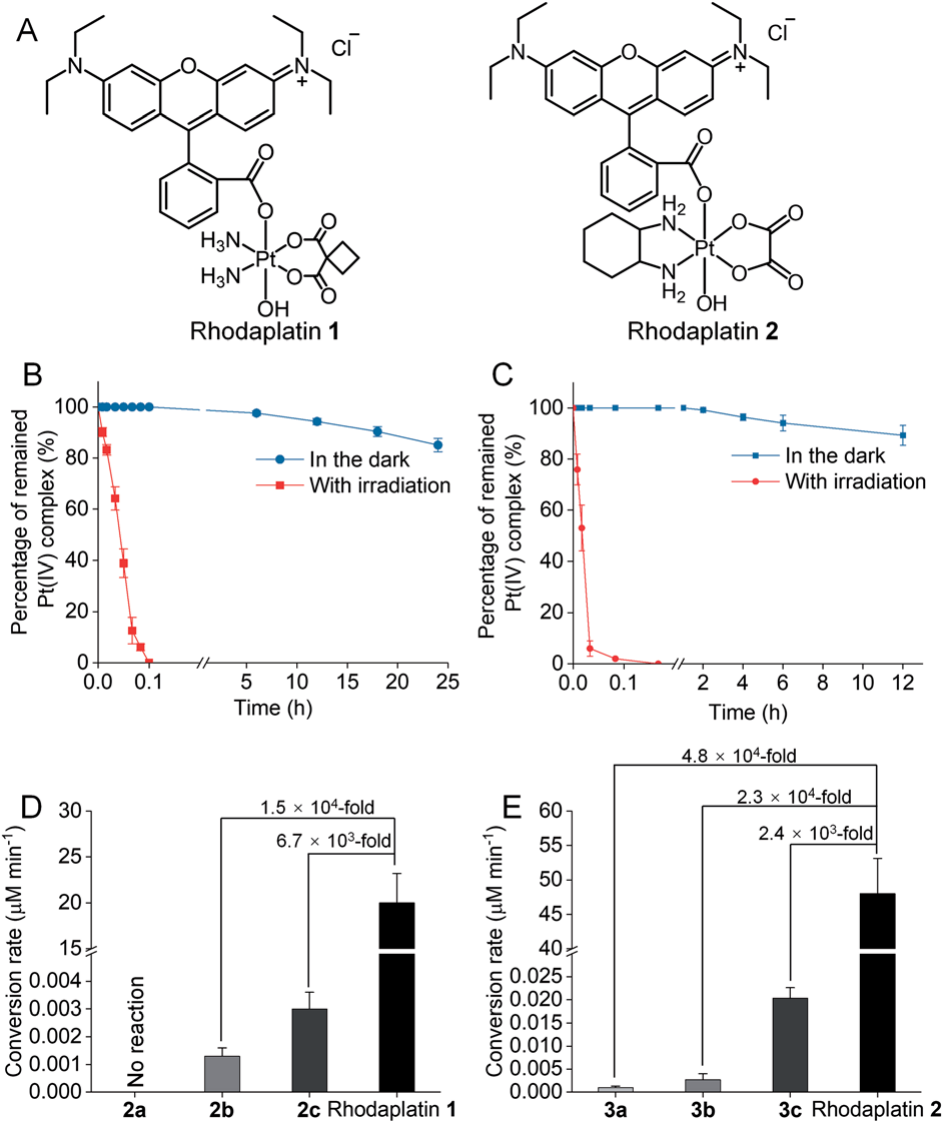
(A) The chemical structures of rhodaplatin **1** and rhodaplatin **2**. The percentage of remained (B) rhodaplatin **1** (10^4^ M) and (C) rhodaplatin **2** (10^4^ M) in the PBS buffer (pH 7.4) containing 2 10^3^ M sodium ascorbate with or without irradiation with white light (400760 nm, 4 mW cm^2^). (D) The calculated conversion rate of rhodaplatin **1** and RhB towards **2a2c** at the concentration of 10^4^ M. (E) The calculated conversion rate of rhodaplatin **2** and RhB towards **3a3c** at the concentration of 10^4^ M.

To improve the photoconversion performance, we speculated that shortening the distance between the photocatalyst and the substrate to increase the electron transfer efficiency might be a promising approach. To verify this hypothesis, we directly conjugated RhB with carboplatin- and oxaliplatin-based Pt(iv) complexes, such that the distance between the photoswitch and the Pt(iv) center significantly decreased. The synthetic complexes were designated as rhodaplatin **1** and rhodaplatin **2**, for the carboplatin- and oxaliplatin-based prodrugs, respectively (Fig. 2A, S19, S20 and Scheme S2[Fn fn1]). As rhodaplatins were designed to be activated in cancer cells, which have abundant reducing agents (*e.g.*, sodium ascorbate, GSH, or NADPH),^15^ we monitored the stability and photoreduction of rhodaplatins in the presence of such reducing agents. Rhodaplatins showed high dark stability even in the presence of sodium ascorbate; more than 94% of rhodaplatin **1** and 88% of rhodaplatin **2** remained after incubation for 12 h (Fig. S21[Fn fn1]). Upon irradiation with low-dose visible light (400760 nm, 4 mW cm^2^), however, 95% of rhodaplatins were converted to the corresponding Pt(ii) drugs within 5 min in PBS buffer (pH 7.4) containing sodium ascorbate (Fig. 2B, C, S22 and S23[Fn fn1]). High stability and rapid photoreduction were also observed in the presence of glutathione (Fig. S24 and S25[Fn fn1]). In the presence of ascorbate, the conversion rate of rhodaplatin **1** at 10^4^ M was calculated to be 2 10^5^ M min^1^, which is 1.5 10^4^-fold and 6.7 10^3^-fold higher than that of RhB towards complex **2b** and **2c**, respectively (Fig. 2D). Similarly, the conversion rate of rhodaplatin **2** at 10^4^ M was up to 4.8 10^4^-fold higher than that of RhB towards oxaliplatin-based Pt(iv) substrates (Fig. 2E). Notably, compared with riboflavin, an effective photocatalyst to convert Pt(iv) prodrugs to Pt(ii) forms,^7*a*^ the photocatalysis efficiency of free RhB was 6.6 10^4^ to 1.32 10^6^ times lower than that of riboflavin, but rhodaplatins presented a comparable photoconversion rate with that from riboflavin. These data confirm that the enhanced proximity and the covalent bond between the photoswitch and the Pt(iv) center could significantly accelerate the photoconversion process.

To further investigate how the distance and the covalent bond between the photoswitch and the Pt center would affect the conversion efficiency, the photo-reduction of rhodaplatins was investigated in the presence of excess Pt(iv) substrate or RhB at various concentrations. As shown in Fig. S26 and S27,[Fn fn1] no increase in the photoconversion efficiency of rhodaplatins was observed, even when high concentrations of Pt(iv) complex or RhB were added, indicating that a distantly separated Pt(iv) substrate and RhB can hardly affect the internal photo-reduction of rhodaplatins, further emphasizing the importance of the distance and covalent bond between the photoswitch and the Pt(iv) center. Next, to study the rate law of rhodaplatin, the impacts of irradiation power density and concentration of rhodaplatin and sodium ascorbate on the photo-reduction of rhodaplatin were analyzed. The photoreduction rate of rhodaplatin increased linearly with the power density of irradiation and the concentration of rhodaplatin, indicating a first-order reaction (Fig. S28 and S29[Fn fn1]).

As rhodaplatins are presented as monovalent cations in aqueous solutions, the prodrugs may be able to form ion-pairs with ascorbate. To investigate such a possibility, we first determined the ion-pair formation between rhodaplatin **2** and ascorbate in Milli-Q water. The prodrug formed ion-pair with ascorbate in a 1:1 stoichiometry (Fig. S30[Fn fn1]). The association constant (*K*_a_) value of such ion-pair in Milli-Q water was determined to be 3149 M^1^ by UV-Vis spectroscopic titration (Fig. S31[Fn fn1]).^16^ A similar result was obtained by fluorescence spectroscopic titration (Fig. S32[Fn fn1]). In PBS buffer, however, the anions including phosphates showed a much higher affinity towards rhodaplatin cations (Fig. S33[Fn fn1]); only very limited rhodaplatin **2** could form ion-pair with ascorbate, determined by NMR titration (Fig. S34[Fn fn1]), indicating that rhodaplatin may need to obtain electrons directly from free ascorbate in PBS buffer. At low concentrations of ascorbate, the conversion rate of rhodaplatin in PBS buffer increased with the ascorbate concentration, but the rate became nearly constant at high concentrations of ascorbate. The leveling off effect is dependent on irradiation power intensity (Fig. S35[Fn fn1]), indicating that when there is sufficient ascorbate, the number of photoexcited rhodaplatin is the limiting factor for the photoconversion rate in PBS buffer.

**Scheme 1:**
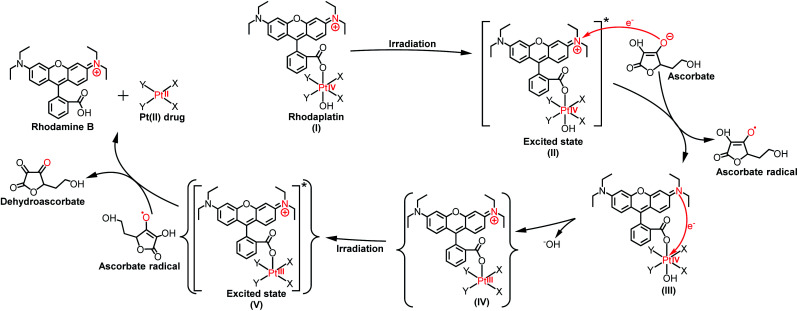
The proposed photoreduction mechanism of rhodaplatins in the presence of ascorbate under physiological conditions. Curly brackets are used to denote transient and undetected intermediates.

As the reduction potential of a free rhodamine ligand significantly decreases after photoexcitation,^17^ electron transfer from the excited rhodamine ligand to the Pt center may occur to reduce the Pt(iv) complex. To verify this hypothesis, we determined the fluorescence quantum yield and lifetime of RhB and rhodaplatins in aqueous solutions. As shown in Table S2,[Fn fn1] free RhB presented a higher quantum yield (0.34 *vs.* 0.18 and 0.19) and a longer fluorescence lifetime (2.0 *vs.* 1.0 and 1.1 ns) than rhodaplatins, indicating electron transfer from the excited rhodamine moiety to the Pt center.^18^ After photoactivation, the absorption and fluorescence intensity of the completely photoactivated products were very close to those from the same amount of free RhB (Fig. S36 and S37[Fn fn1]); no fragment of RhB was detected in the photoreduction products (Fig. S22, S23 and S25[Fn fn1]), indicating the RhB ligand remained intact during the photoactivation process. Therefore, electron transfer from reducing agents to the Pt center through the excited RhB ligand may occur. For this reason, we measured the interaction between free RhB and sodium ascorbate. In the presence of sodium ascorbate, the absorption of RhB rapidly changed (Fig. S38[Fn fn1]), indicating the formation of the reduced counterpart.^19^ The formation of ascorbate radicals in the mixture of rhodaplatin **2** and sodium ascorbate upon irradiation was confirmed by electron paramagnetic resonance (EPR) spectroscopy (Fig. S39[Fn fn1]), ascertaining that ascorbate is the electron donor for the photoreduction of rhodaplatins. Based on these observations, we propose a possible photoreduction mechanism of rhodaplatins in the presence of reducing agents (*e.g.*, sodium ascorbate). As shown in Scheme 1, rhodaplatins were designed to be activated in physiological environments, in which rhodamine and its derivatives are present in the cationic form (I).^20^ Upon visible light irradiation, the rhodaplatin is first transformed to its excited state (II), then an electron from ascorbate is transferred to the RhB ligand to form the Pt(iv) intermediate containing the RhB radical (III).^13*a*^ Since RhB and the Pt(iv) center are connected by a covalent bond, which can facilitate the transfer of the extra electron to the Pt(iv) center to yield the Pt(iii) intermediate (IV) along with the release of a hydroxyl group. After that, the Pt(iii) intermediate is promoted to its excited state by irradiation, and steps (II) to (IV) are repeated to generate the Pt(ii) drug and free RhB ligand.

As rhodaplatins presented significantly greater reduction efficiency than the RhB plus Pt(iv) platform, we subsequently analyzed how this boosted photoconversion efficiency would affect their biological activities. We first measured the cellular accumulation of rhodaplatins in A2780cisR platinum-resistant ovarian cancer cells. Most rhodaplatins accumulated in the cells within 6 h (Fig. S40[Fn fn1]); thus 6 h was chosen as the treatment time for the following cell-based assays. More importantly, after incubation for 6 h, more than 90% of rhodaplatins have remained stable in the culture medium or cancer cells; whereas after irradiation for 10 min with white light, more than 93% of rhodaplatins were reduced (Fig. S41[Fn fn1]), indicating that rhodaplatins are sufficiently stable in the physiological environment but can be rapidly photoactivated in cells. The high stability of rhodaplatins in cells also ensures the colocalization of RhB and the Pt center.

**Fig. 3:**
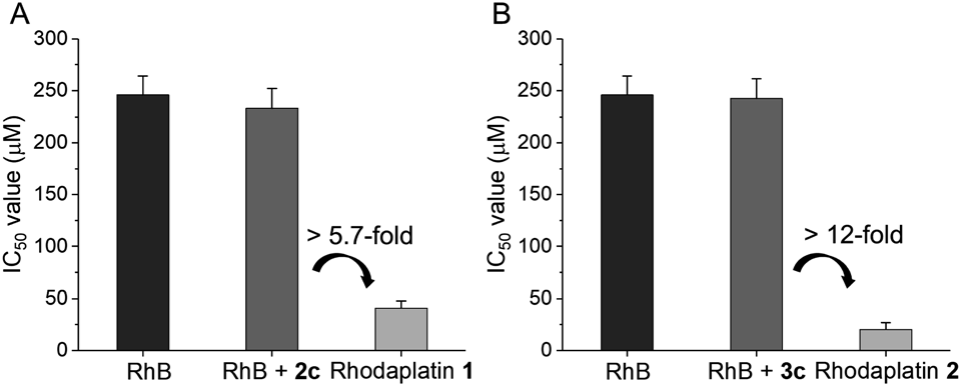
The IC_50_ value of (A) RhB, RhB + **2c**, and rhodaplatin **1**; (B) RhB, RhB + **3c**, and rhodaplatin **2** in A2780cisR cells upon irradiation with visible light. Cells were treated with complexes for 6 h, then the culture medium was replaced with fresh medium. Cells were irradiated with visible light (400760 nm, 4 mW cm^2^) for 30 min, and cultured for another 42 h. The IC_50_ value was determined by MTT assay.

Next, we compared the photocytotoxicity of free RhB, a mixture of RhB and Pt(iv), and rhodaplatins in A2780cisR platinum-resistant ovarian cancer cells. No significant change in cell viability was observed after irradiation (Fig. S42[Fn fn1]). RhB presented poor photocytotoxicity with an IC_50_ value of 2.46 10^4^ M (Fig. 3). Compared with free RhB, a mixture of RhB and Pt(iv) did not result in increased photocytotoxicity, suggesting that the Pt(iv) substrates cannot be efficiently photocatalyzed to their Pt(ii) counterparts in cancer cells. In contrast, significantly enhanced photocytotoxicity was achieved for rhodaplatins, and the IC_50_ values of rhodaplatin **1** and rhodaplatin **2** were 5.7- and 12-fold lower than that of the RhB plus Pt(iv) platform, indicating the effective intracellular activation of rhodaplatins. Compared with Pt(ii) drugs, rhodaplatins also displayed significantly enhanced photocytotoxicity (Table 1 and S3[Fn fn1]). We observed a similar effect in A549cisR platinum-resistant lung cancer cells as well as cancer cells from other origins. For example, photoactivated rhodaplatins exhibited not only significantly increased photocytotoxicities than the parent Pt(ii) drugs but also a greater ability to overcome drug resistance, with the resistance factor (RF) values in the range of 0.8 to 1.1, suggesting rhodaplatins may possess a distinct mechanism of action to overcome developed drug resistance. Rhodaplatins showed negligible dark cytotoxicity in normal cells.

**Table 1:** The cytotoxicity of different complexes against various cancer cell lines. Cells were treated with complex for 6 h, then the culture medium was replaced with drug-free fresh medium, and cells were irradiated with or without (bold text) visible light (400760 nm, 4 mW cm^2^) for 30 min. Then cells were cultured for another 42 h. The IC_50_ value was determined by the MTT assay

Cell line	IC_50_ [M]				PI^c^	FI^d^	IC_50_ [M]			PI^c^	FI^d^
	Carboplatin	RhB	RhB + **2c**^b^	Rhodaplatin **1**			Oxaliplatin	RhB + **3c**^b^	Rhodaplatin **2**		
A2780	**301 28**	**258 19**	**242 19**	**220 17**	5.0	7.3	**68 6**	**263 18**	**108 9**	4.4	2.6
	322 33	222 14	204 19	44 5			64 6	235 9	25 2		
A2780cisR (RF)^a^	**>400**	**265 27**	**254 21**	**250 18**	6.1	>9.8	**187 19**	**284 17**	**136 13**	6.7	9.8
	>400 ()	246 18 (1.1)	234 19 (1.1)	41 5 (0.9)			199 21 (3.1)	242 19 (1.0)	20 7 (0.8)		
MCF-7	**>400**	**301 30**	**325 22**	**245 17**	3.2	>5.2	**113 13**	**311 22**	**133 11**	3.1	2.4
	>400	255 15	279 18	77 9			103 12	264 25	43 3		
A549	**>400**	**288 23**	**291 16**	**251 15**	4.4	>7.0	**95 12**	**277 20**	**104 15**	3.7	3.1
	>400	247 17	219 18	57 5			87 6	211 15	29 5		
A549cisR (RF)^a^	**>400**	**305 29**	**284 19**	**289 17**	4.7	>6.5	**212 9**	**274 17**	**142 10**	4.0	5.0
	>400 ()	265 19 (1.1)	228 19 (1.0)	61 6 (1.1)			218 10 (2.5)	232 16 (1.1)	33 5 (1.1)		
HCT116	**>400**	**246 24**	**257 20**	**248 18**	5.3	>10.7	**58 4**	**239 26**	**112 6**	6.3	3.4
	>400	184 16	201 17	47 5			60 4	163 16	18 1		
MRC-5	**>400**	**262 32**	**277 21**	**>300**			**82 7**	**242 19**	**116 7**		

a Resistance factor (RF): the IC_50_ in A2780cisR (A549cisR) cells under irradiation/the IC_50_ in A2780 (A549) cells under irradiation.

b The IC_50_ values of free complex **2c** or **3c** are >200 M in all the tested cells.

c Phototoxic index (PI): the IC_50_ of the dark group treated with rhodaplatin/the IC_50_ of the irradiation group treated with rhodaplatin.

d Fold increase (FI): the IC_50_ of carboplatin (or oxaliplatin) of irradiation group/the IC_50_ of rhodaplatin **1** (or rhodaplatin **2**) of irradiation group.

**Fig. 4:**
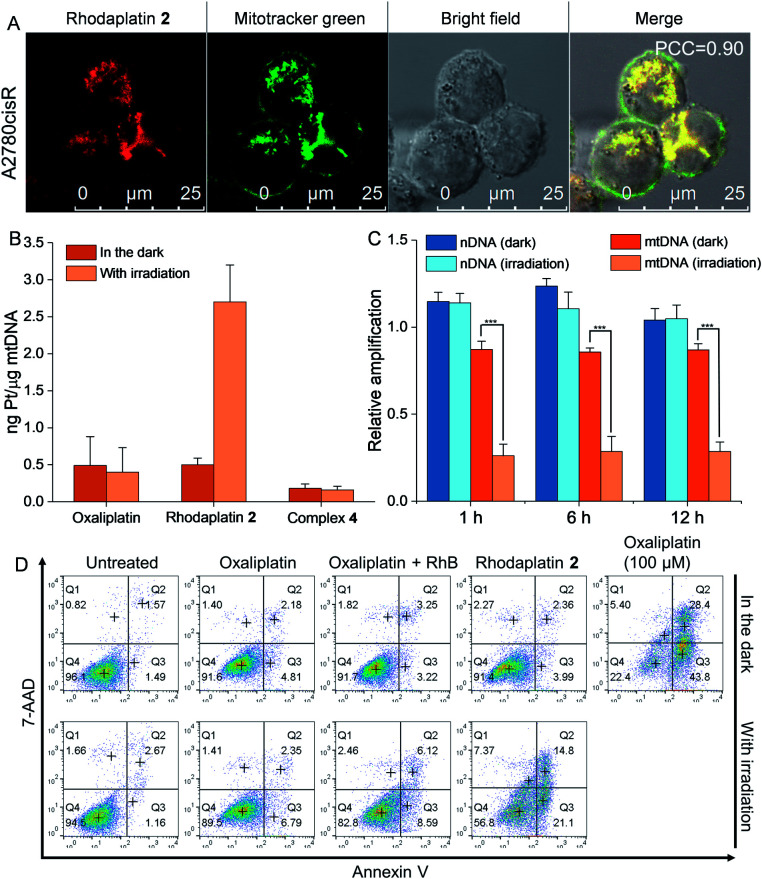
Rhodaplatin **2** effectively accumulated in the mitochondria and induced mitochondrial DNA damage after photoactivation. (A) The cellular distribution of rhodaplatin **2** in A2780cisR cells. Cells were treated with rhodaplatin **2** (10^5^ M) for 6 h, then co-stained with mitotracker. (B) The PtmtDNA binding amount in A2780cisR cells after different treatments. Cells were treated with complexes (10^5^ M) for 6 h, then the culture medium was replaced, and cells were irradiated with or without visible light (400760 nm, 4 mW cm^2^) for 30 min and further cultured for 1 h. (C) The relative amplification of nDNA and mtDNA in A2780cisR cells treated with rhodaplatin **2**. Cells were treated with complex (10^5^ M) for 6 h, then the culture medium was replaced with fresh medium, and irradiated with or without visible light (400760 nm, 4 mW cm^2^) for 30 min. After irradiation, cells were cultured for another 1, 6, and 12 h. (D) The apoptosis level of A2780cisR cells after different treatments. Cells were treated with complex (10^5^ M) for 6 h, then the medium was replaced with fresh medium, and cells were irradiated with or without visible light (400760 nm, 4 mW cm^2^) for 30 min and cultured for another 12 h. As a positive control, cells were treated with oxaliplatin (10^4^ M) for 6 h, then the medium was replaced with fresh medium, and cells were cultured for another 12 h.

As rhodaplatin **2** exhibited higher photocytotoxicity in the tested cell lines and possessed greater potential to overcome drug resistance in both monolayer and 3D tumor spheroid models (Fig. S43[Fn fn1]), we further explored its mechanism of action to overcome drug resistance. Rhodaplatin **2** presented considerable fluorescence in an aqueous solution (Fig. S37[Fn fn1]), which enabled us to monitor its subcellular distribution. Since rhodaplatin **2** is presented as a lipophilic cation, which may easily cross the phospholipid bilayers and accumulate in the mitochondria or endoplasmic reticulum (ER),^21^ we treated the cells with rhodaplatin **2** and co-stained the cells with fluorescent trackers of the mitochondria and ER. As shown in Fig. 4A and S44,[Fn fn1] the Pearson's colocalization coefficient (PCC) values of the mitochondrial- and ER-trackers with rhodaplatin **2** are 0.90 and 0.73, respectively. The prodrug showed a similar subcellular distribution tendency in MCF-7 cells (Fig. S45 and S46[Fn fn1]), indicating its strong mitochondria-targeting ability. As Pt-based drugs are well-known DNA damaging agents,^22^ we measured the interaction of rhodaplatin **2** with nuclear DNA (nDNA) and mitochondrial DNA (mtDNA). For rhodaplatin **2**- and oxaliplatin-treated cells, the amount of Pt on nDNA is 0.084 and 0.72 ng Pt per g DNA, respectively (Fig. S47[Fn fn1]); no nDNA damage response was triggered in the rhodaplatin **2**-treated cells (Fig. S48[Fn fn1]), indicating that nDNA is not the target. In contrast, photoactivated rhodaplatin **2** caused a much higher level of PtmtDNA binding (Fig. 4B) and greater mtDNA damage (Fig. 4C and S49[Fn fn1]) than that of oxaliplatin. Notably, although complex **4** (Fig. S50[Fn fn1]), another oxaliplatin-based Pt(iv) prodrug containing triphenylphosphonium (TPP) as the mitochondria-targeting group,^23^ exhibited comparable mitochondrial accumulation efficiency to that of rhodaplatin **2** (Fig. S51[Fn fn1]), complex **4** induced much lower PtmtDNA binding amount (0.16 ng Pt per g mtDNA) than rhodaplatin **2** (2.7 ng Pt per g mtDNA, Fig. 4B). Moreover, complex **4** was found to be nontoxic towards cancer cells (Table S4[Fn fn1]), suggesting that it was not sufficiently activated in cells, thus emphasizing the importance of developing targeted Pt drugs with controllable activation properties. Subsequently, the loss of mitochondrial membrane potential, a major event after intense mtDNA damage,^24^ was also detected in the cells treated with photoactivated rhodaplatin **2** but not oxaliplatin (Fig. S52[Fn fn1]). Following this observation, apoptosis-inducing factor (AIF) and endonuclease G (endo G), two important apoptogenic factors that respond to mitochondrial damage,^25^ were translocated from the mitochondria to the nucleus (Fig. S53 and S54[Fn fn1]), resulting in chromatin condensation (Fig. S55[Fn fn1]).^26^ At the same time, the photoactivated rhodaplatin **2** triggered the release of cytochrome c (Fig. S56[Fn fn1]), an essential mitochondrial factor for intrinsic apoptosis,^27^ and activated caspase-3 and -7 (Fig. S57[Fn fn1]), the key mediators responsible for mitochondria-mediated apoptosis,^28^ indicating the initiation of apoptosis. As expected, photoactivated rhodaplatin **2** induced a remarkably higher level of apoptosis than oxaliplatin in A2780cisR cells (Fig. 4D). Both activation of caspase-3/7 and nuclear fragmentation could be diminished by co-treatment with the apoptosis inhibitor Z-VAD-FMK (Fig. S57 and S58[Fn fn1]). These data confirmed that rhodaplatin **2** could induce mtDNA damage and activate the nDNA-damage-independent intrinsic apoptosis to overcome drug resistance.

## Conclusion

In summary, we developed a couple of photoactivable Pt(iv) prodrugs that could be effectively converted from their inert Pt(iv) state to clinically active Pt(ii) drugs *via* internal photoswitch in physiological conditions. Compared with the conventional photocatalyst plus Pt(iv) substrate platform, our rhodaplatins possess significantly closer proximity between the photoswitch ligand and Pt(iv) center by a covalent bond, enabling up to 4.8 10^4^-fold increased photoconversion efficiency and significantly increased photocytotoxicity in cancer cells. After the cellular entrance, rhodaplatin **2** effectively accumulated in the mitochondria of cancer cells, and induced mtDNA but not nDNA damage after visible light irradiation. Subsequently, the intense mtDNA damage led to the loss of mitochondrial membrane potential and the release of pro-apoptotic factor endoG, AIF, and cytochrome c in a nDNA-damage-independent manner. These translocated factors further triggered the condensation of chromatin and activation of caspase-3/7 to initiate apoptosis and overcome drug resistance (Fig. S59[Fn fn1]). Taken together, our results suggest that the distance between photocatalyst and Pt(iv) substrate is the bottleneck for conventional photocatalysis platforms; shortening the distance by integration in the same molecule can significantly improve the photoconversion efficiency. In addition, this study may pave the way to design highly effective platinum-based prodrugs with built-in photoswitches,^29^ and explore their potential applications for cancer treatment, especially against drug-resistant cancers by activating nDNA-damage-independent pathways.

## Author contributions

Z. D. and G. Z. designed the study. Z. X., H. Y., Q. Z., and Z. D. synthesized these complexes. Z. D. performed the experiments. S. C. and Z. D. detected the photo properties of rhodaplatin. C. L. and Z. D. completed cell-based experiments. H. H. carried out the DFT calculation. Z. W. carried out the EPR experiments. Z. D. and G. Z. analyzed the data. Z. D. and G. Z. wrote the paper. All authors edited and approved the final manuscript.

## Conflicts of interest

There are no conflicts to declare.

## Supplementary Material

SC-012-D0SC06839J-s001
