# Supplementary material for: An intramolecular photoswitch can significantly promote photoactivation of Pt(iv) prodrugs
Source: Chem Sci. 2021 Apr 1;12(19):6536–42. doi: 10.1039/d0sc06839j (PMC8139284; doi:10.1039/d0sc06839j)
Supplement: SC-012-D0SC06839J-s001 [file SC-012-D0SC06839J-s001.pdf]

Electronic Supplementary Material (ESI) for Chemical Science.
This journal is © The Royal Society of Chemistry 2021

Supporting Information

An Intramolecular Photoswitch Can Significantly Promote Photoactivation of Pt(IV) Prodrugs

Zhiqin Deng, Cai Li, Shu Chen, Qiyuan Zhou, Zoufeng Xu, Zhigang Wang, Houzong Yao, Hajime Hirao, and Guangyu Zhu*

Contents

Experimental Procedures 2
- Materials, instruments, and reagents 2
- Synthesis and characterization 3
- Photocatalysis experiment 6
- Dark stability of rhodaplatin 7
- Photo-reduction of rhodaplatin 8
- Stability and photoactivation of rhodaplatin in cancer cells 8
- Stoichiometry and association constant of rhodaplatin towards ascorbate 8
- Electron paramagnetic resonance (EPR) spectra of ascorbate radical 9
- DFT calculation 9
- Cell culture 13
- Cytotoxicity 13
- Annexin V/7-AAD double staining assay 14
- Cytotoxicity in tumor spheroids 14
- Cellular accumulation 15
- Mitochondrial accumulation 15
- Platinum accumulation in mitochondrial and nuclear DNA 15
- Mitochondrial and nuclear DNA damage 16
- JC-1 staining 16
- Hoechst staining 17
- Translocation of AIF and endoG 17
- Caspase activation test 18
- Western blot 18
- Statistical analysis 19

References 60

Experimental Procedures

Materials, instruments, and reagents

Unless otherwise noted, all the reactions were carried out under normal atmospheric conditions with protection from light. All chemicals and solvents were purchased from commercial resources. Rhodamine B (R6626), thiazolyl blue tetrazolium bromide (M2128), trichloroacetic acid (T6399), and sulforhodamine B (230162) were purchased from Sigma-Aldrich. Dulbecco's modified eagle medium (DMEM), minimum essential medium (MEM), Roswell park memorial institute (RPMI) 1640 medium, trypsin, phosphate buffered saline (PBS), and fetal bovine serum (FBS) were obtained from Life Technologies. Unless otherwise noted, the components of PBS buffer used in this study are 10 mM Na_2HPO_4, 2 mM KH_2PO_4, 137 mM NaCl, 2.7 mM KCl, the pH is 7.4, and the temperature is 310 K.

Unless otherwise noted, the light source used in this study is a LED white light (400-760 nm, 4 mW cm^{-2}). NMR data were recorded with Bruker Ascend AVANCE III 300 MHz, 400 MHz, and 600 MHz spectrometers at room temperature and protect from light. The ESI-MS data were recorded with a Liquid Chromatograph-Mass Spectrometer (API-3200 Triple-Q MS/MS). The High-resolution MS data were recorded with a Bruker Daltonics micrOTOF. Electron paramagnetic resonance studies were carried out in the X-Band using a Bruker EMXplus EPR spectrometer (Bruker, Rheinstetten, Germany). The confocal image was recorded with a Laser Confocal Scanning Microscope (Leica SPE and Leica SP5). The HPLC analysis was carried out by Phenomenex 00G-4435-E0 Gemini 5u C18 110A 250 x 4.6 mm HPLC Column on a Shimadzu LC-20A HPLC instrument.

GeneJET Genomic DNA Purification Kit (K0722), Mitochondria Isolation Kit for Cultured Cells (89874), CellEvent™ Caspase-3/7 Green Detection Reagent (C10423), MitoTracker™ Green (M7514), ER-Tracker™ Blue-White DPX (E12353), JC-1 Dye (T3168), and Quant-iT™ PicoGreen™ dsDNA Assay Kit (P11496) were purchased from ThermoFisher. Rabbit monoclonal anti-Histone H2A.X (D17A3) XP® antibody (#7631), mouse anti-cytochrome c antibody (#12963), and rabbit monoclonal anti-β-actin antibody (#4970) were purchased from Cell Signaling Technology (CST). Rabbit monoclonal anti-AIF antibody (ab1998) and rabbit monoclonal anti-Endo G antibody (ab9647) were purchased from Abcam. Z-VAD-FMK caspase inhibitor was obtained from Beyotime (China).

Synthesis and characterization

Synthesis of *c,c,t*-[Pt(NH_3)$_2$$Cl_2(OH)_2$] (1a): Complex **1a** was synthesized according to other report.[1] Cisplatin was suspended in Milli-Q water (15 mL), hydrogen peroxide (50% in H_2O, 3 mL) was added to stir at 50 ºC for 5 h. After the reaction, the solution was cool to 4 ºC to precipitate the product. The formed product was collected by centrifugation, and dried by lyophilization. Yield: 93%. Purity: 98%.

Synthesis of Ac-cisPt(IV)-Ac (1b): Complex **1b** was synthesized according to our previous report.[2] Cisplatin (1 g, 3.3 mmol, 1.0 eq.) was suspended in acetic acid (400 mL), then hydrogen peroxide (35% in H_2O, 12 mL) was added. The mixture was stirred at room temperature for 5 h. The reaction mixture was centrifuged, the supernatant was concentrated by rotary evaporation. Ethyl ether was added to precipitate the crude product. The solid was washed with acetone and dichloromethane to give complex **1b**. Yield: 86%. Purity: 97%. ^{1}H NMR (400 MHz, DMSO-d_6, δ): 6.13 (m, J = 49.6 Hz, 6H; NH_3), 1.89 (s, 3H; CH_3). ^{13}C NMR (151 MHz, DMSO-d_6, δ): 178.96, 24.20. ^{195}Pt NMR (129 MHz, DMSO-d_6, δ): 1051.2.

Synthesis of Ac-cisPt(IV)-COOH (1c): Complex **1c** was synthesized according to our previous report.[2] Cisplatin (1 g, 3.3 mmol, 1.0 eq.) was suspended in acetic acid (400 mL) before hydrogen peroxide (50% in H_2O, 10 mL, 176.4 mmol, 53.0 eq.) was added. The mixture was stirred at room temperature for 4 h. The reaction mixture was centrifuged to remove unreacted cisplatin. The supernatant was concentrated under reduced pressure. Excess ethyl ether was added to the residue to precipitate a yellow solid. The formed solid was collected by centrifugation and washed by acetone, dichloromethane (DCM), and ethyl ether respectively to get yellow solid (980 mg). This yellow solid (300 mg, 0.8 mmol) was suspended in DMF (2 mL). Succinic anhydride (480 mg, 4.8 mmol) was then added. The mixture was stirred at 60 ºC overnight. DCM (2 mL) and ethyl ether (90 mL) were added to the reaction mixture to form a light-yellow solid. The solid was collected by centrifugation and washed by DCM (2 mL) and ethyl ether (10 mL) three times to get light yellow solid (260 mg). Yield: 68%. Purity: 95%. ^{1}H NMR (400 MHz, DMSO-d_6, δ): 12.05 (s, 1H; COOH), 6.51 (s, 6H; 2NH_3), 2.48 – 2.29 (m, 4H; 2CH_2), 1.90 (s, 3H; CH_3). ^{13}C NMR (151 MHz, DMSO-d_6, δ): 179.75, 178.65, 173.19, 30.86, 30.01, 23.28. ^{195}Pt NMR (129 MHz, DMSO-d_6, δ): 1229.4. ESI-MS: m/z [M - H]$^{-}$ calculated for $C_6H_{13}Cl_2N_2O_6Pt$: 475.2, found 475.2.

Synthesis of OH-carPt(IV)-OH (2a): Carboplatin (500 mg) was suspended in Milli-Q water (15 mL), hydrogen peroxide (50% in H_2O, 3 mL) was added to stir at 50 °C for 5 h. After the reaction, the solution was cooled to 4 °C to precipitate the product. The formed product was

collected by centrifugation, and dried by lyophilization. Yield: 87%. Purity: 98% ESI-MS: m/z $[M - H]^-$ calculated for $C_6H_{13}N_2O_6Pt$: 404.2, found: 404.1.

Synthesis of Ac-carPt(IV)-OH (2b): Carboplatin (200 mg, 0.54 mmol) was suspended in acetic acid (20 mL), to which 30% hydrogen peroxide aqueous solution (0.5 mL) was added. The mixture was stirred for 1 h at room temperature. Vaporize the solvent by a rotate evaporator and wash the residue with acetone (10 mL) and diethyl ether (10 mL). Dry the white solid in vacuum. Yield: 72%. Purity: 98%. ^1H NMR (400 MHz, DMSO-d_6, δ): 5.86 (dq, J = 52.8, 27.3, 26.6 Hz, 6H; $2NH_3$), 2.51 (m, 4H; $2CH_2$), 1.91 (s, 3H; CH_3), 1.78 (q, J = 7.8 Hz, 2H; CH_2). ^{13}C NMR (151 MHz, DMSO-d_6, δ): 178.29, 176.98, 56.17, 40.56, 32.30, 31.79, 23.89, 16.32. ^{195}Pt NMR (129 MHz. DMSO-d_6, δ): 1754.2.

Synthesis of Ac-carPt(IV)-COOH (2c): Ace-CarPt-OH (100 mg, 0.22 mmol) was dissolved in dry dimethyl sulfoxide (1 mL), to which succinic anhydride (26.4 mg, 0.26 mmol) was added. Stir the mixture overnight at 50 °C. The desired product was precipitated with dichloromethane (3 mL) and diethyl ether (40 mL). The white solid was collected by centrifuge and dried in vacuum. Yield: 39%. Purity: 95%. ^1H NMR (300 MHz, DMSO-d_6, δ): 12.05 (s, 1H; COOH), 6.35 (m, 6H; $2NH_3$), 2.61 (d, J = 5.5 Hz, 2H; CH_2), 2.51 (m, 4H; $2CH_2$), 2.37 (q, J = 7.2, 5.9 Hz, 2H; CH_2), 1.91 (s, 3H; CH_3), 1.80 (q, J = 8.2 Hz, 2H; CH_2). ^{13}C NMR (151 MHz, DMSO-d_6, δ): 179.15, 177.74, 176.71, 174.17, 56.00, 31.96, 31.67, 31.41, 30.62, 30.11, 23.01, 22.97, 16.19. ^{195}Pt NMR (129 MHz, DMSO-d_6, δ): 1950.9.

Synthesis of *c,t*-[Pt(DACH)ox(OH)₂] (3a): Complex **3a** was synthesized according to another report.[3] Oxaliplatin (500 mg) was suspended in Milli-Q water (15 mL), hydrogen peroxide (50% in H_2O, 3 mL) was added to stir at 50 °C for 5 h. After the reaction, the solution was cool to 4 ºC to precipitate the product. The formed product was collected by centrifugation, and dried by lyophilization. Yield: 91%. Purity: 97% ESI-MS: m/z $[M - H]^-$ calculated for $C_8H_{15}N_2O_6Pt$: 430.3, found: 430.3.

Synthesis of Ac-oxaPt(IV)-OH (3b): Complex **3b** was synthesized according to another report.[3] Oxaliplatin (1 g, 2.52 mmol, 1 eq.) was suspended in acetic acid (200 mL) before hydrogen peroxide (50% in H_2O, 5 mL) was added. The mixture was stirred at room temperature for 3 h. The reaction mixture was concentrated under reduced pressure. Excess ethyl ether was added to the residue to precipitate a white solid. The formed solid was collected by centrifugation and washed by acetone, DCM, and Et_2O respectively to get white solid (1.01 g). Yield: 85%. Purity: 96%. ^1H NMR (400 MHz, DMSO-d_6, δ): 8.58 (s, 1H; NH_2), 8.18 (s, 1H; NH_2), 7.82 (s, 1H; NH_2), 7.12 (s, 1H; NH_2), 2.46 (s, 2H; CH_2), 2.13 – 1.96 (m, 2H; CH_2), 1.91 (s, 3H; CH_3), 1.58 – 1.25 (m, 4H; $2CH_2$), 1.12 (d, J = 9.2 Hz, 2H; CH_2). ^{13}C NMR (151 MHz,

DMSO-d_6, δ): 179.15, 163.18, 60.77, 59.78, 30.25, 30.09, 23.34, 23.15, 22.99. ^{195}Pt NMR (129 MHz, DMSO-d_6, δ): 1408.8. ESI-MS: m/z [M - H]$^-$ calculated for $C_{10}H_{17}N_2O_7Pt$: 472.3, found: 472.1.

Synthesis of Ac-oxaPt(IV)-COOH (3c): Complex **3c** was synthesized according to another report.[3] Ac-oxaPt(IV)-OH (300 mg, 0.6 mmol, 1 eq.) was suspended in DMF (2 mL). Succinic anhydride (360 mg, 3.6 mmol, 6 eq.) was then added. The mixture was stirred at 60 °C overnight. DCM (2 mL) and ethyl ether (90 mL) were added to the reaction mixture to form a white solid. The solid was collected by centrifugation and washed by DCM (2 mL) and ethyl ether (10 mL) for three times to get white solid (269 mg). Yield: 73%. Purity: 96%. ^{1}H NMR (400 MHz, DMSO-d_6, δ): 12.11 (s, 1H; COOH), 8.65 – 7.88 (m, 4H; 2NH_2), 2.63 – 2.53 (m, 2H; CH_2), 2.50 – 2.31 (m, 4H; 2CH_2), 2.10 (s, 2H; CH_2), 1.95 (s, 3H; CH_3), 1.59 – 1.28 (m, 4H; CH_2), 1.24 – 1.06 (m, 2H; CH_2). ^{13}C NMR (151 MHz, DMSO-d_6, δ): 180.05, 179.05, 174.16, 163.85, 163.79, 61.43, 61.35, 31.34, 31.01, 30.04, 24.01, 23.43. ^{195}Pt NMR (129 MHz, DMSO-d_6, δ): 1613.2.

Synthesis of rhodaplatin 1: Rhodamine B (480 mg), N-hydroxysuccinimide (NHS, 173 mg) and dicyclohexylcarbodiimide (DCC, 309 mg) were dissolved in acetonitrile (ACN,10 mL) to stir at room temperature for 12 h. After reaction, the dicyclohexylurea (DCU) was removed by centrifugation and the solution was carefully collected. The ACN was removed by rotary evaporation, the NHS ester of rhodamine B was collected. The rhodamine B NHS ester (90 mg) and diammine (cyclobutane-1,1 dicarboxylato) dihydroxido platinum(IV) (40 mg) were mixed in DMSO (3 mL) to stir at 50 °C for 6 h. Then DCM (10 mL) and Et_2O (30 mL) were added to precipitate the crude product, rhodaplatin **1** was further purified by HPLC. Yield: 31%. Purity: 97%. ^{1}H NMR (300 MHz, D_2O, δ): 8.02 (dd, J = 8.3, 1.3 Hz, 1H; Ar H), 7.64 (dd, J = 12.7, 7.1 Hz, 2H; Ar H), 7.26 (d, J = 8.1 Hz, 1H; Ar H), 6.93 (d, J = 9.4 Hz, 2H; Ar H), 6.72 (d, J = 9.4 Hz, 2H; Ar H), 6.57 (d, J = 2.3 Hz, 2H; Ar H), 3.47 (dq, J = 15.7, 7.3 Hz, 8H; 4CH_2), 2.27 (dt, J = 17.0, 8.1 Hz, 4H; 2CH_2), 1.77 (d, J = 8.5 Hz, 2H; CH_2), 1.12 (t, J = 7.0 Hz, 12H; 4CH_3). ^{13}C NMR (151 MHz, D_2O, δ): 179.09, 175.22, 158.02, 156.88, 155.22, 132.70, 132.04, 130.95, 130.10, 113.89, 112.70, 96.19, 54.63, 45.52, 35.31, 29.28, 28.83, 15.48, 11.95. ^{195}Pt NMR (129 MHz. D_2O, δ): 1739.1. ESI-HRMS: m/z [M - Cl$^-$]$^+$ calculated for $C_{34}H_{43}N_4O_8Pt$: 830.2723, found: 830.2725.

Synthesis of rhodaplatin 2: Rhodamine B (480 mg), N-hydroxysuccinimide (NHS, 173 mg) and dicyclohexylcarbodiimide (DCC, 309 mg) were dissolved in ACN (10 mL) to stir at room temperature for 12 h. After reaction, the DCU was removed by centrifugation and the solution was carefully collected. The ACN was removed by rotary evaporation, the NHS ester of

rhodamine B was collected. The crude rhodamine B NHS ester (90 mg) and c,t-[Pt(DACH)ox(OH)$_2$] (DACH = (1R,2R)-1,2-diaminocyclohexane, ox = oxalate) (43 mg) were mixed in DMSO (4 mL) to stir at 50 °C for 6 h. Then DCM (10 mL) and Et_2O (30 mL) were added to precipitate the crude product, rhodaplatin **2** was further purified by HPLC. Yield: 22%. Purity: 97%. ^{1}H NMR (400 MHz, D_2O, δ): 7.92 (d, *J* = 7.8 Hz, 1H; Ar H), 7.73 (t, *J* = 7.8 Hz, 1H; Ar H), 7.64 (t, *J* = 7.8 Hz, 1H; Ar H), 7.31 (d, *J* = 7.6 Hz, 1H; Ar H), 6.92 (dd, *J* = 9.4, 7.0 Hz, 2H; Ar H), 6.68 (t, *J* = 7.4 Hz, 2H; Ar H), 6.55 – 6.47 (m, 2H; Ar H), 3.41 (dt, *J* = 14.6, 7.4 Hz, 8H; 4CH_2), 2.52(td, *J*=11.8, 4.3 Hz, 1H; CH) 2.29 (td, *J* = 11.6, 3.9 Hz, 1H; CH), 1.97 (t, *J* = 14.2 Hz, 2H; CH_2), 1.41 (s, 4H; 2CH_2), 1.08 (q, *J* = 8.2, 7.6 Hz, 12H; 4CH_3), 0.98 – 0.83 (m, 2H; CH_2). ^{13}C NMR (151 MHz, D_2O, δ): 164.53, 157.16, 155.44, 155.34, 132.12, 130.82, 130.76, 130.34, 130.14, 114.06, 113.96, 96.45, 96.35, 95.82, 62.35, 62.15, 60.81, 45.63, 31.62, 30.95, 23.86, 23.42, 23.22, 11.90. ^{195}Pt NMR (129 MHz, D_2O, δ): 1403.8. ESI-HRMS: m/z [M - Cl^-]$^+$ calculated for $C_{36}H_{45}N_4O_8Pt$: 856.2879, found: 856.2872.

Synthesis of TPP-oxaPt(IV)-OH (4): (2-Carboxyethyl)triphenylphosphonium bromide (200 mg, 0.48 mmol) and N-hydroxysuccinimide (66 mg, 0.58 mmol) was dissolved in dry N,N-dimethylacetamide (2 mL), to which dicyclohexylcarbodiimide (119 mg, 0.58 mmol) was added. The reaction mixture was stirred at 60 °C overnight. The clear liquid was precipitated with dichloromethane (3 mL) and diethyl ether (40 mL). After drying in a vacuum, the solid was dissolved in DMSO (1 mL), to which dihdroxido oxaliplatin (1.5 equiv.) was added. The mixture was stirred for 3 h at 60 °C. The clear liquid is collected by centrifuge and the desired product was crystallized with dichloromethane (3 mL) and diethyl ether (40 mL). Pale orange solid obtains after freeze-drying. Purity: 95%. ^{1}H NMR (600 MHz, DMSO-d_6, δ): 8.03 – 7.74 (m, 18H; Ar H and NH_2), 7.19 (s, 1H; NH_2), 3.83 – 3.71 (m, 2H; CH_2), 2.60 (s, 2H; CH_2), 2.14 – 1.95 (m, 2H; CH_2), 1.61 – 1.26 (m, 4H; 2CH_2), 1.10 (dt, *J* = 29.3, 13.3 Hz, 2H; CH_2). ^{31}P NMR (243 MHz, DMSO-d_6, δ): 24.3. ^{195}Pt NMR (129 MHz, DMSO-d_6, δ): 1419.2.

Photocatalysis experiment

Rhodamine B, Pt(IV) complex, and sodium ascorbate at the specified concentration were mixed in PBS buffer (pH 7.4, 10 mL). The mixed solution was separated into 2 tubes. The first tube was placed in the dark for 5 h; the second tube was irradiated by visible light (400-760 nm, 4 mW cm^{-2}) for 5 h. The tubes containing the solutions were placed in a water-cool system, and the water is from a water bath (set at 310 K) to keep the temperature constant. At each time point, the solution of both tubes was analyzed by HPLC, the spectrum from 190 nm to 700 nm was scanned by a photodiode array (PDA) detector. According to the UV-Vis spectrum,

complexes **2a**, **2b**, **2c**, and carboplatin have the same molar extinction coefficient at the wavelength of 311 nm. Similarly, complexes **3a**, **3b**, **3c**, and oxaliplatin share the same molar extinction coefficient at the wavelength of 303 nm. Therefore, at such wavelength, the peak area of carboplatin (or oxaliplatin) equals to the peak area of corresponding reduced Pt(IV). Thus, the percentage of reduced complexes **2a**, **2b**, or **2c** can be calculated by the following equation:

Percentage of reduced Pt(IV) = $A_{II}/(A_{II}+A_{IV}) \times 100\%$

A_{IV} is the peak area of **2a**, **2b**, or **2c** recorded at the wavelength of 311 nm; A_{II} is the peak area of carboplatin recorded at the wavelength of 311 nm.

Similarly, the percentage of reduced complexes **3a**, **3b**, or **3c** is calculated by the following equation:

Percentage of reduced Pt(IV) = $A'_{II}/(A'_{II}+A'_{IV}) \times 100\%$

A'_{IV} is the peak area of **3a**, **3b**, or **3c** recorded at the wavelength of 303 nm; A'_{II} is the peak area of oxaliplatin recorded at the wavelength of 303 nm.

The turnover frequency (TOF) is calculated according to the following equation:

TOF = (molar of reduced Pt(IV)) / [(molar of catalyst)×time]

The conversion rate of RhB and rhodaplatins is calculated according to the following equation:

Conversion rate = molar of reduced Pt(IV) / time

The TOF value and conversion rate were calculated at the time point that reaction presented the highest conversion rate. For rhodaplatins, 0.5 min was chosen for calculation. For the "RhB+Pt(IV)" platform, 5 h was chosen for calculation.

Dark stability of rhodaplatin

Rhodaplatin was dissolved in PBS buffer (10 mM Na_2HPO_4, 2 mM KH_2PO_4, 137 mM NaCl, 2.7 mM KCl, pH = 7.4) at a final concentration of 100 μM with or without the presence of 2 mM ascorbate or glutathione and incubated at 37 °C. At certain time points, the sample was analyzed by HPLC. The HPLC program was set as: 0-20% phase B (linear increase from 0-8 min), 20-90% phase B (linear increase from 8-18 min), 90% phase B (18-20 min), 90-0% phase B (20-25 min). Phase A is water with 0.1% formic acid, and phase B is acetonitrile with 0.1% formic acid. The signal was recorded at 254 nm.

Photo-reduction of rhodaplatin

Rhodaplatin was dissolved in PBS buffer (10 mM Na_2HPO_4, 2 mM KH_2PO_4, 137 mM NaCl, 2.7 mM KCl, pH = 7.4) at a final concentration of 100 μM in the presence of 2 mM ascorbate or glutathione and incubated at 37 °C. The solution was irradiated with white light (4 mW cm^{-2}) and analyzed at different time points by HPLC.

Stability and photoactivation of rhodaplatin in cancer cells

A2780cisR cells (10,000 cells per well) were cultured in 2 cm dishes for 48 h, then cells were cultured with medium containing 50 μM rhodaplatins for 6 h. After incubation, the medium was taken out, after centrifugation (12,000 g) for 10 min, the supernatant was analyzed by HPLC. Cells were washed by PBS twice and cultured with drug-free medium, then cells were irradiated with or without white light (400-760 nm, 4 mW cm^{-2}) for 30 min. After that, cells were lysed by 100 μL lysis buffer (5 mM Tris, 5 mM EDTA, 150 mM NaCl, 1% Triton-X 100) for 20 min on ice. Then the lysate was centrifugated at 12, 000 g for 10 min, the supernatant was analyzed by HPLC. For the control experiment, rhodaplatins or RhB was directly added into the culture medium, and analyzed by HPLC after 10 min centrifugation (12,000 g). To bypass the signal peaks in the lysate, the HPLC was recorded at 560 nm.

Stoichiometry and association constant of rhodaplatin towards ascorbate

The stoichiometry of rhodaplatin-ascorbate ion pair was detected by Job's continuous variation method.[4] The stock solutions of rhodaplatin **2** (100 μM) and sodium ascorbate (100 μM) were prepared in Milli-Q water, and then the two stock solutions were mixed with different ratios to give solutions that contain 0-100 μM rhodaplatin **2** and 100-0 μM sodium ascorbate, where the total concentration of rhodaplatin and ascorbate is kept at 100 μM. The UV-Vis spectrum of each solution was recorded. At the same time, solutions that only contained 0-100 μM rhodaplatin **2** were prepared, and the UV-Vis spectrum of these solutions was recorded as well.

Because rhodaplatin **2** presented maximum absorption at 561 nm, and there is no absorption for sodium ascorbate at this wavelength; therefore, the absorption change at 561 nm induced by rhodaplatin-ascorbate ion pair could be obtained by comparing the absorption of rhodaplatin solutions with or without ascorbate. The relationship between the change of absorption and mole fraction of rhodaplatin **2** was mapped, and the maximum absorption change was observed when the mole fraction of rhodaplatin **2** is 0.5, indicating the rhodaplatin and ascorbate is binding in a 1:1 mode.

The association constant was detected by UV-Vis spectroscopic and fluorescence spectroscopic titration according to a review article by Thordarson.[5] Briefly, rhodaplatin (100 μM) was dissolved in Milli-Q water and titrated with ascorbate solution (10 mM, dissolved in Milli-Q water, containing 100 μM rhodaplatin). After each addition, the mixed solution was stirred for 20 seconds. The change of UV-Vis spectra and fluorescence spectra during the titration was recorded. After titration, the solution was analyzed by HPLC to confirm that rhodaplatin was not reduced. The association constant was calculated following previous studies,[6-7] and the collected data were analyzed by online software http://supramolecular.org/ by applying a 1:1 global fitting model (Nelder-Mead method).

Electron paramagnetic resonance (EPR) spectra of ascorbate radical

Sodium ascorbate (20 mM) or a mixture of rhodaplatin **2** (1 mM) and sodium ascorbate (20 mM) were dissolved in PBS buffer (pH 7.4). The prepared solutions were transferred into EPR tubes, then the tubes were irradiated with or without white light (150W) for 1 min, and the EPR signal of ascorbate radicals was obtained on a Bruker EMXplus EPR spectrometer. The instrument settings for the cylindrical resonator were the following: microwave frequency 9.85 GHz, microwave power 2.0 mW, modulation frequency 100 kHz, modulation amplitude 0.1 mT, sweep width 4 mT, conversion time 75.00 ms, time constant 0.01 ms, sweep time 30.00 s.

DFT calculation

- Software: Gaussian Rev D.01[8]
- B3LYP/[SDD(Pt),6-31G*(others)] for geometry optimization (energy = E1, zero-point vibrational energy = ZPE) [9-15]
- B3LYP(SCRF,solvent=water)/def2-TZVP for energy calculation (energy = E2)[16-17]

Raw energy data

(1)

	E1 [hartrees]	ZPE [hartrees]	E2 [hartrees]	ΔE2 [kcal mol^{-1}]	Δ(E2+ZPE) [kcal mol^{-1}]
Pt(IV)-$(OH)_2$ (**3a**)	-994.751698	0.265876	-995.197656		
ASA(–)	-684.193405	0.137142	-684.619699		
sum	-1678.945103	0.403018	-1679.817355	0.0	0.0
Pt(III)-OH	-918.917095	0.250955	-919.325938		
ASA(•–)	-683.588148	0.124855	-684.009270		
H_2O	-76.407025	0.021139	-76.470400		
sum	-1678.912268	0.396949	-1679.805608	7.4	3.6
sum-H_2O	-1602.505243	0.375810	-1603.335208	7.4	3.6
oxaliplatin	-843.145076	0.238382	-843.518530		
DHA	-683.510069	0.125875	-683.840999		
OH^{-}	-75.716802	0.007701	-75.946985		
sum	-1602.371948	0.371958	-1603.306514	25.4	19.2

(2)

	E1 [hartrees]	ZPE [hartrees]	E2 [hartrees]	ΔE2 [kcal mol^{-1}]	Δ(E2+ZPE) [kcal mol^{-1}]
Pt(IV)-(OH)(OAc) (**3b**)	-1147.436495	0.304738	-1147.936466		
ASA(–)	-684.193405	0.137142	-684.619699		
sum	-1831.629900	0.441880	-1832.556165	0.0	0.0
Pt(III)-OAc	-1071.603519	0.289501	-1072.071098		
ASA(•–)	-683.588148	0.124855	-684.009270		
H_2O	-76.407025	0.021139	-76.470400		
sum	-1831.598692	0.435495	-1832.550767	3.4	-0.6
sum-H_2O	-1755.191667	0.414356	-1756.080368	3.4	-0.6
oxaliplatin	-843.145076	0.238382	-843.518530		
DHA	-683.510069	0.125875	-683.840999		
AcO^{-}	-228.493167	0.048295	-228.724645		
sum	-1755.148312	0.412552	-1756.084175	1.0	-4.1

(2’)

	E1 [hartrees]	ZPE [hartrees]	E2 [hartrees]	ΔE2 [kcal mol^{-1}]	Δ(E2+ZPE) [kcal mol^{-1}]
Pt(IV)-(OH)(OAc) (**3b**)	-1147.436495	0.304738	-1147.936466		
ASA(–)	-684.193405	0.137142	-684.619699		
sum	-1831.629900	0.441880	-1832.556165	0.0	0.0
Pt(III)-OH	-918.917095	0.250955	-919.325938		
ASA(•–)	-683.588148	0.124855	-684.009270		
AcOH	-229.077608	0.062048	-229.196331		
sum	-1831.582851	0.437858	-1832.531539	15.5	12.9
sum-AcOH	-1602.505243	0.375810	-1603.335208	15.5	12.9
oxaliplatin	-843.145076	0.238382	-843.518530		
DHA	-683.510069	0.125875	-683.840999		
OH^-	-75.716802	0.007701	-75.946985		
sum	-1602.371948	0.371958	-1603.306514	33.5	28.5

(3)

	E1 [hartrees]	ZPE [hartrees]	E2 [hartrees]	ΔE2 [kcal mol^{-1}]	Δ(E2+ZPE) [kcal mol^{-1}]
Pt(IV)-(OAc)($O_2CC_2H_4CO_2^-$) (**3c**)	-1527.473117	0.374929	-1528.189684		
ASA(–)	-684.193405	0.137142	-684.619699		
sum	-2211.666522	0.512071	-2212.809383	0.0	0.0
Pt(III)-OAc	-1071.603519	0.289501	-1072.071098		
ASA(•–)	-683.588148	0.124855	-684.009270		
$^-O_2CC_2H_4CO_2H$	-456.415739	0.091473	-456.729979		
sum	-2211.607405	0.505829	-2212.810346	-0.6	-4.5
sum-($^-O_2CC_2H_4CO_2H$)	-1755.191667	0.414356	-1756.080368	-0.6	-4.5
oxaliplatin	-843.145076	0.238382	-843.518530		
DHA	-683.510069	0.125875	-683.840999		
AcO^-	-228.493167	0.048295	-228.724645		
sum	-1755.148312	0.412552	-1756.084175	-3.0	-8.0

(3’)

	E1 [hartrees]	ZPE [hartrees]	E2 [hartrees]	ΔE2 [kcal mol^{-1}]	Δ(E2+ZPE) [kcal mol^{-1}]

Pt(IV)-(OAc)<br>($O_2CC_2H_4CO_2^-$)<br>(**3c**)<br>ASA(–)	-1527.473117<br>-684.193405	0.374929<br>0.137142	-1528.189684<br>-684.619699		
sum	-2211.666522	0.512071	-2212.809383	0.0	0.0
Pt(III)-<br>$O_2CC_2H_4CO_2^-$<br>ASA(•–)<br>AcOH	-1298.963903<br>-683.588148<br>-229.077608	0.321015<br>0.124855<br>0.062048	-1299.590283<br>-684.009270<br>-229.196331		
sum	-2211.629659	0.507918	-2212.795884	8.5	5.9
sum-AcOH	-1982.552051	0.445870	-1983.599553	8.5	5.9
oxaliplatin<br>DHA<br>$^-O_2CC_2H_4CO_2^-$	-843.145076<br>-683.510069<br>-455.687326	0.238382<br>0.125875<br>0.079894	-843.518530<br>-683.840999<br>-456.238761		
sum	-1982.342472	0.444151	-1983.598290	9.3	5.6

(4)

	E1 [hartrees]	ZPE [hartrees]	E2 [hartrees]	ΔE2 [kcal mol^{-1}]	Δ(E2+ZPE) [kcal mol^{-1}]
Rhodaplatin **2**<br>ASA(–)	-2339.015646<br>-684.193405	0.784100<br>0.137142	-2339.988892<br>-684.619699		
sum	-3023.209051	0.921242	-3024.608591	0.0	0.0
Pt(III)-Rhoda<br>ASA(•–)<br>H2O	-2263.183383<br>-683.588148<br>-76.407025	0.769008<br>0.124855<br>0.021139	-2264.124170<br>-684.009270<br>-76.470400		
sum	-3023.178556	0.915002	-3024.603839	3.0	-0.9
sum-H_2O	-2946.771531	0.893863	-2948.133440	3.0	-0.9
oxaliplatin<br>DHA<br>L	-843.145076<br>-683.510069<br>-1420.220341	0.238382<br>0.125875<br>0.528218	-843.518530<br>-683.840999<br>-1420.787192		
sum	-2946.875486	0.892475	-2948.146721	-5.4	-10.1

(4')

	E1 [hartrees]	ZPE [hartrees]	E2 [hartrees]	ΔE2 [kcal mol^{-1}]	Δ(E2+ZPE) [kcal mol^{-1}]

Rhodaplatin **2**<br>ASA(–)	-2339.015646<br>-684.193405	0.784100<br>0.137142	-2339.988892<br>-684.619699		
sum	-3023.209051	0.921242	-3024.608591	0.0	0.0
Pt(III)-OH<br>ASA(•–)<br>LH^+	-918.917095<br>-683.588148<br>-1420.647376	0.250955<br>0.124855<br>0.541321	-919.325938<br>-684.009270<br>-1421.249666		
sum	-3023.152619	0.917131	-3024.584874	14.9	12.3
sum-H_2O	-1602.505243	0.375810	-1603.335208	14.9	12.3
oxaliplatin<br>DHA<br>OH^-	-843.145076<br>-683.510069<br>-75.716802	0.238382<br>0.125875<br>0.007701	-843.518530<br>-683.840999<br>-75.946985		
sum	-1602.371948	0.371958	-1603.306514	32.9	27.9

Cell culture

A549, MCF-7, HCT116, and MRC-5 cells are obtained from ATCC. A2780 and A2780cisR cells are kind gifts from Prof. Wee Han Ang (Department of Chemistry, National University of Singapore). A549cisR cells are cultured according to the previous report.[18] Human breast carcinoma MCF-7 cells, human lung carcinoma A549 cells, and cisplatin-resistant A549cisR cells were cultured in DMEM with 10% FBS and 100 μg mL^{-1} penicillin/streptomycin. Human ovarian carcinoma A2780 and cisplatin-resistant A2780cisR cells were cultured in RPMI-1640 with 10% FBS, 1% L-Glutamine, and 100 μg mL^{-1} penicillin/streptomycin. Human colon cancer HCT116 cells were cultured in McCoy's 5A medium with 10% FBS and 50 U mL^{-1} penicillin and streptomycin. Human lung fibroblast MRC-5 cells were cultured in MEM with 10% FBS, 1% NEAA, 1% L-Glutamine, 1% sodium pyruvate, and 100 μg mL^{-1} penicillin/streptomycin. For A2780cisR and A549cisR cells, 2 μM of cisplatin was added into the culture medium after the attachment to maintain the resistance. All the cells were cultured at 37 ºC in 5% CO_2.

Cytotoxicity

The cytotoxic profiles of complex against selected cell lines were obtained by both 3-(4,5-Dimethylthiazol-2-yl)-2,5-diphenyltetrazolium bromide (MTT) assay and sulforhodamine B (SRB) assay.[19] Cells were seeded into 96-well plates at a density of 2,500 cells per well (for MCF-7, A549, and A2780) or 5,000 cells per well (for A549cisR, A2780cisR, and MRC-5) and incubated for 24 h. Cells were then treated with medium containing various concentrations of

complexes for 6 h. The medium was replaced by phenol-red free medium and irradiated with white light (4 mW cm^{-2}) or kept in the dark. 30 min irradiation was applied for all biological experiments to guarantee the full activation of rhodaplatins in the cancer cells. Then, cells were further incubated in fresh medium for 42 h at 37 °C. For the MTT assay, after incubation, the culture medium was removed, cells were then incubated with FBS free medium containing 1 mg mL^{-1} MTT for 2 h. Medium containing MTT was removed and DMSO (150 µL) was added to each well. The absorbance was measured at 570 and 730 nm. For the SRB assay, after incubation, 100 µL cold trichloroacetic acid (TCA) solution (10% wt/vol, 4 ºC) was added to each well, and cells were incubated at 4 ºC for 1 h to fix cells. Then cells were washed with water four times, and 100 µL SRB solution (0.057% wt/vol) was added to each well. The plates were placed at room temperature for 0.5 h, and the unbound dye was removed by rinsing the plates with 1% (vol/vol) acetic acid four times. Next, 200 µL 10 mM Tris buffer (pH 10.5) was added to each well, and the plates were placed on a shaker to shake at 50 rpm for 10 min. The absorbance at 510 nm was recorded to represent the cell viability.

Annexin V/7-AAD double staining assay

A2780cisR cells (10^4 cells per well) were seeded in 6-well plates and cultured for 24 h. Cells were incubated with 10 µM rhodaplatin **2**, oxaliplatin, or oxaliplatin+rhodamine B (1:1 mole ratio) for 6 h. As a positive control, cells were treated with oxaliplatin (100 µM) for 6 h. The culture medium was replaced, and cells were washed with PBS twice and then irradiated with white light (4 mW cm^{-2}) or kept in the dark for 30 min. Next, cells were further incubated for 18 h. Cells were then trypsinized, collected by centrifugation (400 g, 5 min), and washed with annexin V-binding buffer (10 mM HEPES, 140 mM NaCl, 2.5 mM $CaCl_2$, pH 7.4). Samples containing 10^5 cells were stained with annexin-V (100 µg mL^{-1}, 5 µL) and 7-AAD (100 µg mL^{-1}, 1 µL) at room temperature for 15 min. A FACS Calibur flow cytometer was utilized for the analysis.

Cytotoxicity in tumor spheroids

A2780cisR cells (5,000 cells/well) were suspended in ultra-low attachment 6-well plates for 12 h with shaking at the speed of 12 rpm. Then the speed was increased to 50 rpm and cells were cultured for another 24 h. The cells were then cultured in regular conditions till the diameter reached 250 µm (in a total of 12 days). The medium was replaced every 3 days in the process of spheroid formation. Tumor spheroids were then transferred into ultra-low attachment 96-well plates (10 spheroids/well) and incubated with 100 µM complex for 6 h. The culture

medium containing drug was replaced with the drug-free fresh medium. The spheroids were irradiated with or without white light (4 mW cm^{-2}) for 0.5 h and then cultured in fresh medium for another 18 h. The tumor spheroids were finally transferred into confocal dishes, stained with 2 μM calcein AM and 10 ng mL^{-1} PI for 1 h, and visualized by confocal microscope (Leica SPE). Calcein AM was excited at 488 nm and recorded at 500-530 nm. PI was excited at 488 nm and recorded at 630-670 nm.

Cellular accumulation

A2780cisR cells (10^4 cells per well) were seeded in 6-well plates and cultured for 24 h before use. Cells were treated with 10 μM complex. After certain incubation time, cells were washed with ice-cold PBS for 3 times, harvested, and suspended in PBS buffer. The cell number was determined by a hemocytometer. Cells were then pelleted by centrifugation, then digested with 65% HNO_3 at 60 °C overnight. The Pt content was determined by ICP-MS (PE Nexion 2000).

Mitochondrial accumulation

Cells (5,000 cells per well) were seeded in confocal dishes and cultured for 24 h before use. Cells were treated with 10 μM rhodaplatin **2** for 6 h. Cells were then stained with 0.1 μM Mito tracker or ER tracker for 15 min. Fluorescent images were recorded on a laser confocal scanning microscope (Leica SP5). Pearson’s Colocalization Coefficient (PCC) was calculated using ImageJ.

The Pt amount in mitochondria was detected by ICP-MS. Cells (20,000 cells per well) were cultured in 6-well plates for 24 h. Then cells were treated with complex (10 μM) for 6 h. After washed with PBS twice, cells were collected, the cell number was counted by a hemocytometer, the mitochondria fraction was isolated by mitochondria isolation kit (Thermo Scientific). After digestion by 65% HNO_3 at 60 °C overnight, the Pt amount was detected by ICP-MS. The Pt amount in the mitochondria and rest fraction were combined as the whole-cell uptake, as no fraction was discarded during the isolation process.

Platinum accumulation in mitochondrial and nuclear DNA

A2780cisR cells were seeded in 10-cm dishes and allowed to grow to 80% confluence. Cells were treated with complex (10 μM) for 6 h. Medium containing drugs was then replaced by drug-free medium and cells were irradiated with white light or kept in the dark for 30 min. Cells were further incubated with fresh medium for 1 h. Cells were washed 3 times with PBS and

collected by centrifugation. Cellular fractionation of cells was isolated using Mitochondrial Isolation Kit (Thermo Scientific) following the instruction. After lysis, nuclei were isolated by centrifugation at 700 g for 10 min. Mitochondria were isolated by centrifugation at 3000 g for 15 min. Nuclear DNA and mitochondrial DNA were purified by GenElute Mammalian Genomic DNA Miniprep kit. DNA content was determined by a NanoDrop OneC Spectrophotometer. Pt content on DNA was determined by ICP-MS after digestion with 65% HNO_3 at 60 ºC overnight. Statistically significant differences (ns not significant, * $p < 0.05$, ** $p < 0.01$, *** $p < 0.001$) were calculated using two two-tailed, unpaired student's t-test.

Mitochondrial and nuclear DNA damage

A2780cisR cells (10,000 cells per well) were seeded in 6-well plates and treated with 10 μM complex for 6 h. Medium containing drugs was then replaced by drug-free medium and cells were irradiated with white light (4 mW cm^{-2}) or kept in the dark for 30 min. Cells were further incubated with fresh medium for 1, 6, or 12 h. Cells were washed 3 times with PBS and collected by centrifugation. DNA was purified using a GenElute Mammalian Genomic DNA Miniprep kit. Mitochondrial and genomic DNA damage was analyzed by PCR following the previous protocol.[20] 50 ng of DNA was mixed with dNTP (0.2 mM each), primers (0.5 μM each), and Platinum SuperFi DNA polymerase (0.02 U μL^{-1}).

Primers chosen for the experiment:

12.2 kb region of the DNA polymerase gene β: sense 5'-CAT GTC ACC ACT GGA CTC TGA AC-3' and antisense 5'-CCT GGA GTA GGA ACA AA ATT GCT-3'

8.9 kb mitochondria fragment: sense 5'-TCT AAG CCT CCT TAT TCG AGC CGA-3' and antisense 5'-TTT CAT CAT GCG GAG ATG TTG GAT GG-3'

Cycling conditions were set as:

For nuclear DNA: 95 °C (2 min); 95 °C (10 s), 64 °C (10 s), 68 °C (6 min), 30 cycles; 68 °C (5 min)

For mitochondrial DNA: 95 °C (2 min); 95 °C (10 s), 67 °C (10 s), 68 °C (4.5 min), 30 cycles; 68 °C (5 min)

The quantitation of the PCR product was performed by Quant-iT PicoGreen dsDNA assay following the instruction (Thermofisher Scientific).

JC-1 staining

A2780cisR cells (10,000 cells per well) were seeded in 2 cm confocal dishes for 24 h. Then cells were treated with 10 μM rhodaplatin **2**, rhodamine B, oxaliplatin, or oxaliplatin +

rhodamine B (1:1 mol ratio) for 6 h. Medium containing drugs was then replaced by drug-free medium and cells were irradiated with white light (4 mW cm^{-2}) or kept in the dark for 30 min. Cells were further incubated with fresh medium for 18 h. Cells were then stained with 10 µg mL^{-1} JC-1 in the medium for 30 min. For the positive control group, cells were pre-treated with H_2O_2 (250 µM) for 6 h, then stained with JC-1.[21] Cells were then washed with PBS buffer for 3 times and the images were taken by Laser Confocal Scanning Microscope (Leica SP5). The dye was excited at 488 nm, the fluorescence of the aggregate form and monomer form was recorded at 590-610 (red) and 520-540 nm (green), respectively.

Hoechst staining

A2780cisR cells were seeded into 6-well plates and treated with 10 µM rhodaplatin **2**, rhodamine B, oxaliplatin, or oxaliplatin+rhodamine B (1:1 mol ratio) for 6 h. Medium containing drugs was then replaced by drug-free medium and cells were irradiated with white light (4 mW cm^{-2}) or kept in the dark for 30 min. Cells were further incubated with fresh medium for 18 h. Cells were then washed with PBS buffer for 3 times and incubated with PBS containing 5 µg mL^{-1} Hoechst for 20 min. Cells were washed with PBS for 3 times and images were recorded using Laser Confocal Scanning Microscope (Leica SPE) (Ex 405 nm, Em 460-480 nm).

For the cells treated with Z-VAD-FMK (caspase inhibitor), cells were pretreated with Z-VAD-FMK (10 µM) for 30 min, then cells were treated with complexes as the above-mentioned process.

Translocation of AIF and endoG

A2780cisR cells (5,000 cells per well) were plated in confocal dishes, treated with 10 µM complex for 6 h, irradiated for 0.5 h with white light (4 mW cm^{-2}), and further incubated for 12 h. Cells were fixed with 10% formalin at 37 °C for 5 min and washed with PBS containing 3% FBS for 3 times. Cells were permeabilized with PBS containing 0.1% Triton X 100 and 2% FBS at 4 °C overnight. Cells were stained with the 1st antibody against AIF (Abcam, 1:1000) or endonuclease G (Abcam, 1:1000) in PBS with 2% FBS at 4 °C overnight and washed 3 times with PBS. Cells were then stained with Alexa 488 labeled secondary antibody (Abcam, 1:2000) in PBS with 2% FBS at 37 °C for 1 h and washed 3 times with PBS. Cells were finally stained with 1 µg mL^{-1} Propidium iodide (PI) for 10 min. Cells were visualized by confocal microscopy (Leica SP5). PI was excited at 488 nm and recorded at 630-670 nm. The antibody was excited at 488 nm and recorded at 510-530 nm.

Caspase activation test

The activation of caspase-3/7 was analyzed by CellEvent Caspase-3/7 Green Detection Reagent. A2780cisR cells (3,000 cells per well) were seeded in 48-well plates and allowed to attach for 24 h. Cells were treated with 10 μM rhodaplatin **2** for 6 h. Medium containing drugs was then replaced by drug-free medium, and cells were irradiated with white light (4 mW cm^{-2}) for 30 min. Cells were further incubated with fresh medium for 0, 2, 4, 12, 18 h. Cells treated with 50 μM of cisplatin for 24 h were introduced as a positive control. After the treatment, 2 μM of detection reagent in PBS with 5% FBS was added into cells and incubated for 0.5 h. Cells were fixed with 10% Formalin for 15 min. Cells were finally visualized at a laser confocal scanning microscope (Leica SPE). The fluorescence was excited at 488 nm and recorded at 520-540 nm. For the cells treated with Z-VAD-FMK (caspase inhibitor), cells were pretreated with Z-VAD-FMK (10 μM) for 30 min, then complexes were added into the pretreated cells as the above-mentioned process.

Western blot

A2780cisR cells (10,000 cells per well) were cultured in 6-wells plates for 24 h. Then cells were treated with complex for 6 h. Medium containing drugs was then replaced by drug-free medium, and cells were irradiated with white light (4 mW cm^{-2}) for 30 min. Cells were further incubated with fresh medium for 0, 2, 12, 18 h. Cells were washed 3 times with ice-cold PBS. For whole-cell lysate, cells were directly scraped into Laemmli buffer (0.05 M Tris-Cl, 0.1 M dithiothreitol, 10% glycerol, 2% SDS, 0.05% bromophenol blue, pH 6.8) and denatured at 95 °C for 10 min. For mitochondrial and cytosol fraction, cells were scraped into PBS buffer and collected by centrifugation. The mitochondria and cytoplasm were then isolated using Thermo Scientific Mitochondrial Isolation Kit for Cultured Cells following the instruction. Then the separated proteins from mitochondria or cytoplasm were mixed with Laemmli buffer (0.05 M Tris-Cl, 0.1 M dithiothreitol, 10% glycerol, 2% SDS, 0.05% bromophenol blue, pH 6.8) and denatured at 95 °C for 10 min. The protein samples were separated on SDS-PAGE gel and transferred to PVDF membrane (Millipore). The membrane was incubated with γ-H2AX (Ser139) (Cell Signaling Technology, 1:1000), cytochrome c (Cell Signaling Technology, 1:1000), and β-Actin (Abcam, 1:1000) primary antibodies and then a secondary antibody (rabbit-anti-mouse/goat-anti-rabbit, Novex). ECL mixture (Bio-Rad) was then applied to the membrane. Protein bands were visualized in a Bio-Rad ChemiDoc Touch System.

Statistical analysis

All the results were presented as Mean ± SD where applicable. GraphPad Prism 6 was used for statistical analysis.

Table S1. The reduction potential, TOF value of RhB towards the complexes, and conversion rate of **2a**-**3c** and rhodaplatins.

Complex	Reduction potential (V)[a)]	TOF value [min^{-1}][b)]	Conversion rate [$\mu M\ min^{-1}$]
**2a**	-0.84	0	0
**2b**	-0.67	1.3×10^{-5}	1.3×10^{-3}
**2c**	-0.48	3.0×10^{-5}	3.0×10^{-3}
**3a**	-0.81	1.0×10^{-5}	1.0×10^{-3}
**3b**	-0.68	2.7×10^{-5}	2.7×10^{-3}
**3c**	-0.56	2.0×10^{-4}	2.0×10^{-2}
Rhodaplatin **1**	-0.36	-	20
Rhodaplatin **2**	-0.34	-	48

[a)] The reduction potential of the complex was detected in H_2O. [b)] The photocatalysis of RhB towards Pt(IV) complexes was carried out in PBS buffer (pH 7.4) containing 2 mM sodium ascorbate.

Table S2. The fluorescence lifetime and quantum yield of rhodaplatins and rhodamine B in PBS buffer (pH 7.4).

	Rhodamine B	Rhodaplatin **1**	Rhodaplatin **2**
Quantum yield	0.34	0.18	0.19
fluorescence lifetime [ns]	2.0	1.0	1.1

Table S3. The cytotoxicity of different complexes against various cancer cell lines. Cells were treated with complex for 6 h, then the culture medium was replaced with drug-free fresh medium, and cells were irradiated with or without visible light (400-760 nm, 4 mW cm^{-2}) for 30 min. Then cells were cultured for another 42 h. The IC_{50} value was determined by the SRB assay.

Cell line	IC_{50} [μM]				PI[c)]	FI[d)]	IC_{50} [μM]			PI[c)]	FI[d)]
	Carboplatin	RhB	RhB+**2c**[b)]	Rhodaplatin **1**			Oxaliplatin	RhB+**3c**[b)]	Rhodaplatin **2**		
A2780	342 ± 24	238 ± 20	227 ± 16	231 ± 12	6.4	8.9	64 ±5	252 ± 10	101 ± 7	3.6	2.4
	319 ± 26	214 ± 16	202 ± 18	36 ± 4			68 ± 7	227 ± 13	28 ± 4		
A2780 cisR (RF)[a)]	> 400	273 ± 22	263 ± 24	271 ± 20	7.1	> 10.5	203 ± 14	287 ± 15	136 ± 13	6.5	9.3
	> 400 (-)	229 ± 14 (1.1)	229 ± 17 (1.1)	38 ± 6 (1.0)			196 ± 15 (2.9)	247 ± 16 (1.1)	21 ± 6 (0.8)		
A549	> 400	301 ± 26	279 ± 15	260 ± 18	5.0	> 7.7	89 ± 14	271 ± 18	109 ± 9	3.5	2.7
	> 400	267 ± 21	231 ± 12	52 ± 5			85 ± 8	223 ± 13	31 ± 4		
A549cisR (RF)[a)]	> 400	332 ± 19	304 ± 25	297 ± 21	5.2	> 7.0	231 ± 14	294 ± 12	155 ± 12	5.2	7.9
	> 400 (-)	286 ± 18 (1.1)	266 ± 15 (1.2)	57 ± 7 (1.1)			238 ± 17 (2.8)	231 ± 11 (1.0)	30 ± 5 (1.0)		

In the dark With irradiation

[a)] Resistance Factor (RF): The IC_{50} value in A2780cisR (A549cisR) cells under irradiation / IC_{50} value in A2780 (A549) cells under irradiation; [b)] The IC_{50} values of free complex **2c** or **3c** are > 200 μM in all the tested cell; [c)] Phototoxic Index (PI): The IC_{50} of the dark group treated with rhodaplatin / The IC_{50} of the irradiation group treated with rhodaplatin; [d)] Fold Increase (FI): The IC_{50} of carboplatin (or oxaliplatin) of irradiation group / The IC_{50} of rhodaplatin **1** (or rhodaplatin **2**) of irradiation group.

Table S4. The cytotoxicity of complex **4** in various cancer cell lines. Cells were treated with complex for 6 h, then the culture medium was replaced with drug-free fresh medium. Cells were cultured for another 42 h. The IC_{50} value was calculated by the MTT assay.

Cell line	IC_{50} [μM]
A2780	170 ± 15
A2780cisR	317 ± 41
MCF-7	> 400

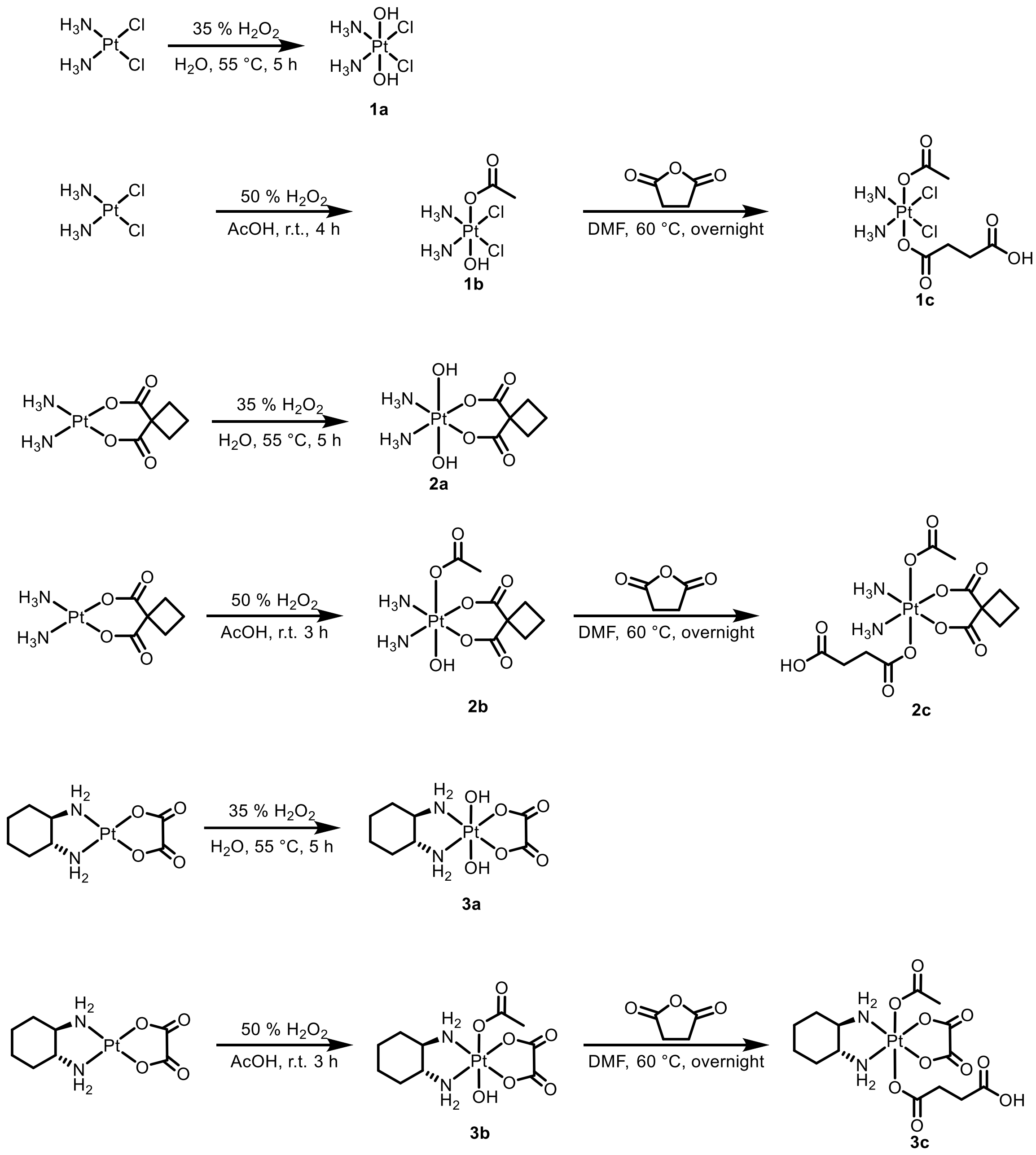

Scheme S1. The synthetic route for complexes **1a**-**3c**.

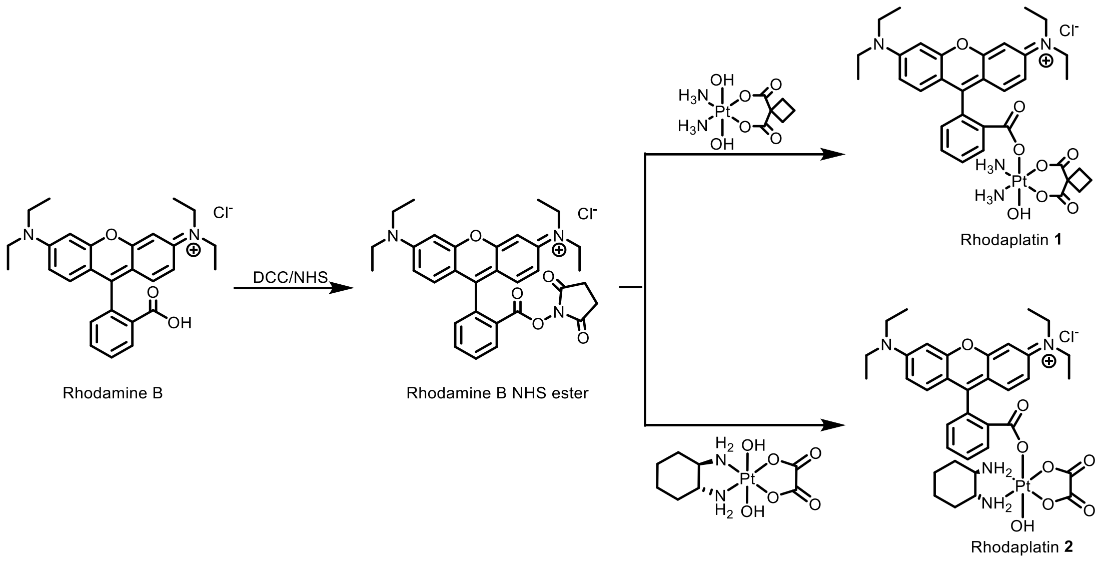

Scheme S2. The synthetic route for rhodaplatins.

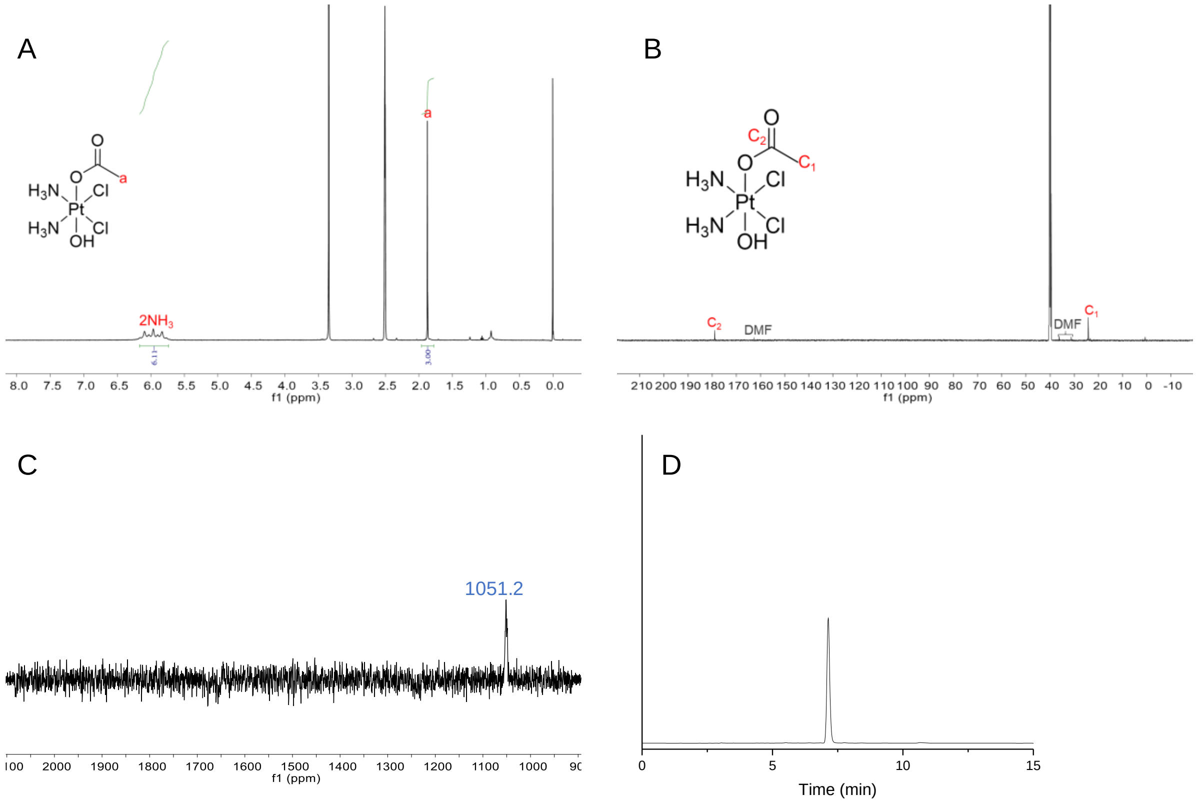

Figure S1. The (A) ^{1}H NMR, (B) ^{13}C NMR, (C)^{195}Pt NMR, and (D) HPLC of **1b**. NMR samples were dissolved in DMSO-d_6.

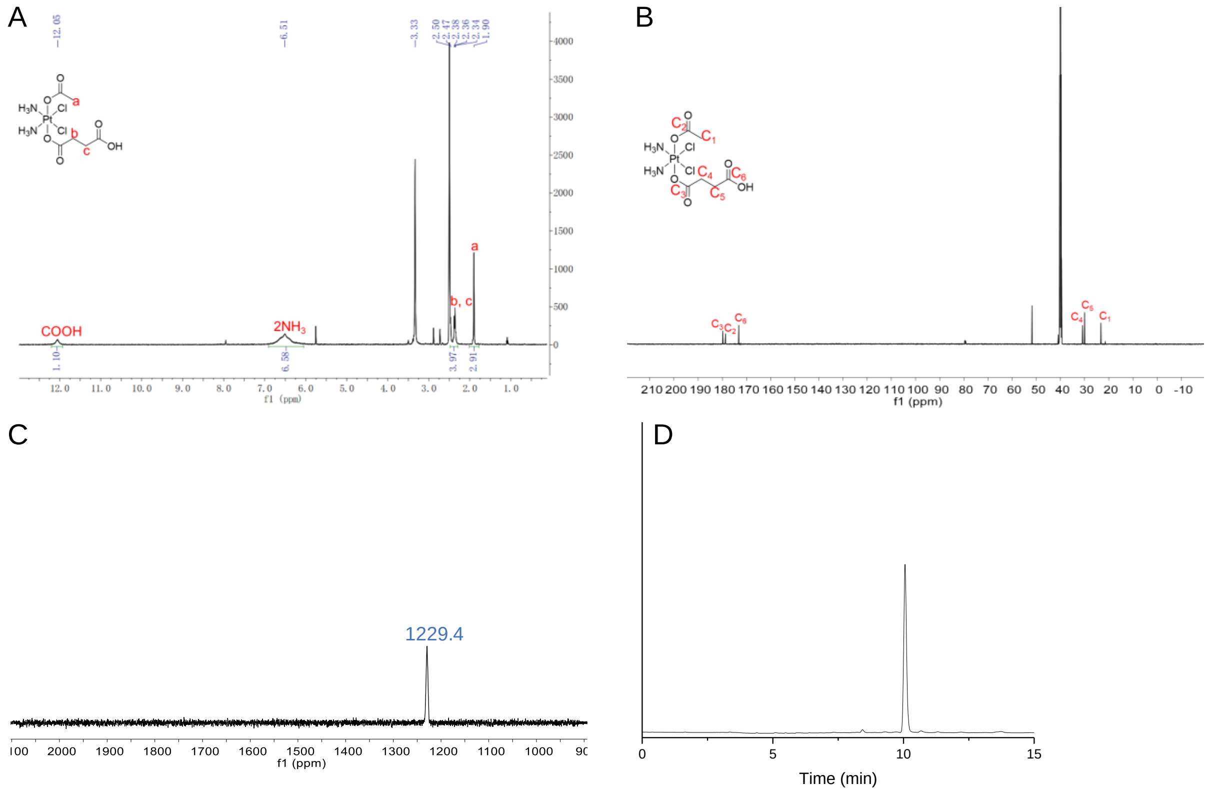

Figure S2. The (A) ^{1}H NMR, (B) ^{13}C NMR, (C)^{195}Pt NMR, and (D) HPLC of **1c**. NMR samples were dissolved in DMSO-d_6.

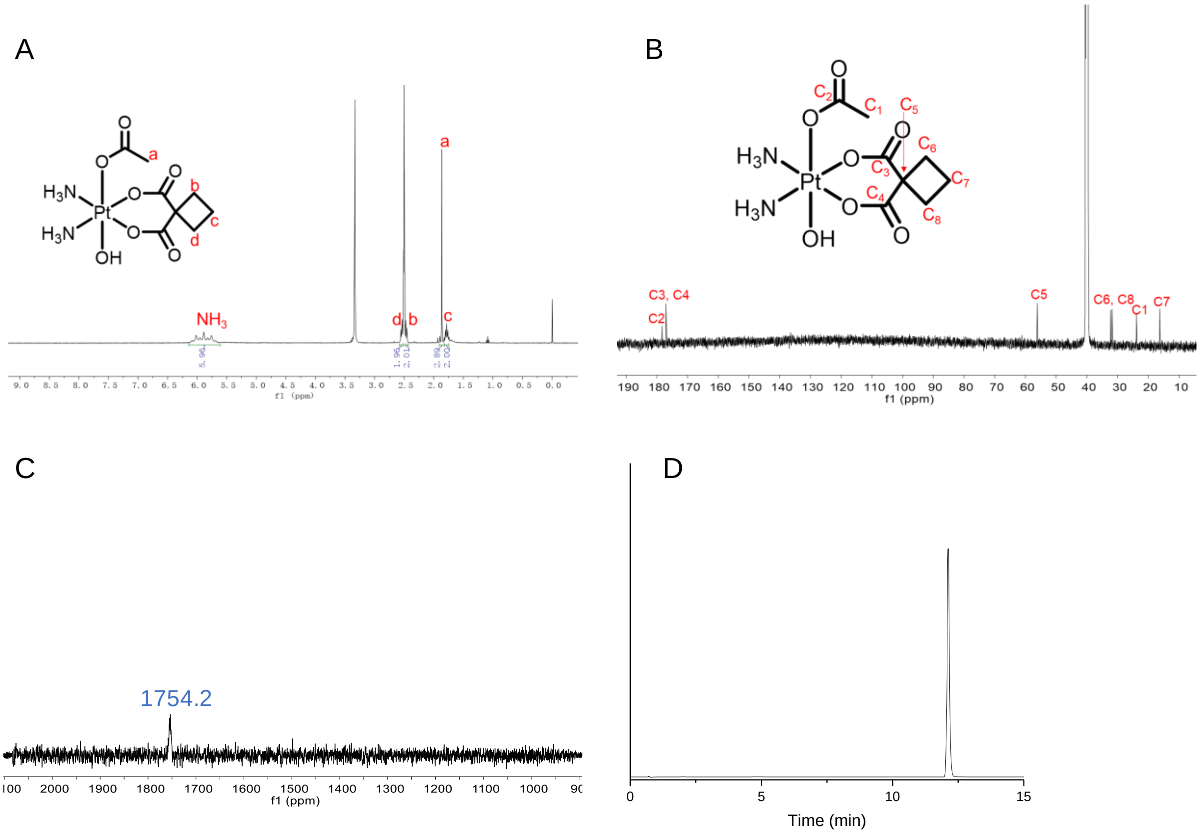

Figure S3. The (A) ^{1}H NMR, (B) ^{13}C NMR, (C) ^{195}Pt NMR, and (D) HPLC of **2b**. NMR samples were dissolved in DMSO-d_6.

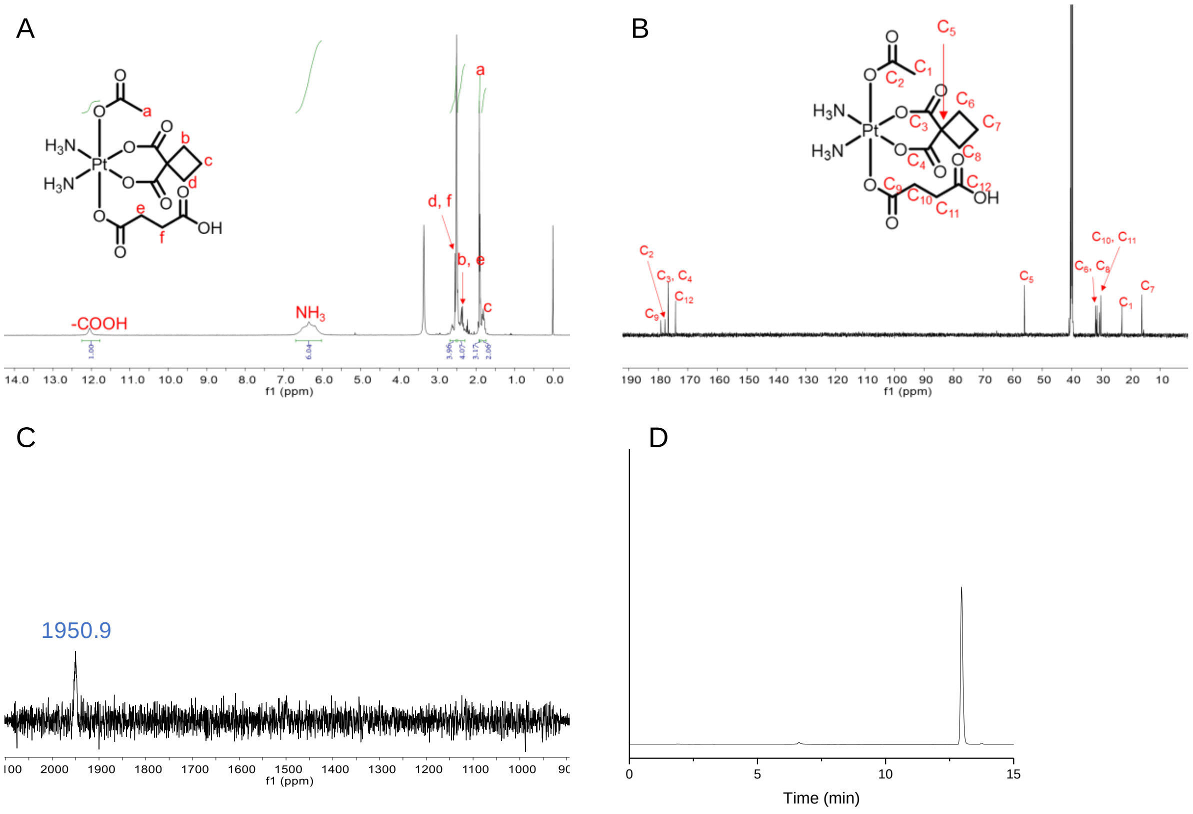

Figure S4. The (A) ^{1}H NMR, (B) ^{13}C NMR, (C)^{195}Pt NMR, and (D) HPLC of **2c**. NMR samples were dissolved in DMSO-d_6.

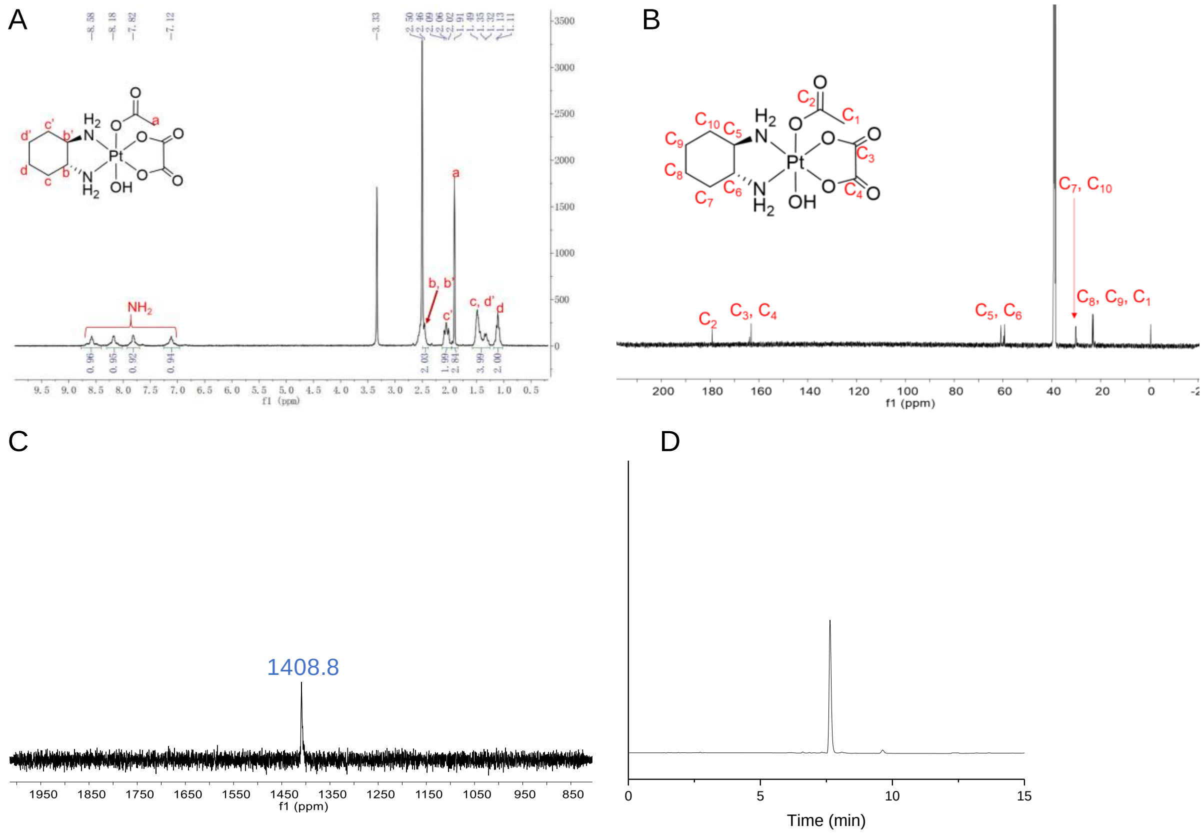

Figure S5. The (A) ^{1}H NMR, (B) ^{13}C NMR, (C)^{195}Pt NMR, and (D) HPLC of **3b**. NMR samples were dissolved in DMSO-d_6.

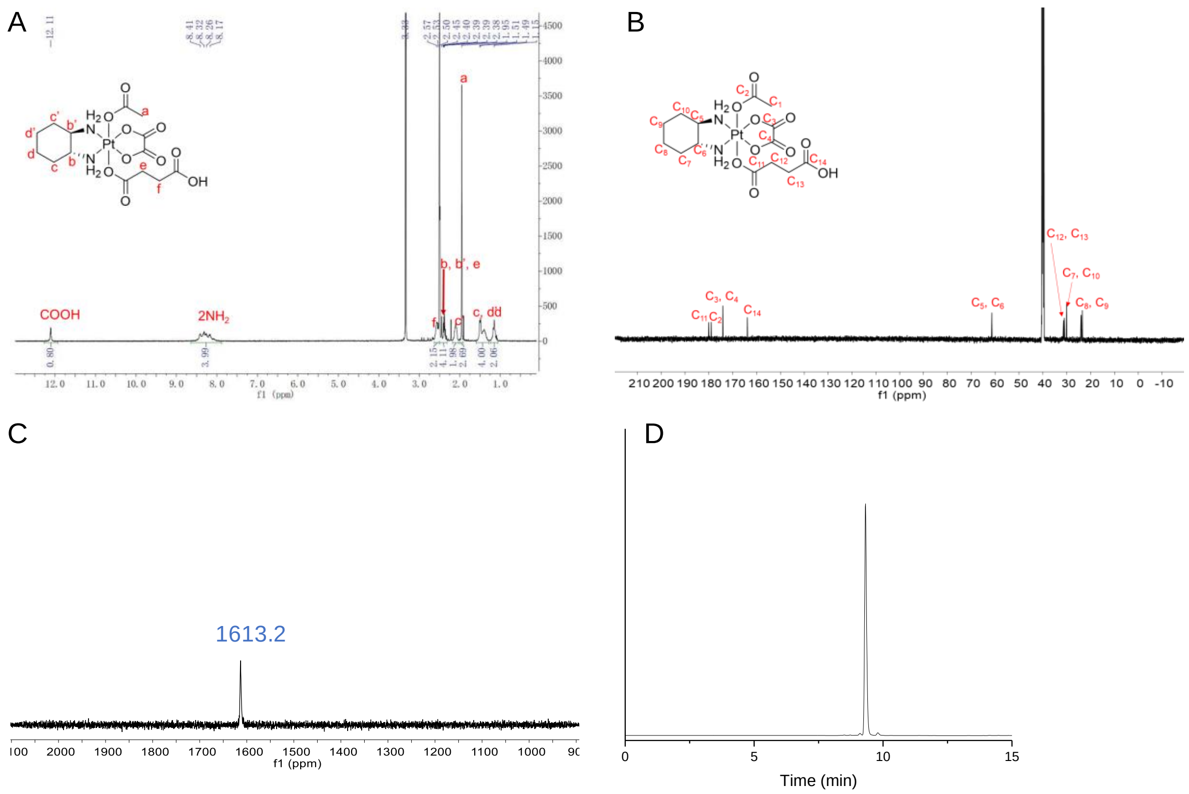

Figure S6. The (A) ^{1}H NMR, (B) ^{13}C NMR, (C)^{195}Pt NMR, and (D) HPLC of **3c**. NMR samples were dissolved in DMSO-d_6.

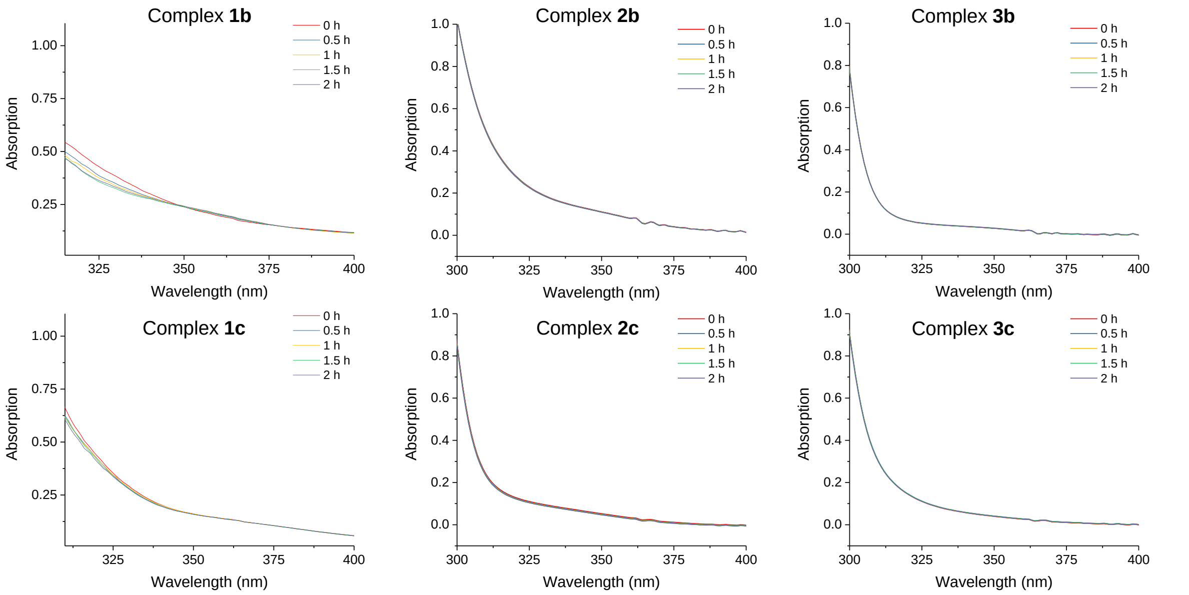

Figure S7. The stability of different complexes in PBS buffer (pH 7.4) containing 2 mM sodium ascorbate at 37 ºC. UV-Vis spectra of each solution were recorded at different time points to represent the stability of complexes.

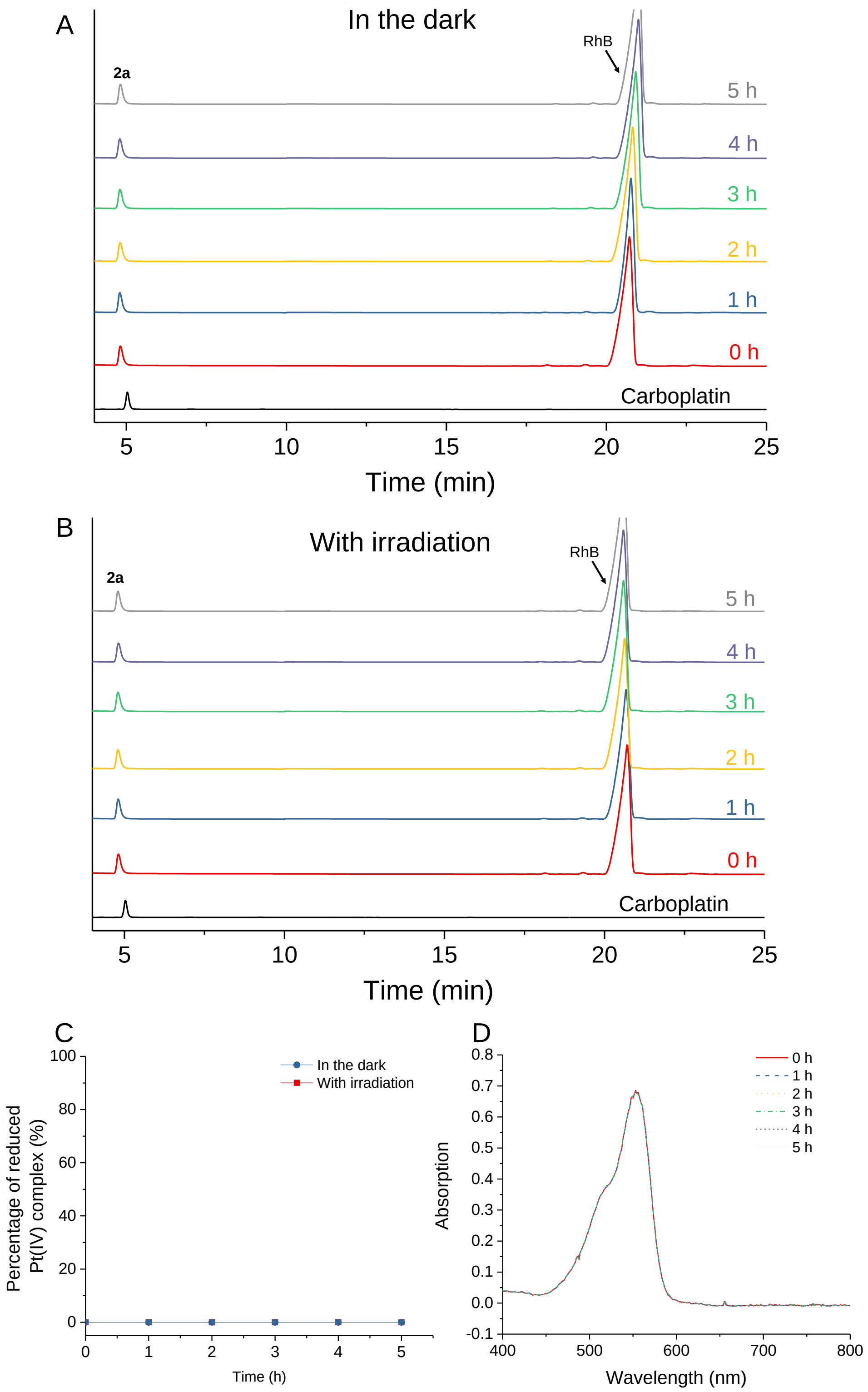

Figure S8. The HPLC chromatography of complex **2a** (100 µM) in the PBS buffer (pH 7.4) containing 2 mM sodium ascorbate and 100 µM RhB (A) kept in the dark or (B) upon irradiation (400-760 nm, 4 mW cm^{-2}) for 5 h. Eluent program: 0-8 min, 10% B; 8-12 min, 10% B-100% B; 12-15 min, 100% B; 15-20 min, 100% B- 0% B. Solution A is H_2O + 0.1% formic acid. Solution B is acetonitrile + 0.1% formic acid. (C) The percentage of reduced complex **2a** in figures S8A and S8B. (D) The UV-Vis spectra of the whole mixture in figure S8B at different time points.

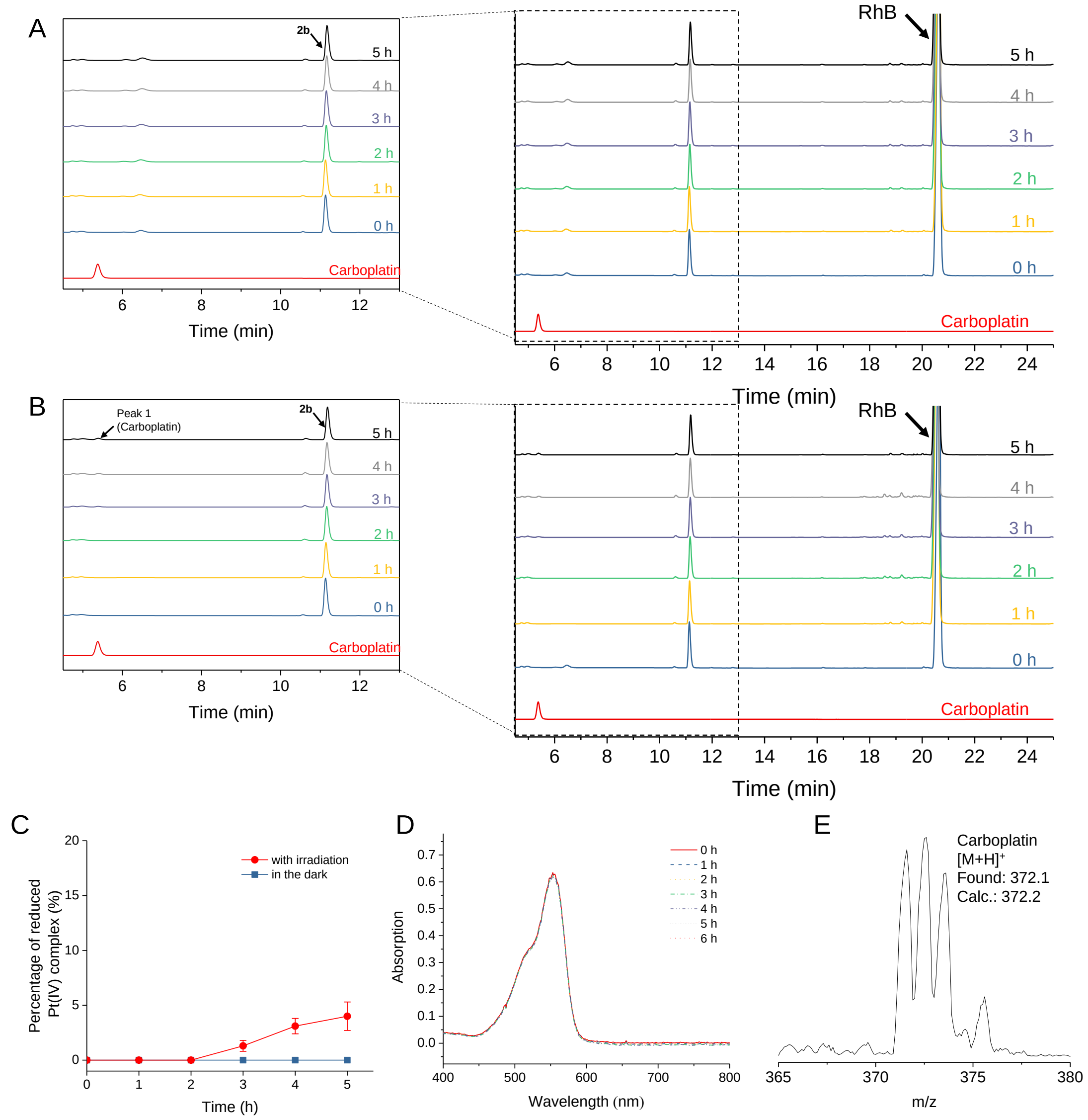

Figure S9. The HPLC chromatography of complex **2b** (100 μM) in the PBS buffer (pH 7.4) containing 2 mM sodium ascorbate and 100 μM RhB (A) kept in the dark or (B) upon irradiation (400-760 nm, 4 mW cm^{-2}) for 5 h. Eluent program: 0-8 min, 10% B; 8-12 min, 10% B-100% B; 12-15 min, 100% B; 15-20 min, 100% B- 0% B. Solution A is H_2O + 0.1% formic acid. Solution B is acetonitrile + 0.1% formic acid. (C) The percentage of reduced complex **2b** in figures S9A and S9B. (D) The UV-Vis spectra of the whole mixture in figure S9B at different time points. (E) The ESI-MS spectra of peak 1 in figure S9B, indicating that carboplatin is generated.

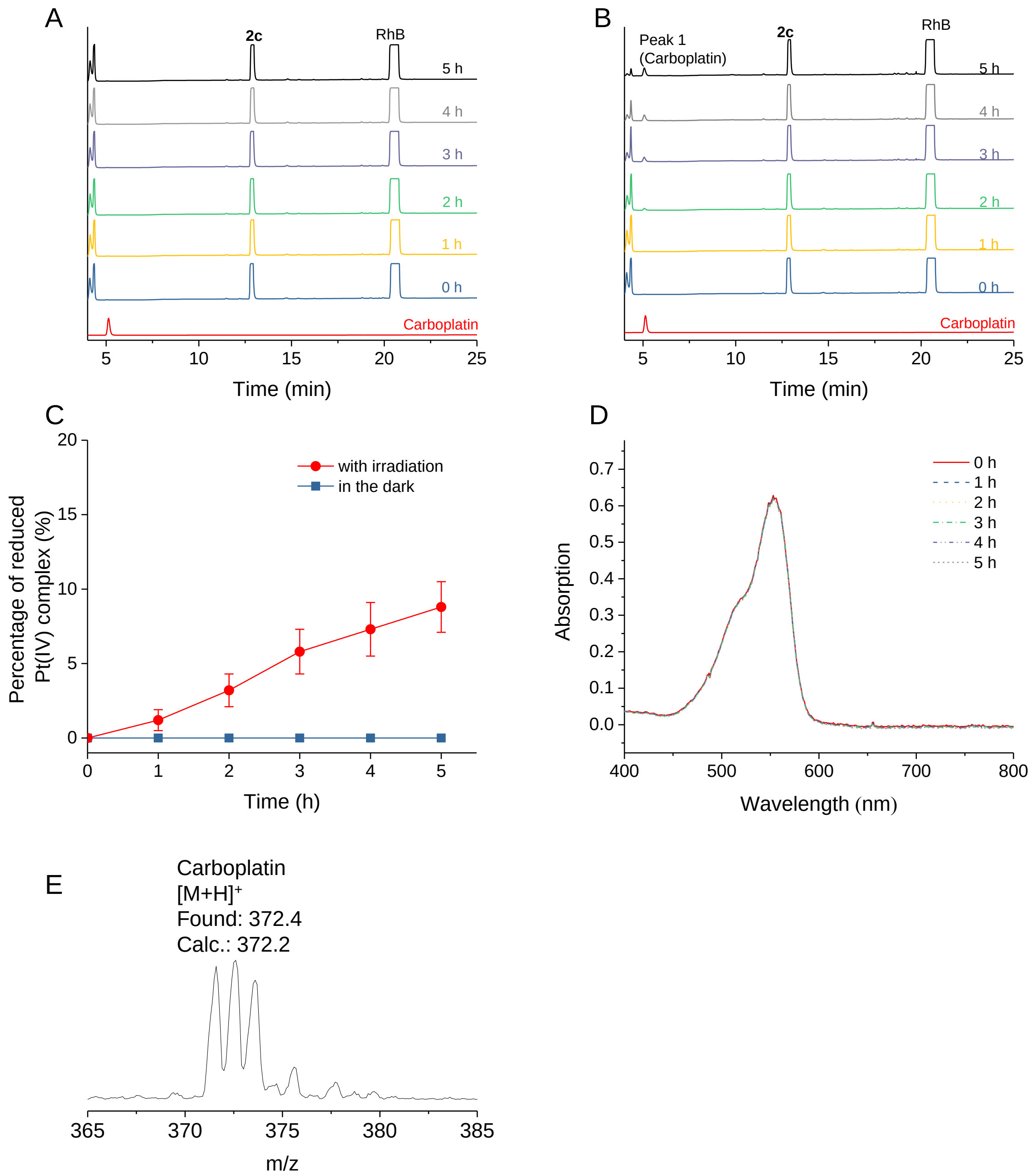

Figure S10. The HPLC chromatography of complex **2c** (100 μM) in the PBS buffer (pH 7.4) containing 2 mM sodium ascorbate and 100 μM RhB (A) kept in the dark or (B) upon irradiation (400-760 nm, 4 mW cm^{-2}) for 5 h. Eluent program: 0-8 min, 10% B; 8-12 min, 10% B-100% B; 12-15 min, 100% B; 15-20 min, 100% B- 0% B. Solution A is H_2O + 0.1% formic acid. Solution B is acetonitrile + 0.1% formic acid. (C) The percentage of reduced complex **2c** in figures S10A and S10B. (D) The UV-Vis spectra of the whole mixture in figure S10B at different time points. (E) The ESI-MS spectra of peak 1 in figure S10B, indicating that carboplatin is generated.

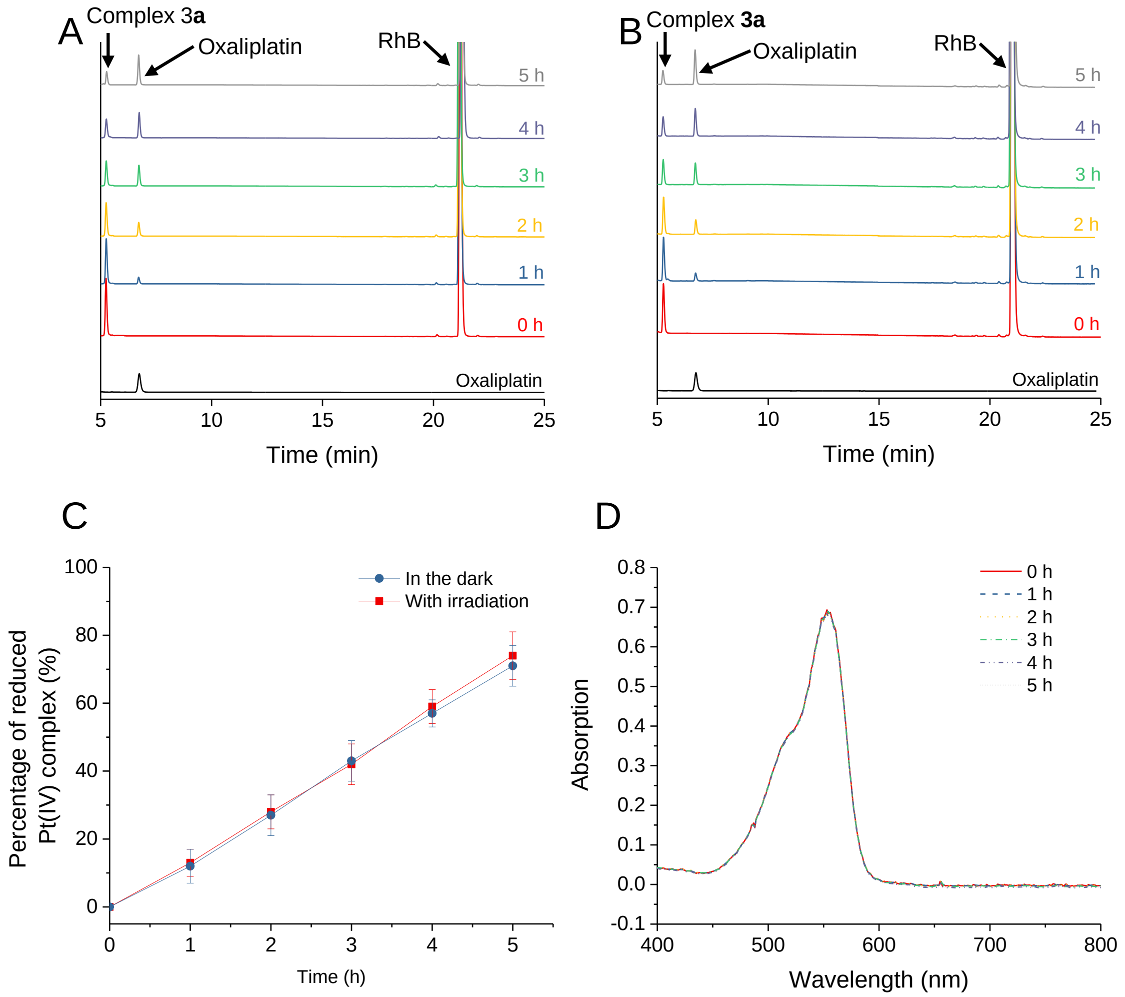

Figure S11. The photocatalysis ability of rhodamine B (100 µM) towards complex **3a** (100 µM) in PBS buffer (pH 7.4) containing 2 mM sodium ascorbate. The mixed solution was (A) kept in the dark or (B) irradiated with visible light (400-760 nm, 4 mW cm^{-2}) for 5 h. At each time point, the solution was analyzed by HPLC. Eluent program: 0-8 min, 4% B; 8-12 min, 4% B-100% B; 12-15 min, 100% B; 15-20 min, 100% B- 0% B. Solution A is H_2O + 0.1% formic acid. Solution B is acetonitrile + 0.1% formic acid. (C) The percentage of reduced Pt(IV) in figures S11A and S11B. (D) The UV-Vis spectra of the whole mixture from figure S11B, no change of absorption intensity of rhodamine B was detected, indicated that rhodamine B remained intact.

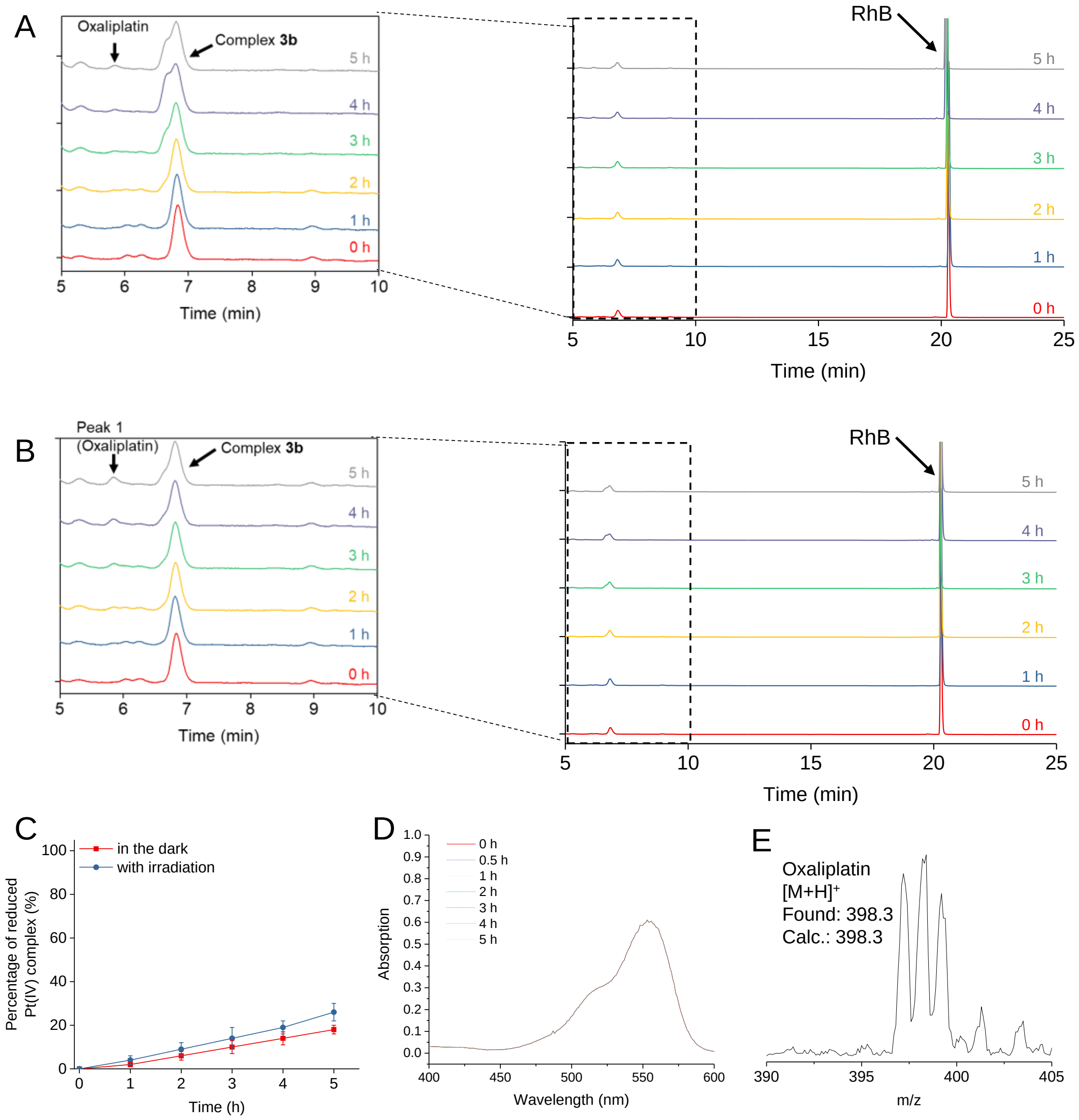

Figure S12. The photocatalysis ability of rhodamine B (100 μM) towards complex **3b** (100 μM) in PBS buffer (pH 7.4) containing 2 mM sodium ascorbate. The mixed solution was (A) kept in the dark or (B) irradiated with visible light (400-760 nm, 4 mW cm^{-2}) for 5 h. At each time point, the solution was analyzed by HPLC. Eluent program: 0-8 min, 4% B; 8-12 min, 4% B-100% B; 12-15 min, 100% B; 15-20 min, 100% B- 0% B. Solution A is H_2O + 0.1% formic acid. Solution B is acetonitrile + 0.1% formic acid. (C) The percentage of reduced Pt(IV) in figures S12A and S12B. (D) The UV-Vis spectra of the whole mixture from figure S12B, no change of absorption intensity of rhodamine B was detected, indicated the rhodamine B remained intact. (E) The ESI-MS spectra of peak 1 in figure S12B, indicating that oxaliplatin is formed.

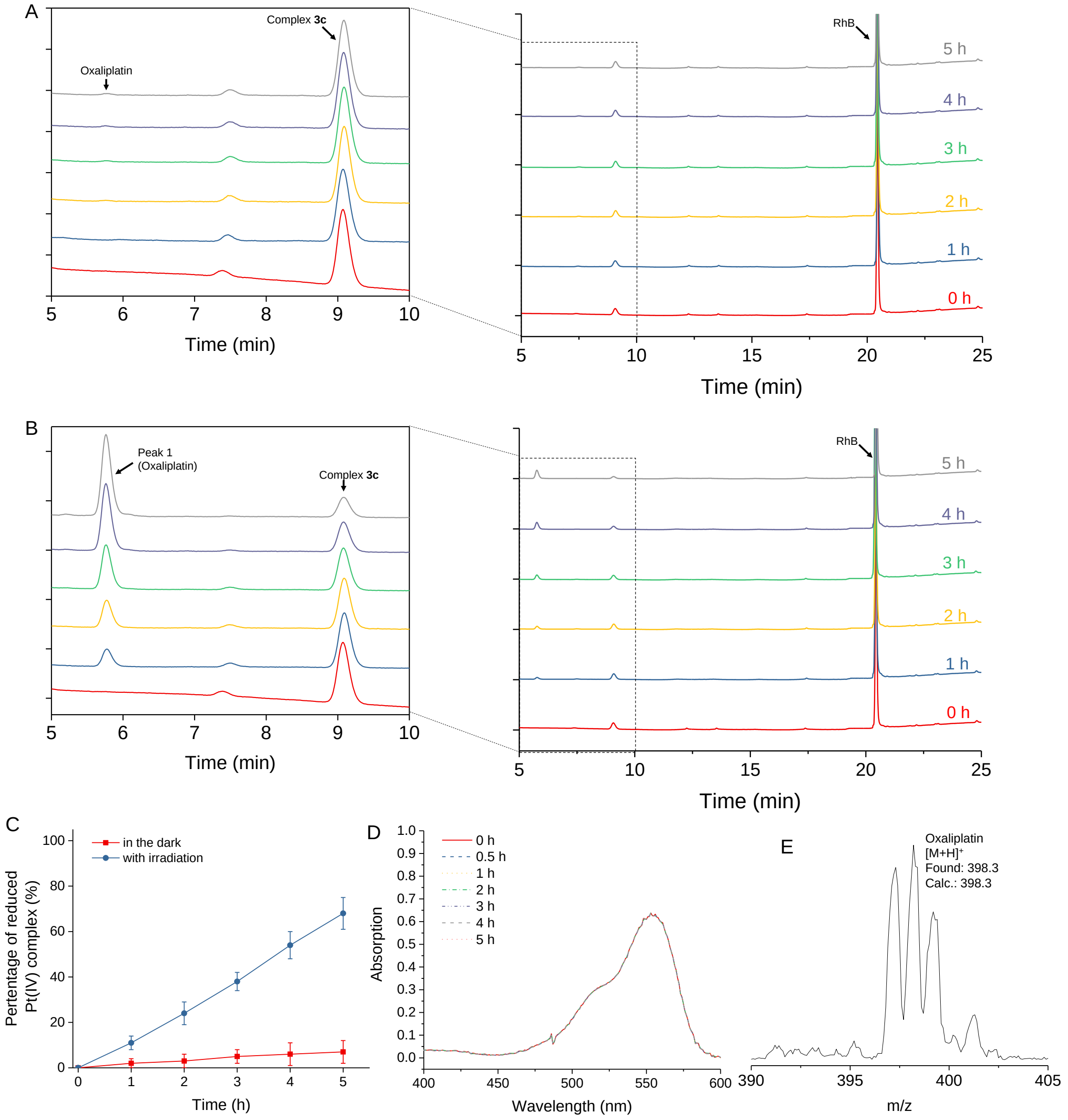

Figure S13. The photocatalysis ability of rhodamine B (100 μM) towards complex **3c** (100 μM) in PBS buffer (pH 7.4) containing 2 mM sodium ascorbate. The mixed solution was (A) kept in the dark or (B) irradiated with visible light (400-760 nm, 4 mW cm^{-2}) for 5 h. At each time point, the solution was analyzed by HPLC. Eluent program: 0-8 min, 10% B; 8-12 min, 10% B-100% B; 12-15 min, 100% B; 15-20 min, 100% B- 0% B. Solution A is H_2O + 0.1% formic acid. Solution B is acetonitrile + 0.1% formic acid. (C) The percentage of reduced Pt(IV) in figures S13A and S13B. (D) The UV-Vis spectra of the whole mixture from figure S13B, no change of absorption intensity of rhodamine B was detected, indicated the rhodamine B remained intact. (E) The ESI-MS spectra of peak 1 in figure S13B, indicating that oxaliplatin is formed.

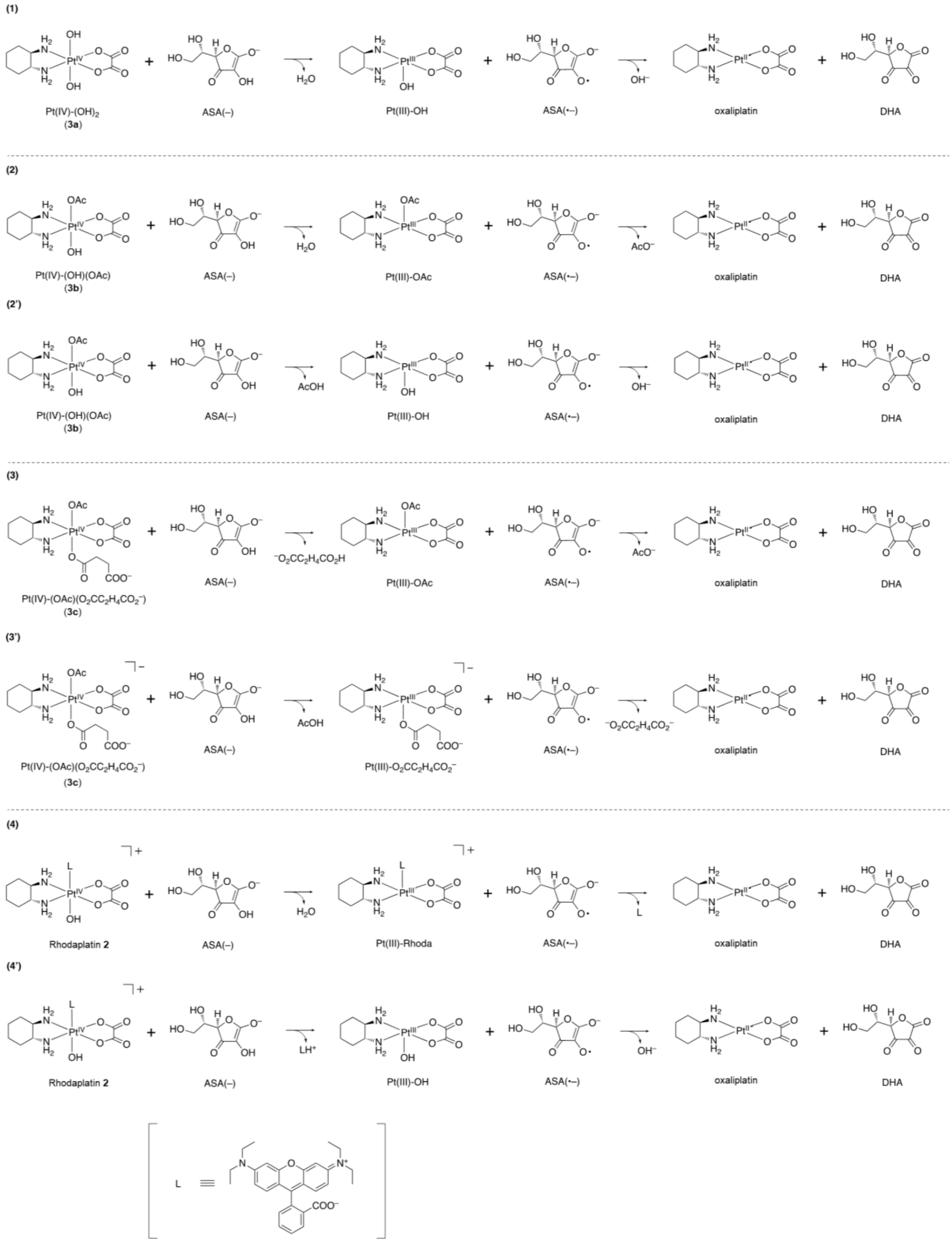

Figure S14. Reactions examined by DFT calculations.

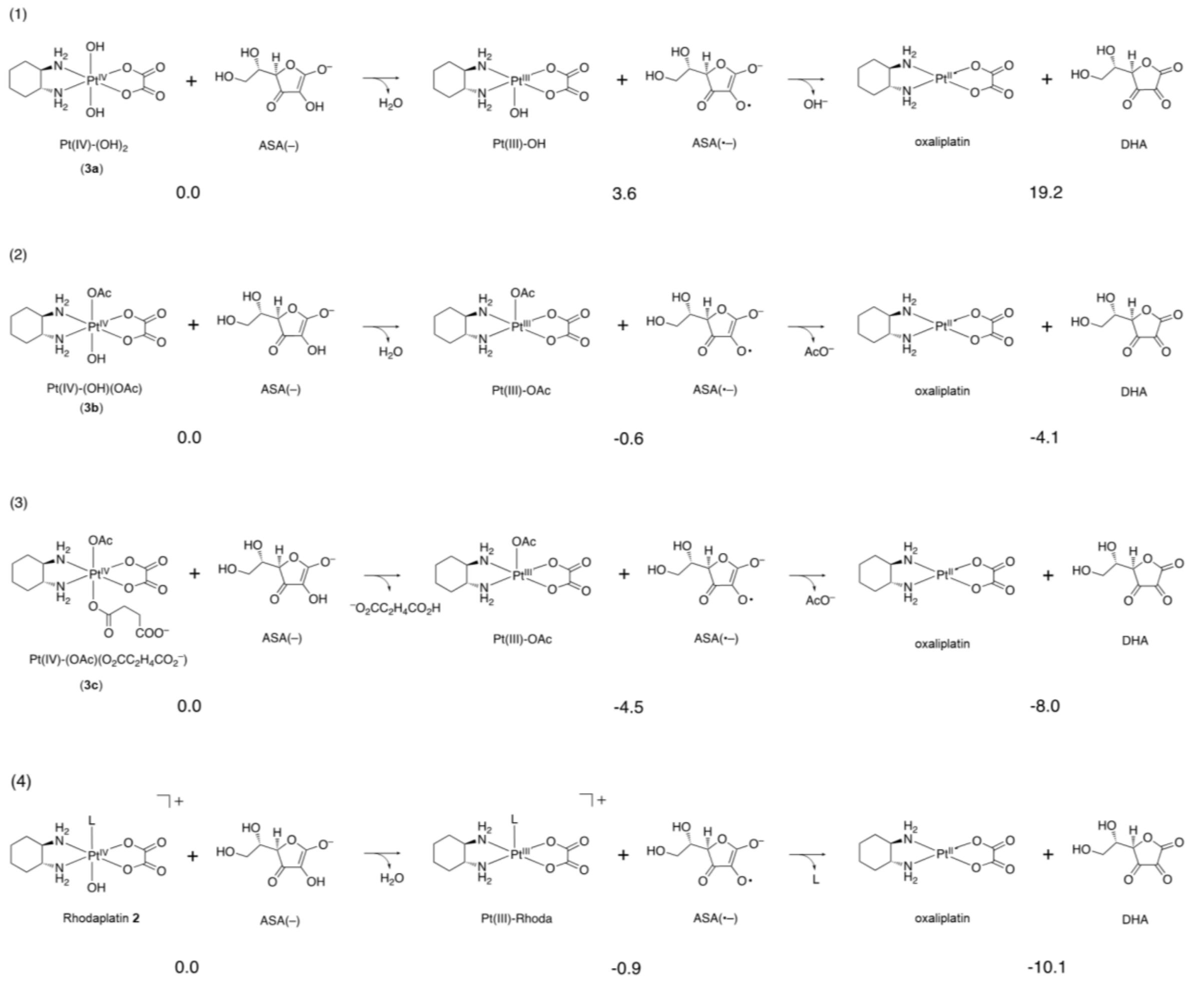

Figure S15. DFT-calculated relative energies (Δ(E2+ZPE)) in kcal mol^{-1}. The calculation result suggested that Pt(IV) complexes with lower reduction potential (i.e., more negative Ep value) require more energy to form Pt(III) intermediates, which makes them harder to reduce.

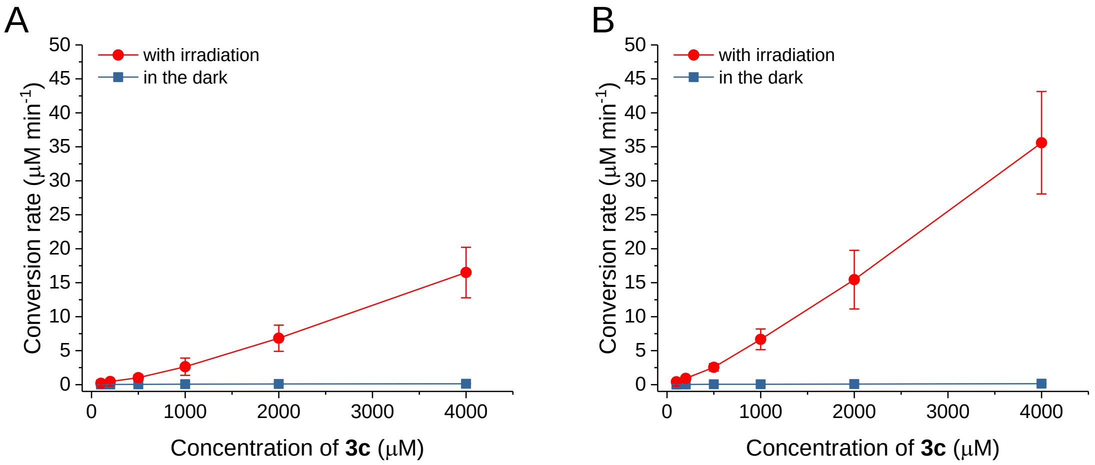

Figure S16. The rate of RhB-catalyzed photoreduction of complex **3c** with different concentrations of substrate (complex **3c**) upon (A) low-dose (4 mW cm^{-2}) or (B) high-dose (20 mW cm^{-2}) irradiation. In both experiments, the concentrations of RhB and sodium ascorbate are 100 μM and 2 mM, respectively. When the reaction rate reached the highest value, the concentration ratio of RhB to **3c** is 1:40, indicating a catalytical amount of RhB is sufficient to catalyze the reduction of Pt(IV) complexes upon irradiation.

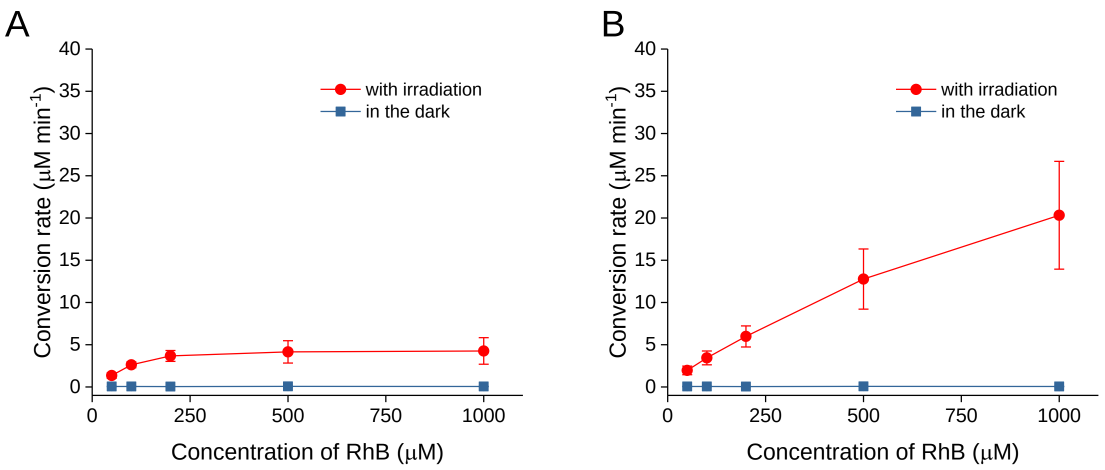

Figure S17. The rate of RhB-catalyzed photoreduction of complex **3c** with different concentrations of catalyst (RhB) upon (A) low-dose (4 mW cm^{-2}) or (B) high-dose (20 mW cm^{-2}) irradiation. In both experiments, the concentrations of complex **3c** and sodium ascorbate are 1000 μM and 2 mM, respectively.

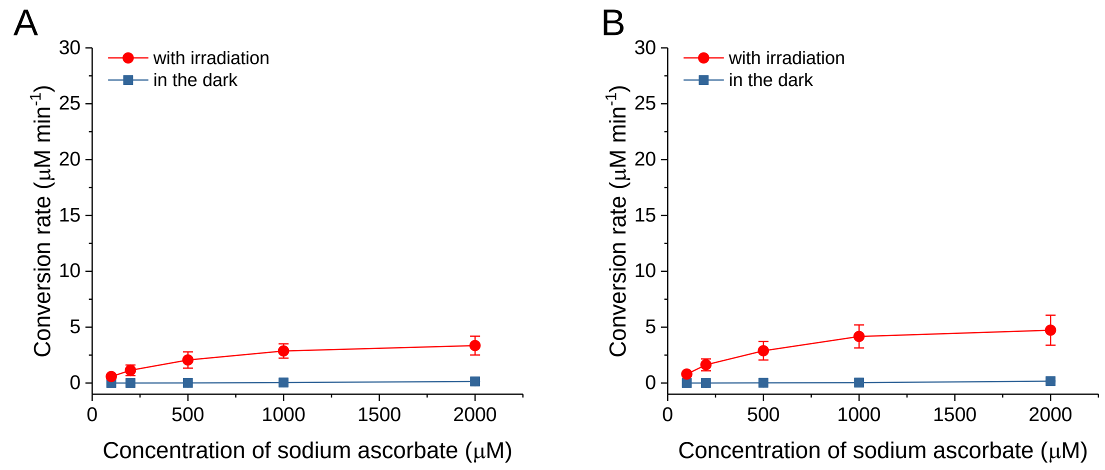

Figure S18. The rate of RhB-catalyzed photoreduction of complex **3c** with different concentrations of sodium ascorbate upon (A) low-dose (4 mW cm^{-2}) or (B) high-dose (20 mW cm^{-2}) irradiation. In both experiments, the concentrations of complex **3c** and RhB are 1000 μM and 100 μM, respectively. The concentrations of substrate and RhB exerted a greater impact on the catalysis rate than that of sodium ascorbate.

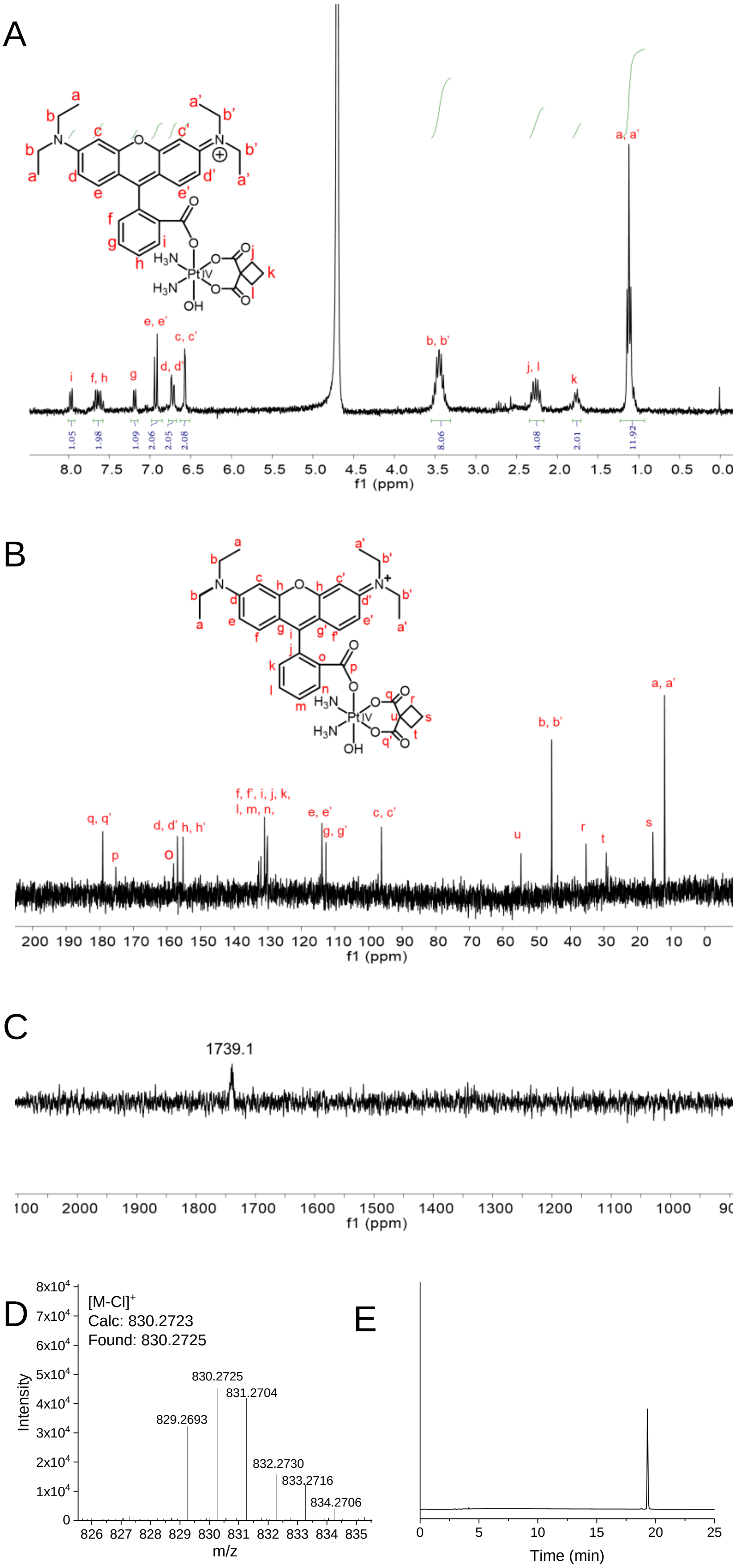

Figure S19. The (A) ^{1}H NMR, (B) ^{13}C NMR, (C) ^{195}Pt NMR, (D) ESI-HRMS (positive mode), and (E) HPLC of rhodaplatin **1.** The NMR samples were dissolved in D_2O.

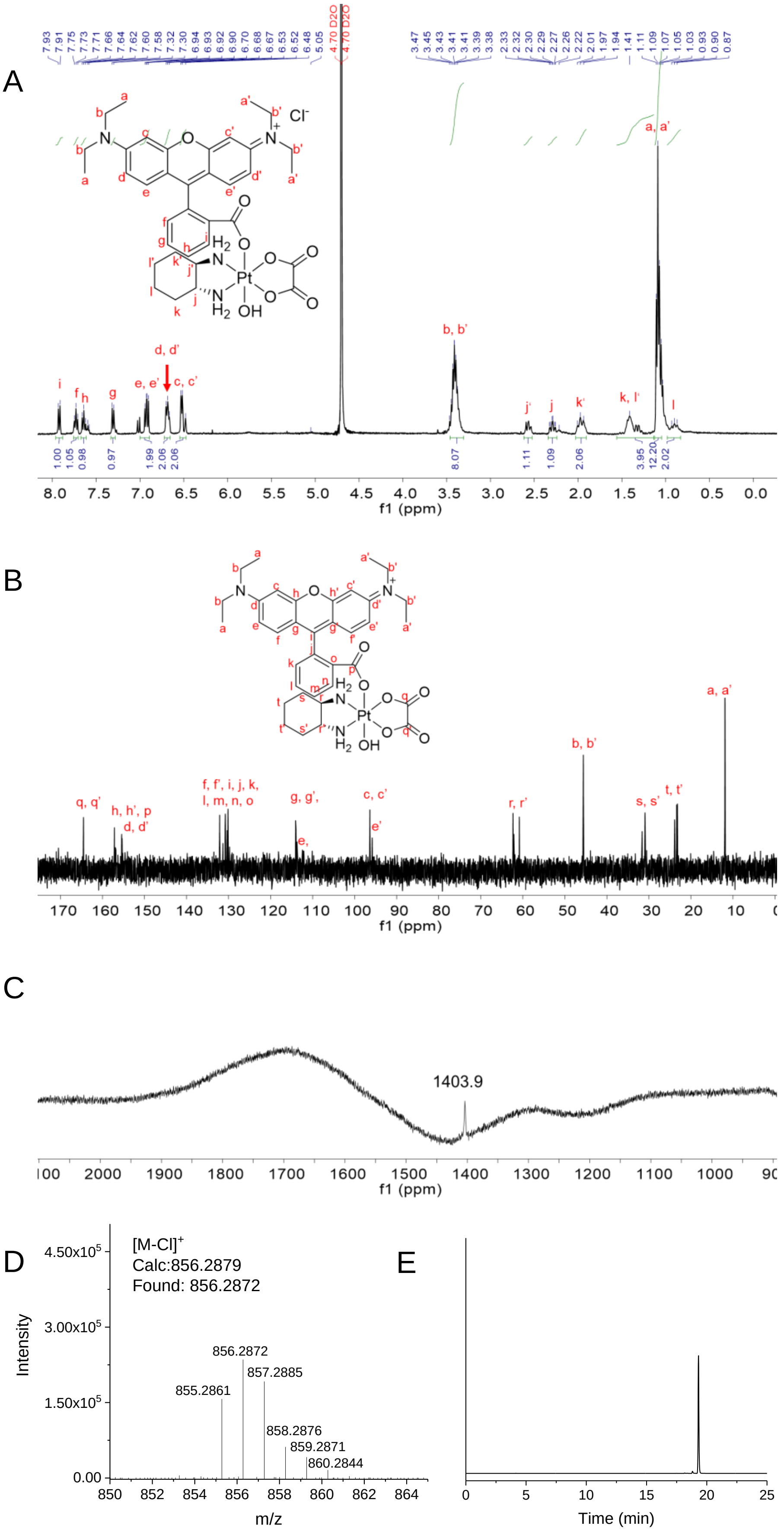

Figure S20. The (A) ^{1}H NMR, (B) ^{13}C NMR, (C) ^{195}Pt NMR, (D) ESI-HRMS (positive mode), and (E) HPLC of rhodaplatin **2.** The NMR samples were dissolved in D_2O.

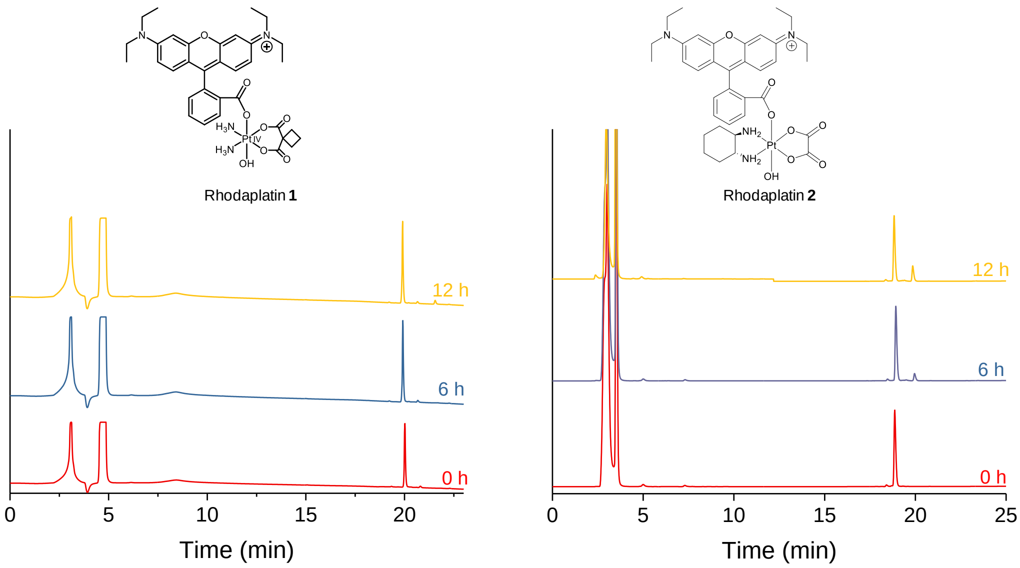

Figure S21. RP-HPLC (254 nm) chromatograms of 100 μM rhodaplatin **1** (left) and 100 μM rhodaplatin **2** (right) in PBS buffer in the presence of 2 mM ascorbate at different time points. The solution was incubated at 37 °C in the dark.

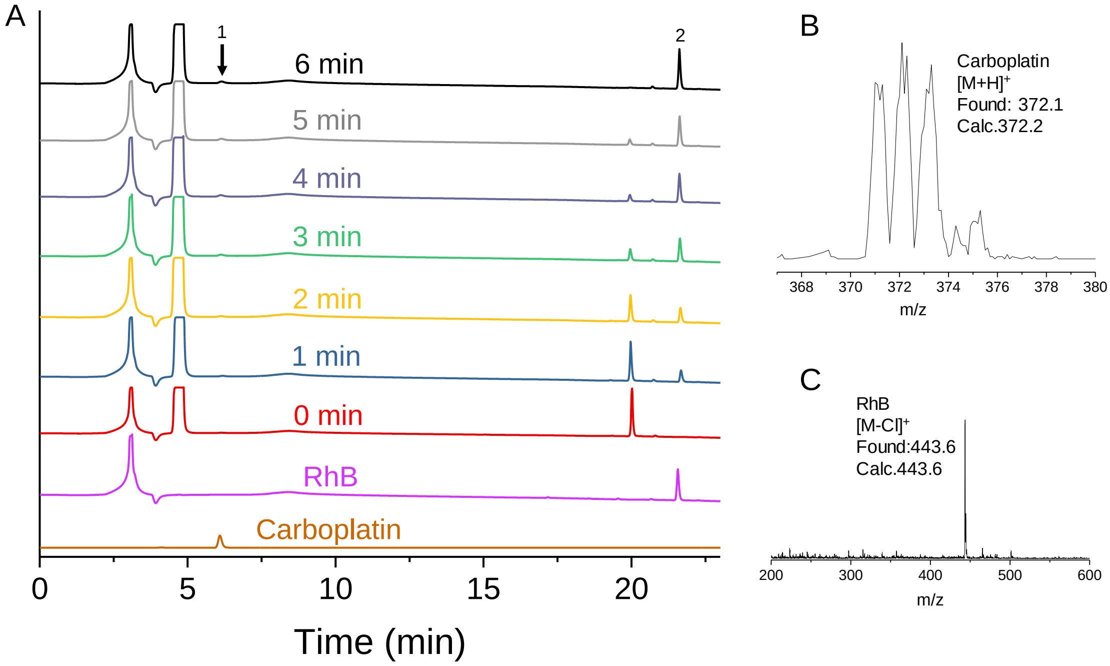

Figure S22. (A) The photo-reduction of rhodaplatin **1** (100 μM) in PBS buffer (pH 7.4) containing 2 mM sodium ascorbate. The HPLC chromatograms were recorded at 254 nm. (B) The ESI-MS spectra of peak 1, which is assigned to carboplatin. (C) The ESI-MS spectra of peak 2, which is assigned to rhodamine B.

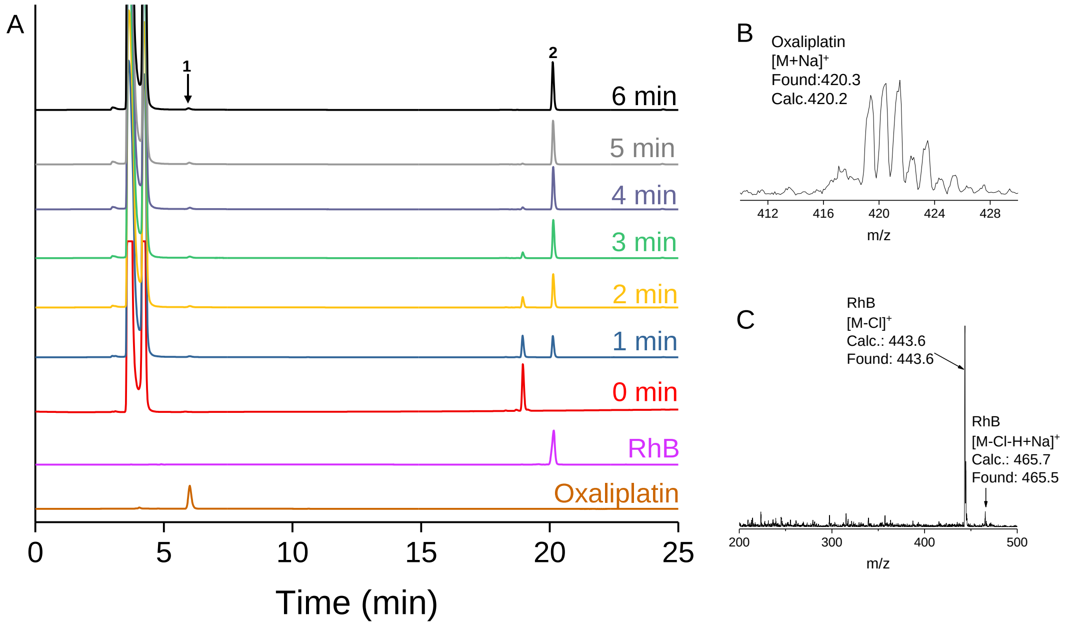

Figure S23. (A) The photo-reduction of rhodaplatin **2** (100 μM) in PBS buffer (pH 7.4) containing 2 mM sodium ascorbate. The HPLC chromatograms were recorded at 254 nm. (B) The ESI-MS spectra of peak 1, which is assigned to oxaliplatin. (C) The ESI-MS spectra of peak 2, which is assigned to rhodamine B.

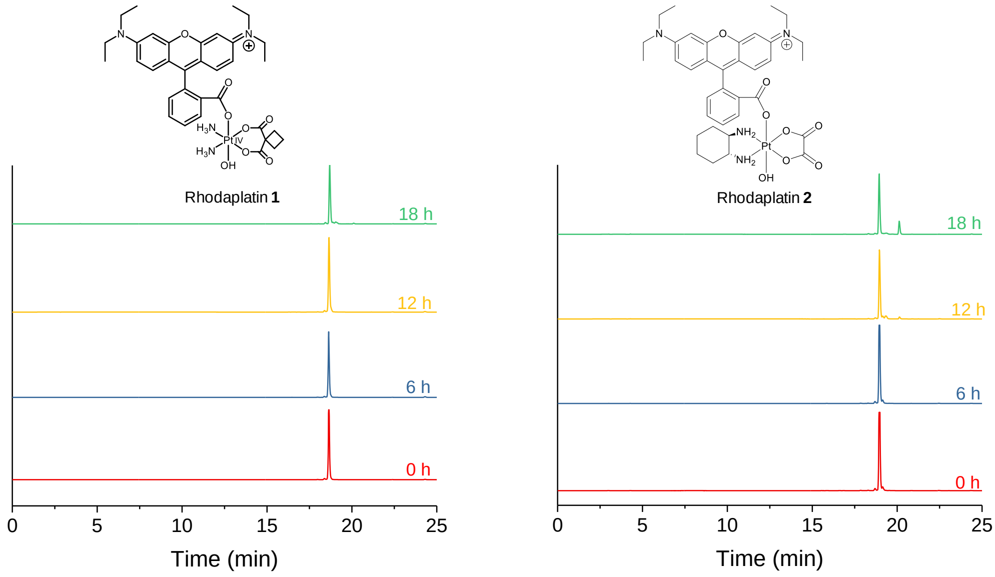

Figure S24. The stability of (left) 100 μM rhodaplatin **1** and (right) 100 μM rhodaplatin **2** in the PBS buffer (10 mM Na_2HPO_4, 2 mM KH_2PO_4, 137 mM NaCl, 2.7 mM KCl, pH = 7.4) containing 2 mM GSH at 310 K.

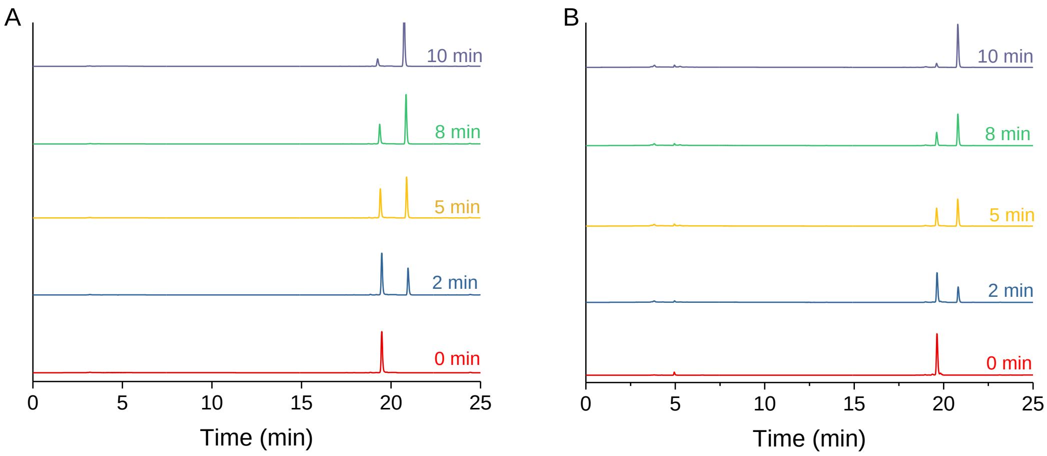

Figure S25. The photoreduction of (A) rhodaplatin **1** (100 μM) and (B) rhodaplatin **2** (100 μM) in PBS buffer (pH 7.4) containing 2 mM GSH. The solution was irradiated with white light (400-760 nm, 4 mW cm^{-2}).

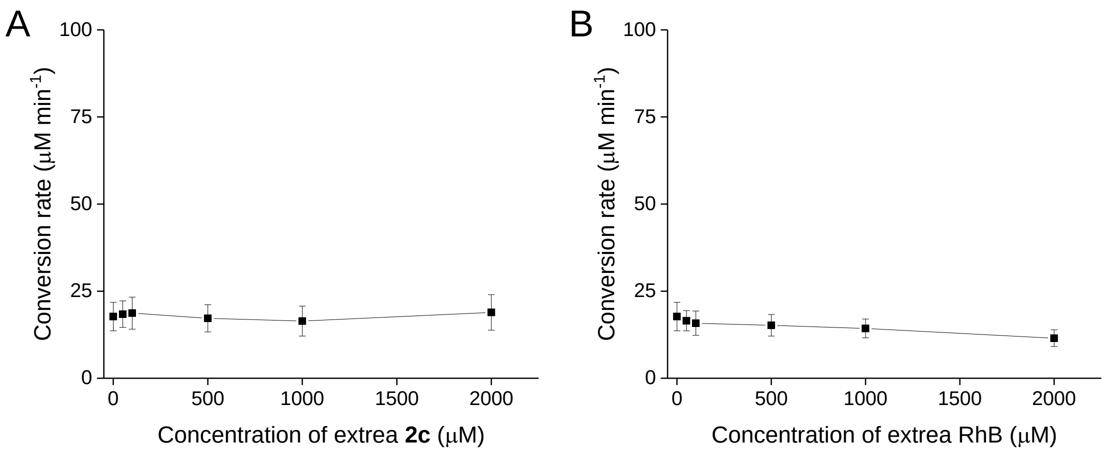

Figure S26. The dependency of conversion rate of rhodaplatin **1** on extra (A) Pt(IV) substrate (complex **2c**) or (B) photocatalyst (RhB). The concentration of rhodaplatin **1** and sodium ascorbate is 100 μM and 2 mM, respectively. The photoconversion rate was studied at increased substrate or photocatalyst concentration (50, 100, 200, 500,1000, and 2000 μM) in PBS buffer (pH 7.4). For all the experiments, the conversion rate was calculated after 1 min white light irradiation (400-760 nm, 4 mW cm^{-2}). In figure S26B, the conversion rate of rhodaplatin **1** slightly decreased with the increased concentration of RhB, which may because of the competitive photoexcitation of RhB and rhodaplatin **1**, which makes less rhodaplatin can be excited at the current power density.

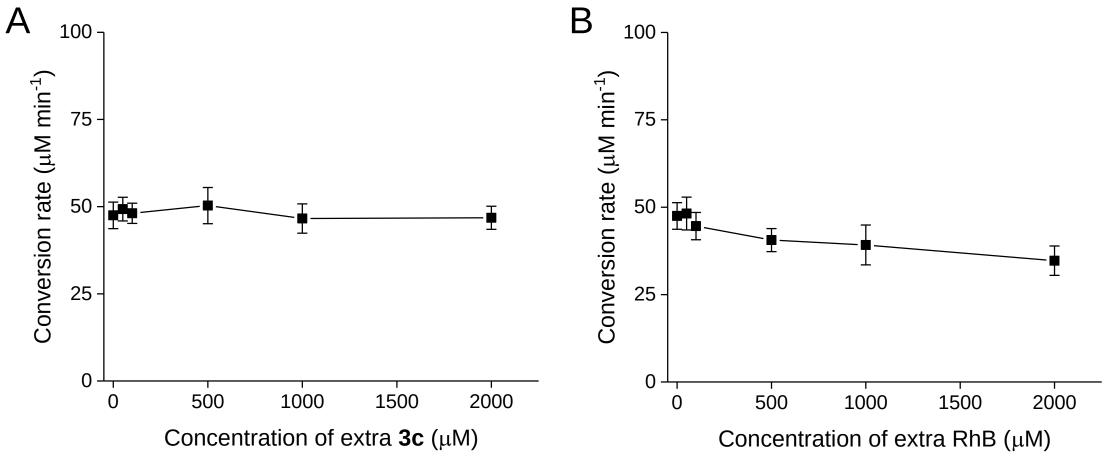

Figure S27. The dependency of conversion rate of rhodaplatin **2** on extra (A) Pt(IV) substrate (complex **3c**) or (B) photocatalyst (RhB). The concentration of rhodaplatin **2** and sodium ascorbate is 100 µM and 2 mM, respectively. The photoconversion rate was studied at increased substrate or photocatalyst concentration (50, 100, 200, 500,1000, and 2000 µM) in PBS buffer (pH 7.4). For all the experiments, the conversion rate was calculated after 1 min white light irradiation (400-760 nm, 4 mW cm^{-2}). In figure S27B, the conversion rate of rhodaplatin **2** slightly decreased with the increased concentration of RhB, which may because of the competitive photoexcitation of RhB and rhodaplatin **2**, which makes less rhodaplatin can be excited at the current power density.

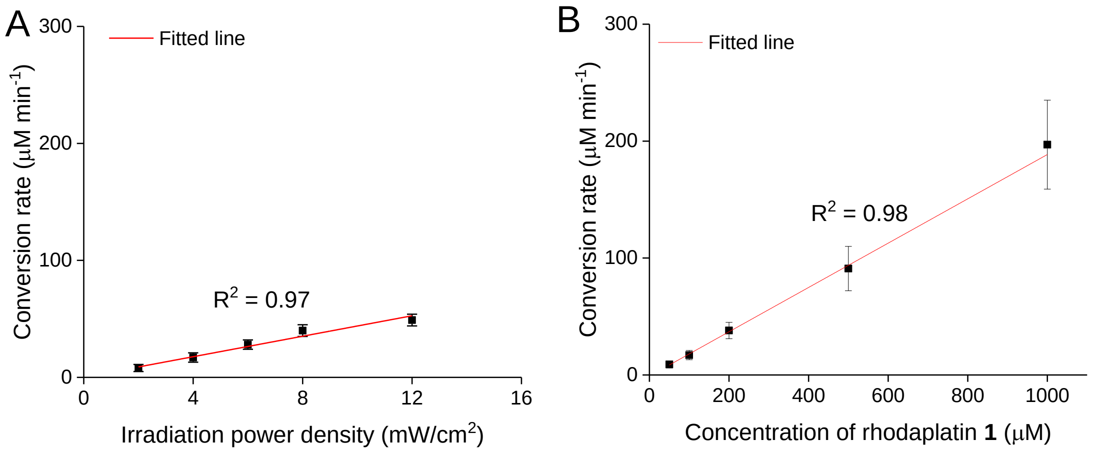

Figure S28. (A) The dependence of conversion rate of rhodaplatin **1** on the irradiation power density. Rhodaplatin **1** (100 µM) was dissolved in the PBS buffer (pH 7.4) containing 2 mM sodium ascorbate. The solution was irradiated with white light at different power densities for 1 min. (B) The dependence of conversion rate of rhodaplatin **1** on the concentration of rhodaplatin **1**. Rhodaplatin **1** at different concentrations was dissolved in the PBS buffer (pH 7.4) containing 2 mM sodium ascorbate. The solution was irradiated with white light (400-760 nm, 4 mW cm^{-2}) for 1 min. R^2 is the coefficient of determination; $R^2 \geq 0.95$ means the conversion rate increased linearly.

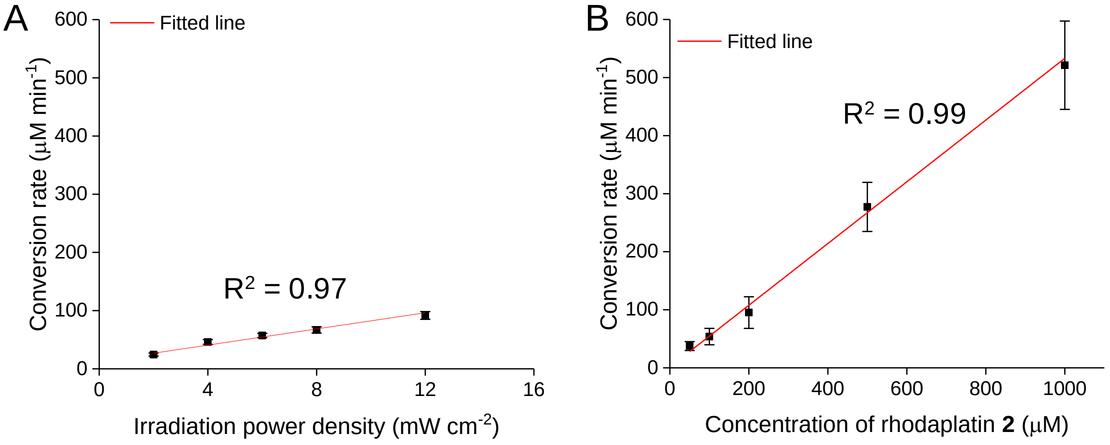

Figure S29. (A) The dependence of conversion rate of rhodaplatin **2** on the irradiation power density. Rhodaplatin **2** (100 μM) was dissolved in the PBS buffer (pH 7.4) containing 2 mM sodium ascorbate. The solution was irradiated with white light at different power densities for 1 min. (B) The dependence of conversion rate of rhodaplatin **2** on the concentration of rhodaplatin **2**. Rhodaplatin **2** at different concentrations was dissolved in the PBS buffer (pH 7.4) containing 2 mM sodium ascorbate. The solution was irradiated with white light (400-760 nm, 4 mW cm^{-2}) for 1 min. R^2 is the coefficient of determination; $R^2 \geq 0.95$ means the conversion rate increased linearly.

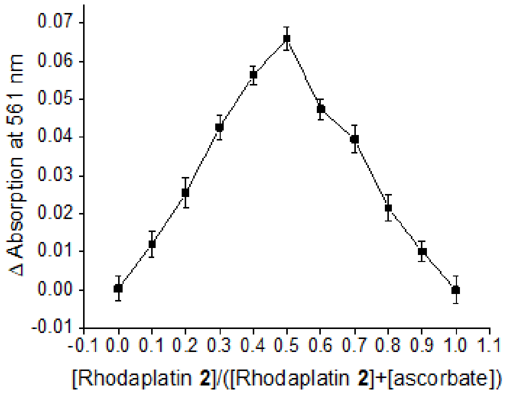

Figure S30. Absorption change of rhodaplatin **2**-ascorbate ion-pair against rhodaplatin **2** only. For solutions containing a mixture of rhodaplatin **2** and ascorbate, the concentrations of rhodaplatin **2**/ascorbate are 0/100, 10/90, 20/80, 30/70, 40/60, 50/50, 60/40, 70/30, 80/20, 90/10, and 100/0 μM. For solutions containing rhodaplatin **2** only, 0, 10, 20, 30, 40, 50, 60, 70, 80, 90, and 100 μM rhodaplatin **2** were used in the solutions. The Y-axis indicates the difference of absorption at 561 nm between the mixture and rhodaplatin **2** only. The difference of absorption reached the maximum when the mole fraction of rhodaplatin **2** is 50%, indicating the stoichiometry of rhodaplatin **2**-ascorbate ion-pair is 1:1.

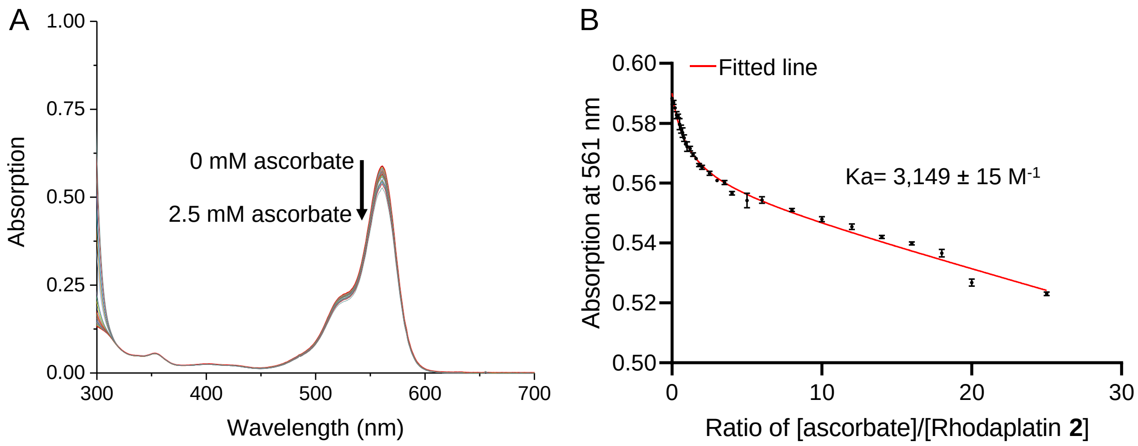

Figure S31. (A) UV-Vis titration of rhodaplatin **2** (100 µM) with ascorbate (0 - 25 equiv.) in Milli-Q water at 25 °C. (B) The changes of absorption at 561 nm with the increased concentration ratio of ascorbate and rhodaplatin **2**. Ka is the calculated association constant of rhodaplatin **2** with ascorbate.

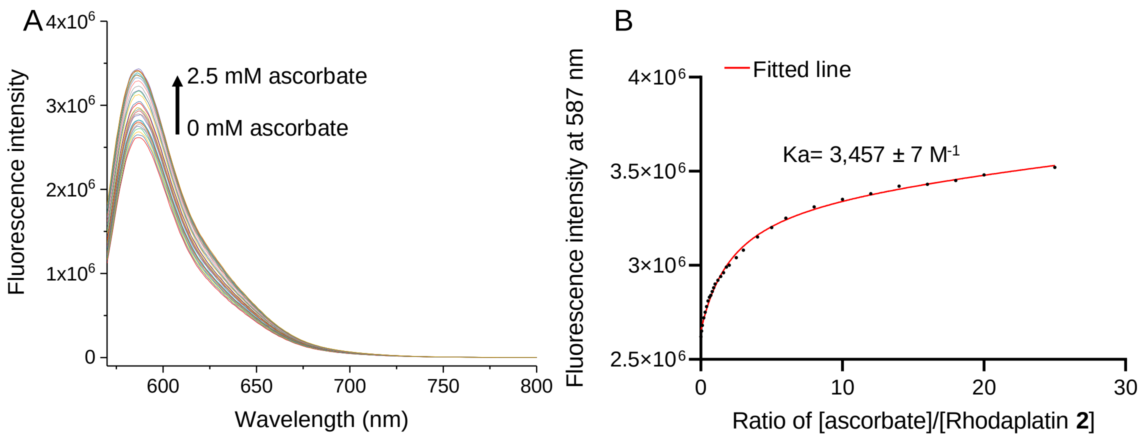

Figure S32. (A) Fluorescence titration of rhodaplatin **2** (100 µM) with ascorbate (0 - 25 equiv.) in Milli-Q water at 25 °C. (B) The changes of fluorescence intensity at 587 nm with the increased concentration ratio of ascorbate and rhodaplatin **2**. Ka is the calculated association constant of rhodaplatin **2** with ascorbate.

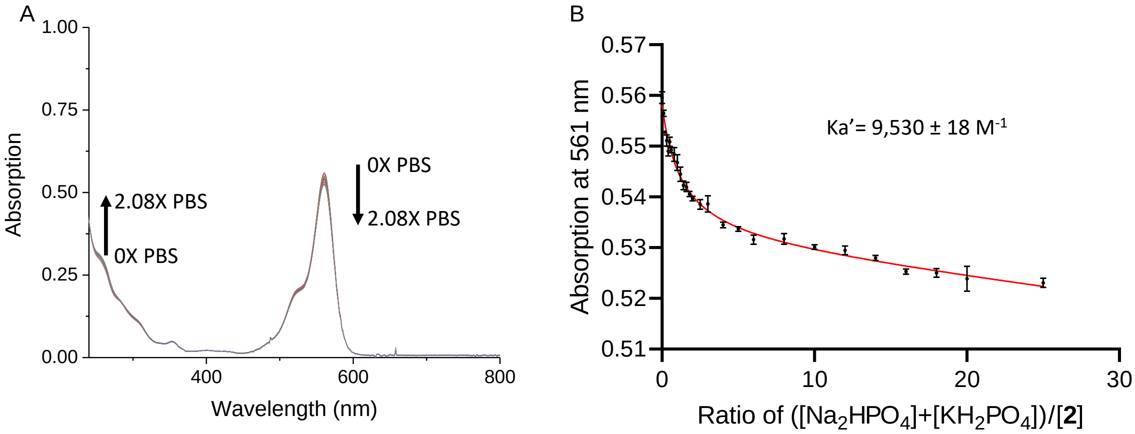

Figure S33. (A) UV-Vis titration of rhodaplatin **2** (100 µM) with PBS buffer (0 – 2.08X PBS) in Milli-Q water at 25 °C. (B) The changes of absorption at 561 nm with the increased concentration ratio of PBS and rhodaplatin **2**. The components in 1X PBS buffer are 10 mM Na_2HPO_4, 2 mM KH_2PO_4, 137 mM NaCl, and 2.7 mM KCl (pH 7.4). The concentration ratio of Na_2HPO_4+KH_2PO_4/rhodaplatin **2** was used for calculation. Ka' is the calculated association constant of rhodaplatin **2** with phosphates.

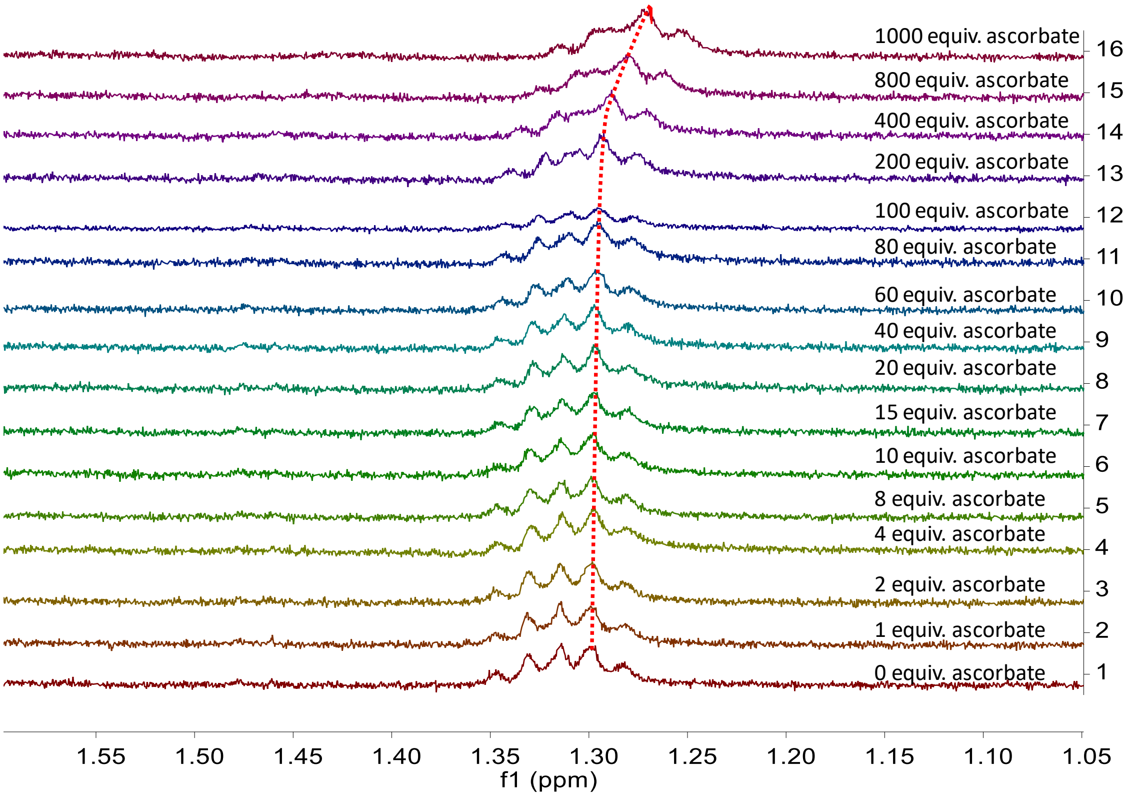

Figure S34. The partial ^{1}H NMR spectra of rhodaplatin **2** (400 µM) in 4X PBS buffer (40 mM Na_2HPO_4, 8 mM KH_2PO_4, 548 mM NaCl, 10.8 mM KCl, pH 7.4, at 25 °C; D_2O as the solvent), and the solution was titrated with sodium ascorbate. Because the signal of rhodaplatin **2** at low concentration is weak, we increased the concentration of rhodaplatin **2** and PBS accordingly. No significant chemical shift was observed until 200 equiv. ascorbate (80 mM) was added, which is much higher than the concentration of ascorbate we used in other experiments (up to 80 equiv. ascorbate, or 8 mM), indicating that most rhodaplatins are not presented as rhodaplatin-ascorbate ion pairs in PBS buffer. Tetramethylsilan (TMS) was used as the reference of chemical shift (δ_{TMS} = 0 ppm).

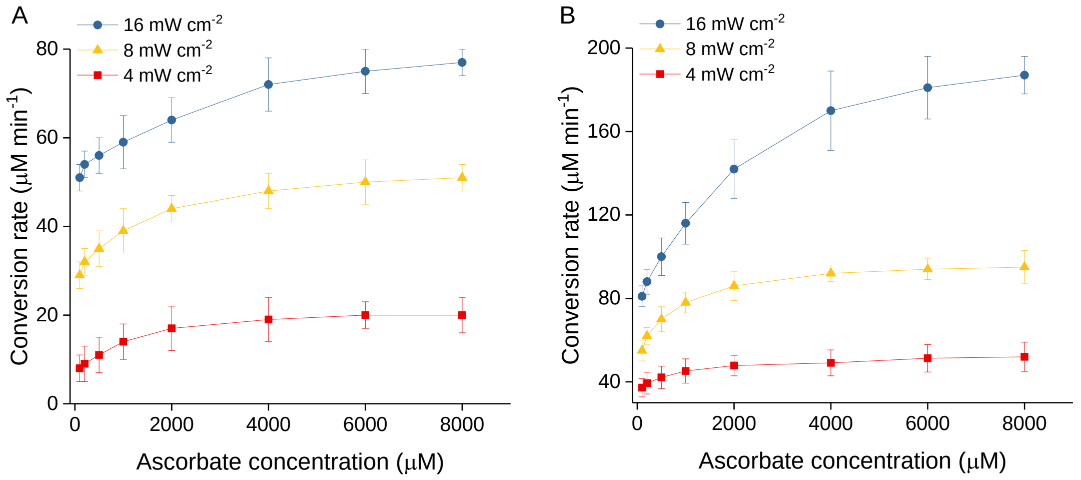

Figure S35. The dependence of conversion rate of (A) rhodaplatin **1** and (B) rhodaplatin **2** on the concentration of sodium ascorbate in PBS buffer (pH 7.4) upon different irradiation conditions. The concentration of rhodaplatin is 100 µM. When the irradiation intensity is 16 mW cm^{-2}, the solution was irradiated for 0.5 min to avoid the possibility that rhodaplatins might be fully photoreduced before the completion of irradiation. In other irradiation conditions, the solution was irradiated for 1 min.

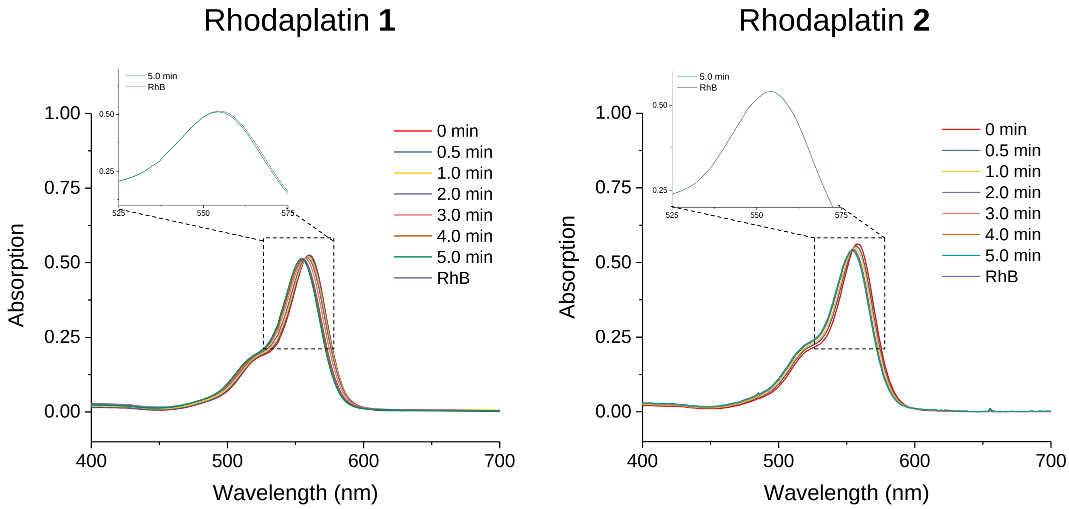

Figure S36. The change of UV-Vis absorption of the photoreduction process of rhodaplatin **1** (left) and rhodaplatin **2** (right) in PBS buffer (pH 7.4) in the presence of sodium ascorbate (2 mM). Rhodamine B (100 µM) was used as a control. The concentration of rhodaplatin is 100 µM. The solution was irradiated with white light (400-760 nm, 4 mW cm^{-2}).

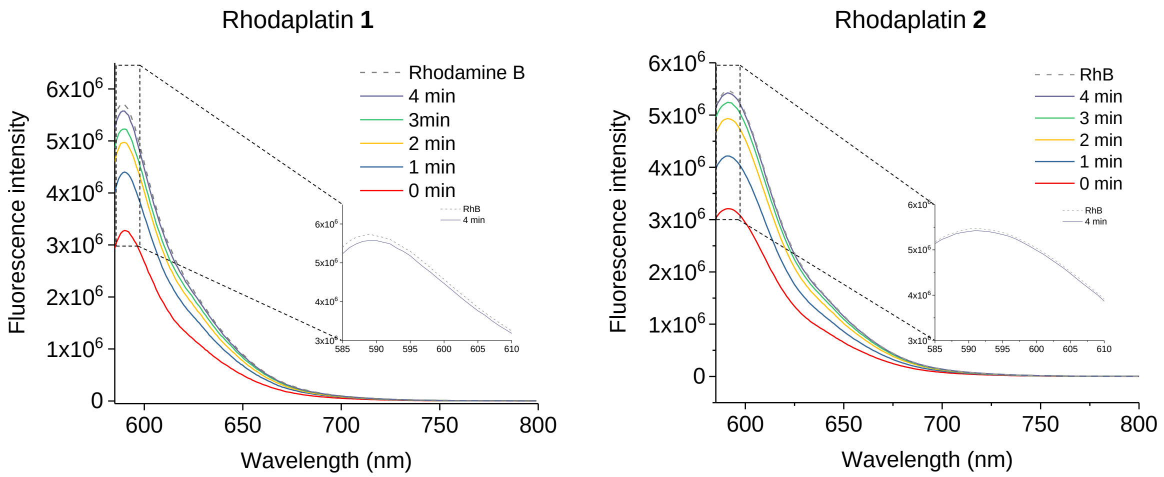

Figure S37. The fluorescence spectrum of rhodaplatin **1** (left) and rhodaplatin **2** (right) in the presence of sodium ascorbate upon irradiation. Rhodamine B was used as a control. The concentration of rhodaplatin, rhodamine B, and sodium ascorbate is 100 µM, 100 µM, and 2 mM, respectively. All the complexes were dissolved in PBS buffer (pH 7.4). The excitation wavelength is 570 nm. The solution was irradiated with white light (400-760 nm, 4 mW cm^{-2}).

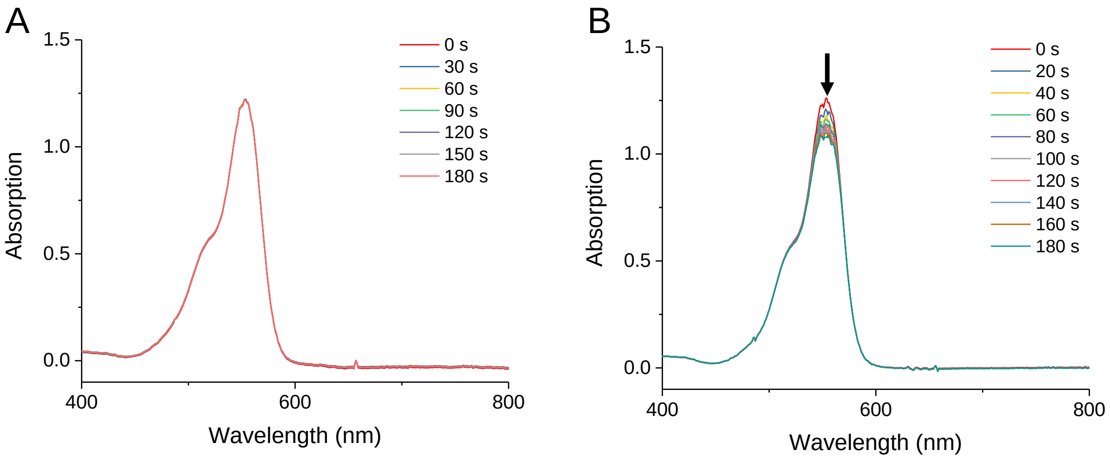

Figure S38. (A) The absorption spectra of rhodamine B (100 µM) in PBS buffer (pH 7.4) upon visible light irradiation. (B) The absorption spectra of rhodamine B (100 µM) in PBS buffer (pH 7.4) containing 2 mM sodium ascorbate upon visible light irradiation. The solution was irradiated with white light (400-760 nm, 4 mW cm^{-2}).

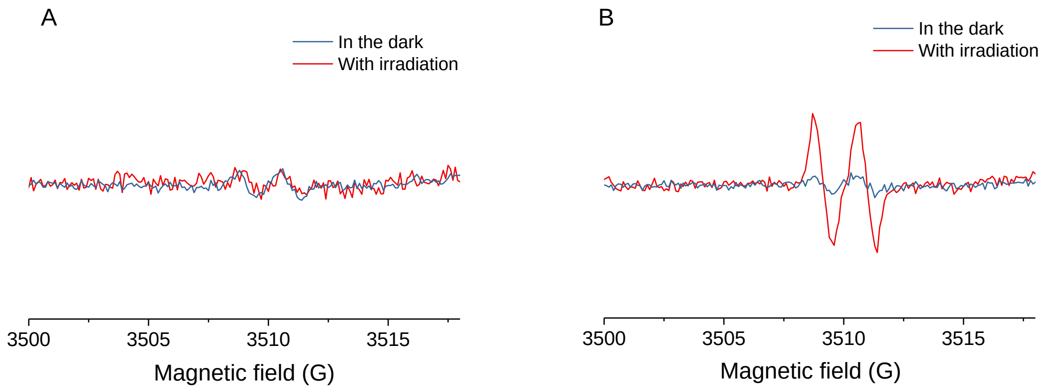

Figure S39. EPR spectrum of (A) PBS buffer (pH 7.4) containing 20 mM sodium ascorbate and (B) PBS buffer (pH 7.4) containing 20 mM sodium ascorbate and 1 mM rhodaplatin **2** with or without irradiation with white light for 1 min. The EPR spectral parameters of detected ascorbate radicals are g value = 2.0052; a^H = 1.8 G, which are consistent with other reports.[22]

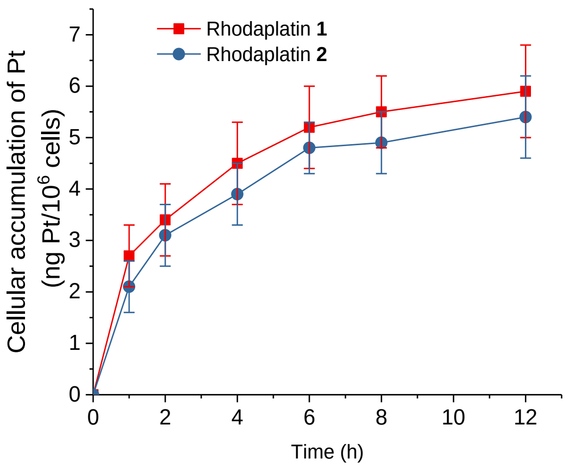

Figure S40. Cellular accumulation of Pt in A2780cisR cells. Cells were treated with rhodaplatin **1** (10 μM) or rhodaplatin **2** (10 μM) for 12 h. Cells were collected at different time points, the intracellular Pt amount was measured by ICP-MS.

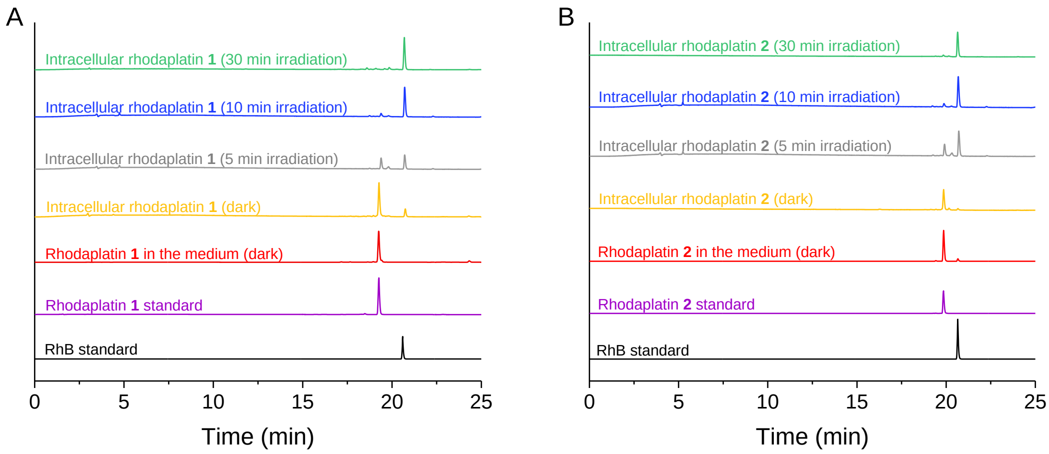

Figure S41. The stability and photoactivation of (A) rhodaplatin **1** and (B) rhodaplatin **2** in the culture medium and A2780cisR cells. Cells were incubated with culture medium containing 50 μM rhodaplatins for 6 h. After incubation, the culture medium is removed and analyzed by HPLC. Cells were washed with PBS buffer twice, incubated with drug-free medium. Cells were kept in the dark or irradiated with white light (400-760 nm, 4 mW cm^{-2}) for 5, 10, and 30 min, respectively. Then, cells were lysed, the lysate solution was analyzed by HPLC. RhB and rhodaplatins were added into the culture medium and analyzed immediately as the standard. To bypass the peaks in the culture medium or cell lysate, HPLC was recorded at 560 nm.

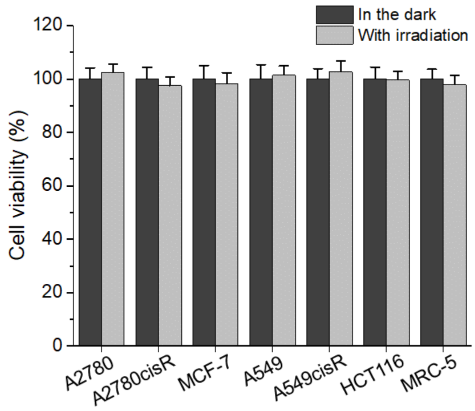

Figure S42. The cell viability of different cell lines with or without visible light irradiation. Cells were irradiated with white light (400-760 nm, 4 mW cm^{-2}) for 30 min, then cultured for another 42 h. The cell viability was detected by MTT assay. For each cell line, the cell viability of the unirradiated group was normalized to 100%.

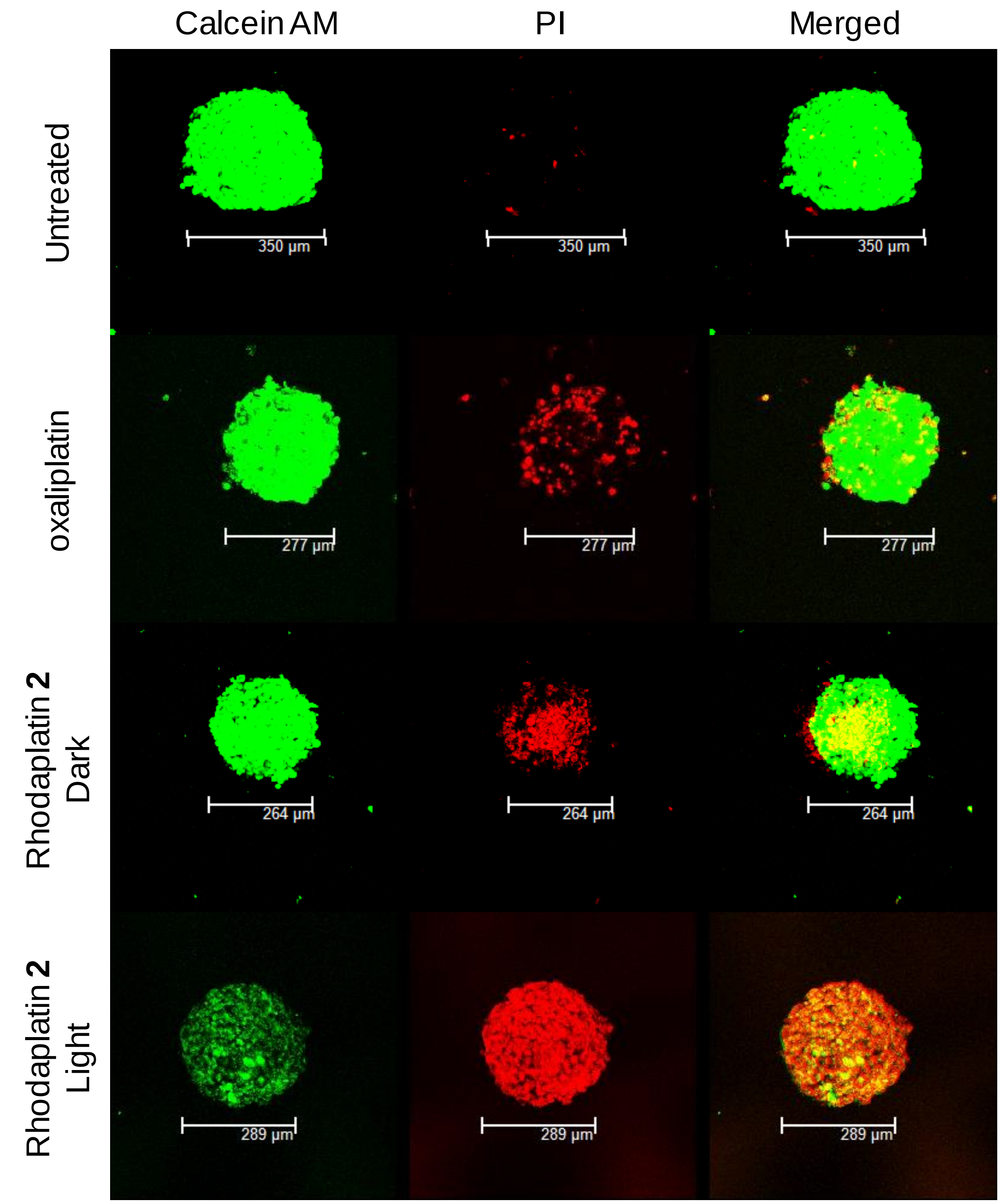

Figure S43. Confocal images of Calcein AM/PI double-stained A2780cisR spheroids. Spheroids were treated with 100 µM complex for 6 h. The drug-containing medium was then replaced with drug-free fresh medium and the cells were irradiated with white light (400-760 nm, 4 mW cm^{-2}) for 0.5 h. The medium was replaced by fresh medium and the cells were incubated for another 18 h.

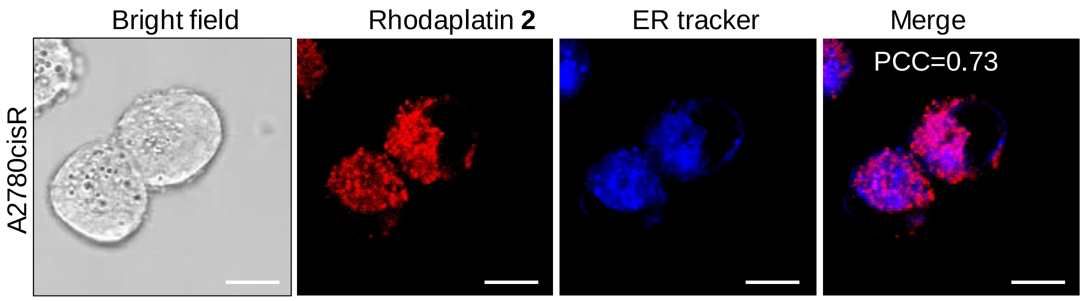

Figure S44. The cellular distribution of rhodaplatin **2** in A2780cisR cells. Cells were treated with rhodaplatin **2** (10 μM) for 6 h, then co-stained with ER tracker and visualized by a confocal microscope. Scale bar represents 10 μm.

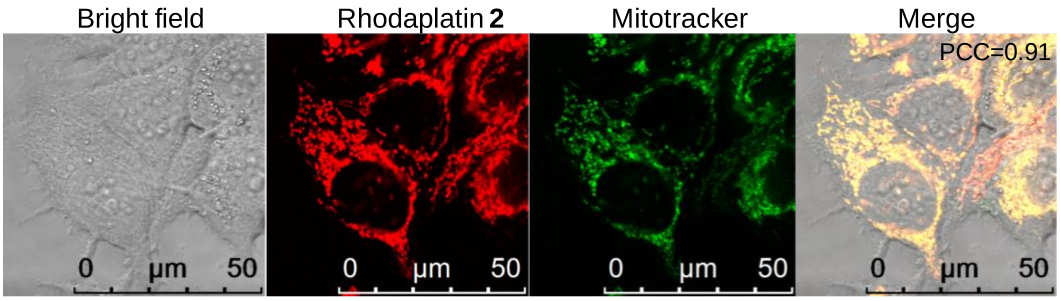

Figure S45. The cellular accumulation of rhodaplatin **2** in MCF-7 cells. Cells were treated with rhodaplatin **2** (10 μM) for 6 h, then co-stained with mitotracker and visualized by confocal microscope.

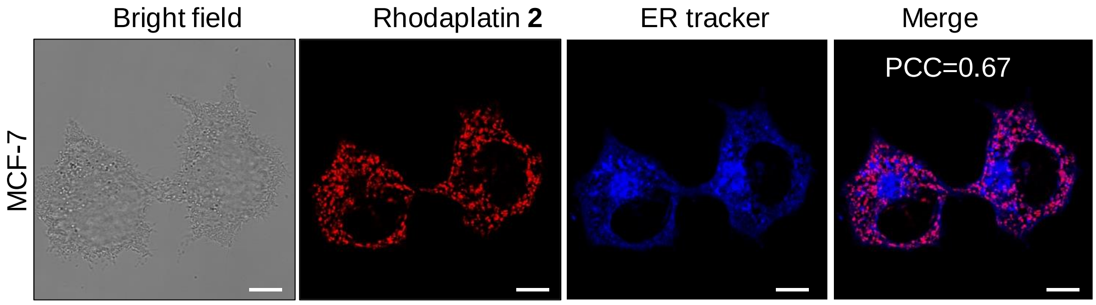

Figure S46. The cellular accumulation of rhodaplatin **2** in MCF-7 cells. Cells were treated with rhodaplatin **2** (10 μM) for 6 h, then co-stained with ER tracker and visualized by a confocal microscope. Scale bar represents 10 μm.

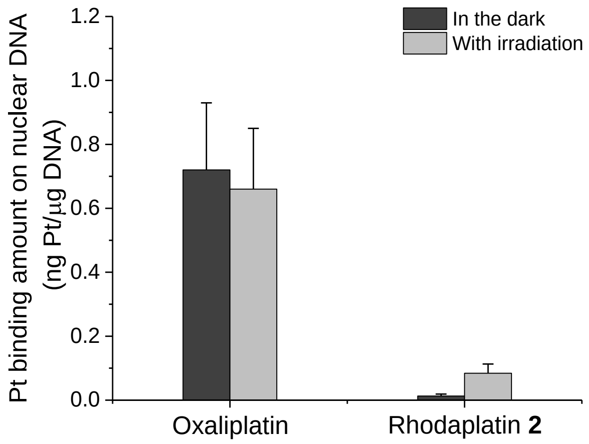

Figure S47. The amount of Pt on nuclear DNA of treated A2780cisR cells. Cells were treated with compound (10 µM) for 6 h, then the culture medium was replaced, and cells were irradiated with or without visible light (400-760 nm, 4 mW cm^{-2}) for 30 min. After irradiation, cells were cultured for another 1 h.

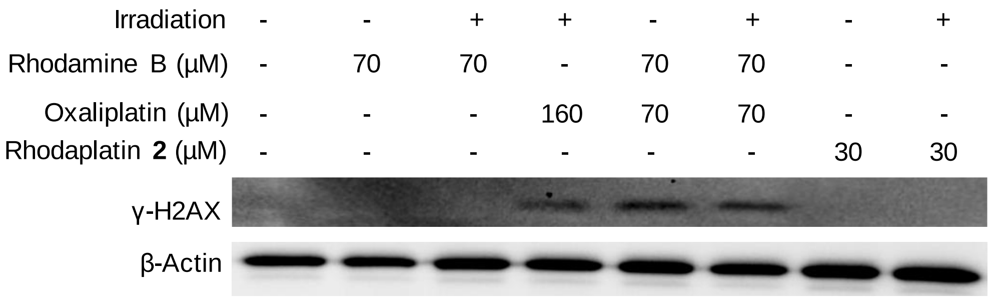

Figure S48. The expression level of γ-H2AX, a well-known marker response for nuclear DNA damage,[23] in A2780cisR cells after different treatment. Cells were treated with complexes at the indicated concentrations for 6 h, the culture medium was replaced with drug-free fresh medium, and cells were irradiated with white light (400-760 nm, 4 mW cm^{-2}) or kept in the dark for 30 min. Then, cells were cultured for another 12 h.

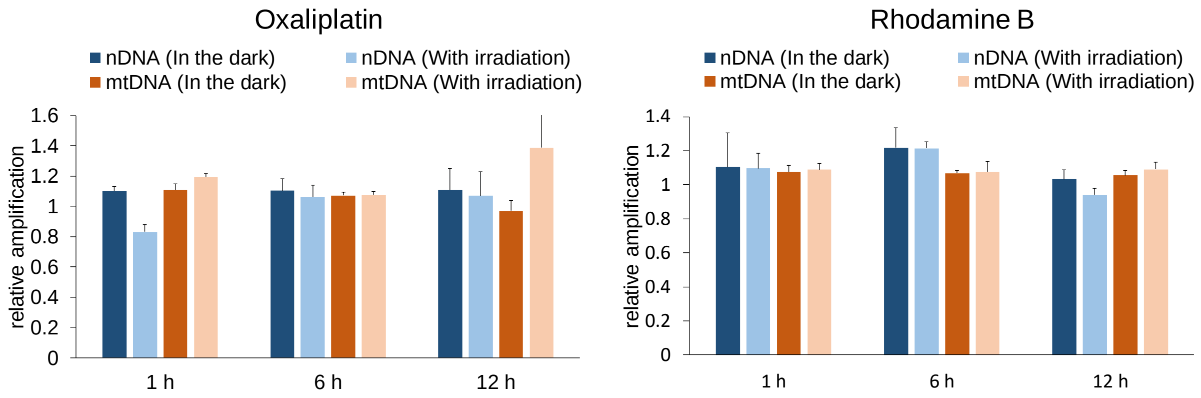

Figure S49. The relative amplification of nuclear DNA and mitochondrial DNA in A2780cisR cells treated with oxaliplatin and rhodamine B. Cells were treated with compound (10 µM) for 6 h, then the culture medium was replaced, and cells were irradiated with or without visible

light (400-760 nm, 4 mW cm^{-2}) for 30 min. After irradiation, cells were cultured for another 1, 6, and 12 h.

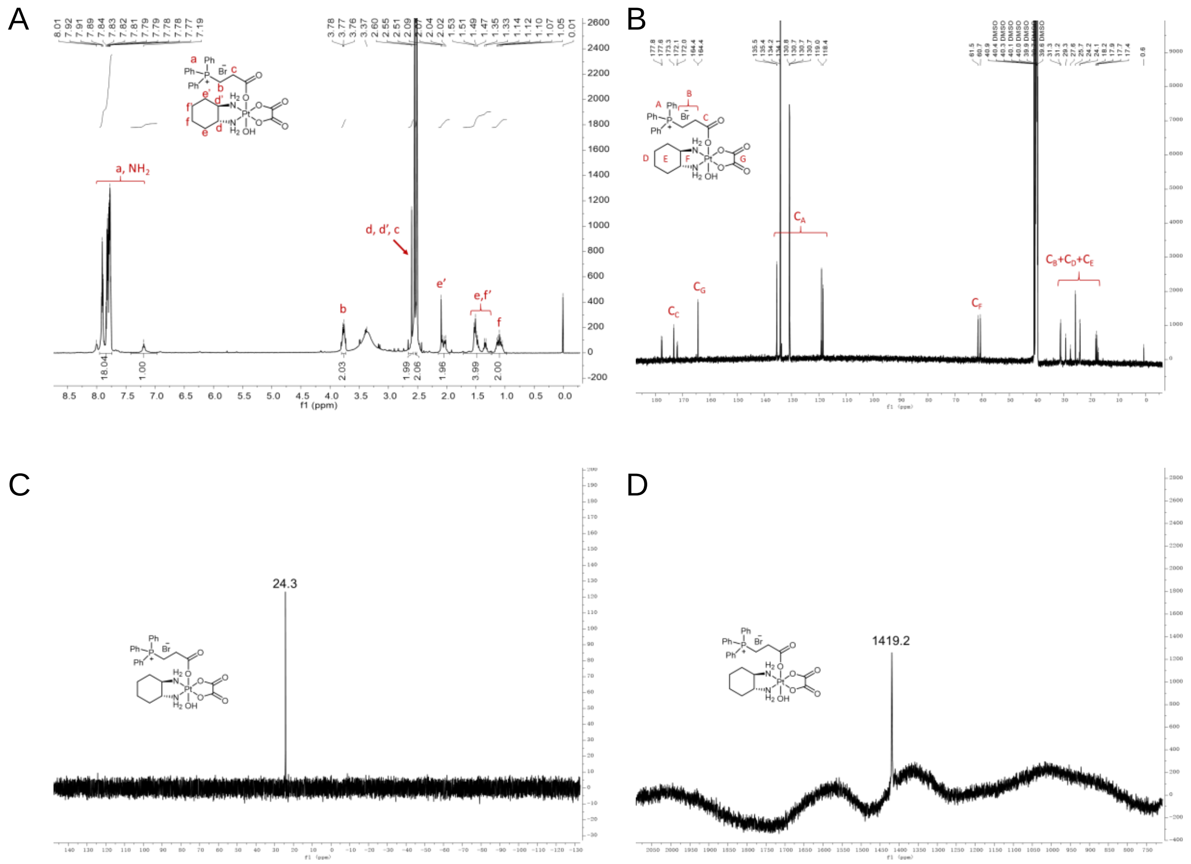

Figure S50. The (A) ^{1}H, (B) ^{13}C, (C) ^{31}P, and (D) ^{195}Pt NMR of complex **4** in DMSO-d_6.

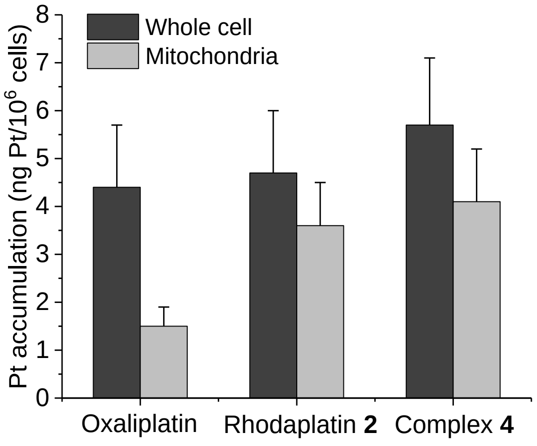

Figure S51. The whole cellular and mitochondrial accumulation amount of oxaliplatin, rhodaplatin **2**, and complex **4** in A2780cisR cells. Cells were treated with complex (10 μM) for 6 h. The Pt concentration was detected by ICP-MS.

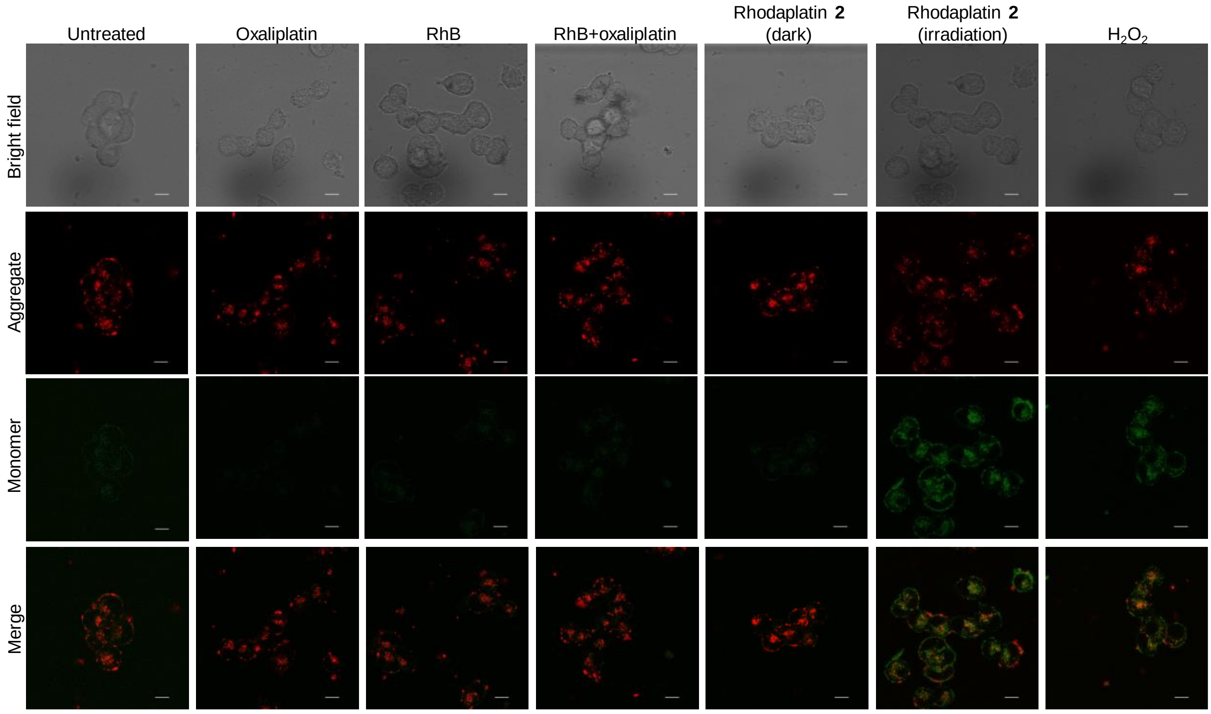

Figure S52. The JC-1 staining in A2780cisR cells with different treatments. Cells were treated compound (10 µM) for 6 h, the culture medium was replaced with drug-free medium, cells were irradiated with or without visible light (400-760 nm, 4 mW cm^{-2}) for 30 min. Cells were cultured for another 18 h. Then, the A2780cisR cells were treated with JC-1 (10 µg mL^{-1}) for 30 min. The JC-1 dye was excited at 488 nm, the aggregate form and monomer form were recorded at 590-610 nm and 520-540 nm, respectively. Cells that pre-treated with H_2O_2 (250 µM) for 6 h were used as positive controls. The increased fluorescence of JC-1 monomers (green channel) indicated the loss of mitochondria membrane potential. Scale bar represents 10 µm.

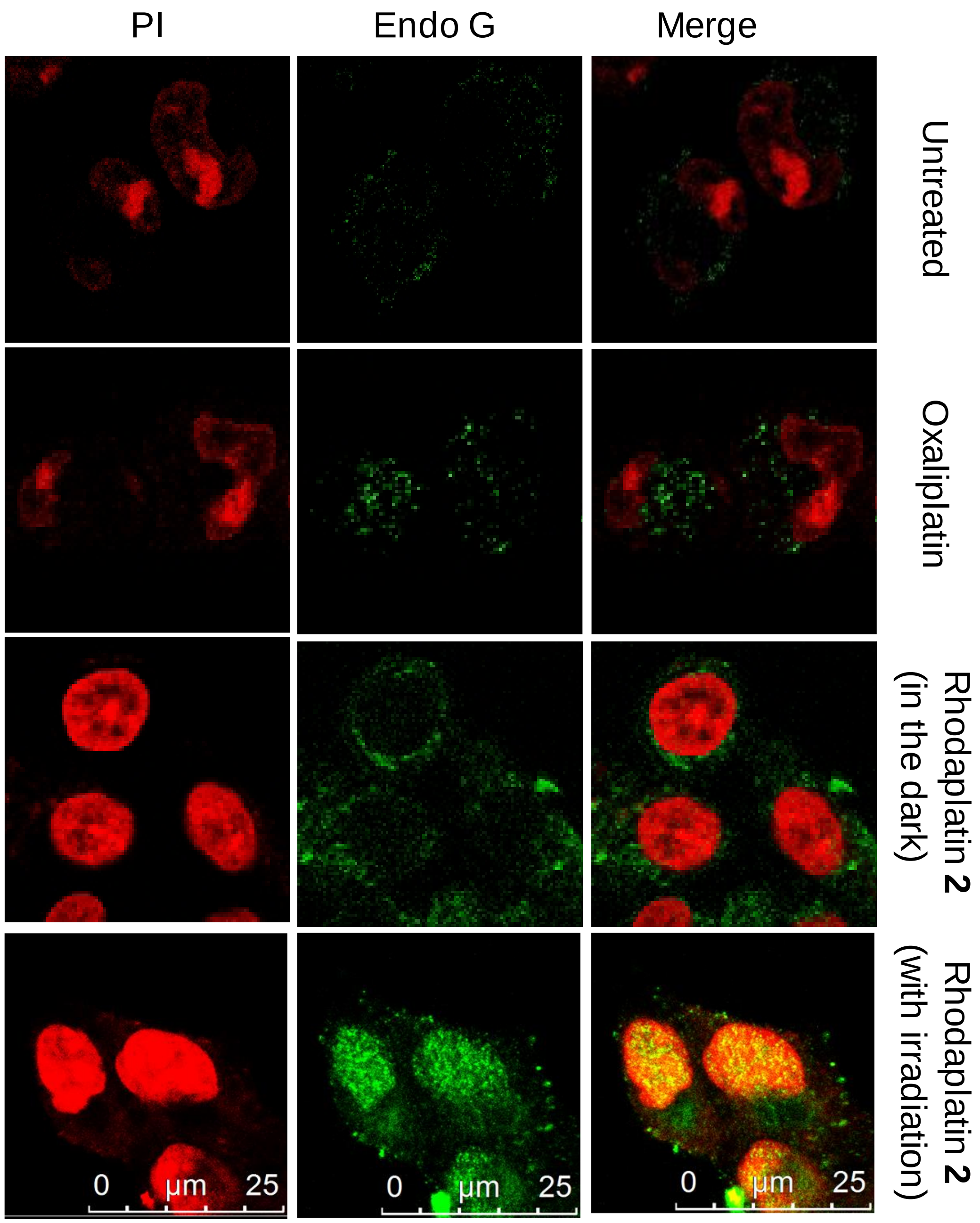

Figure S53. The cellular distribution of endo G in A2780cisR cells after oxaliplatin or rhodaplatin **2** treatment. Cells were treated with oxaliplatin or rhodaplatin **2** (10 μM) for 6 h, then the culture medium was replaced with drug-free fresh medium, cells were irradiated with or without visible light (400-760 nm, 4 mW cm^{-2}) for 30 min, then culture for another 12 h.

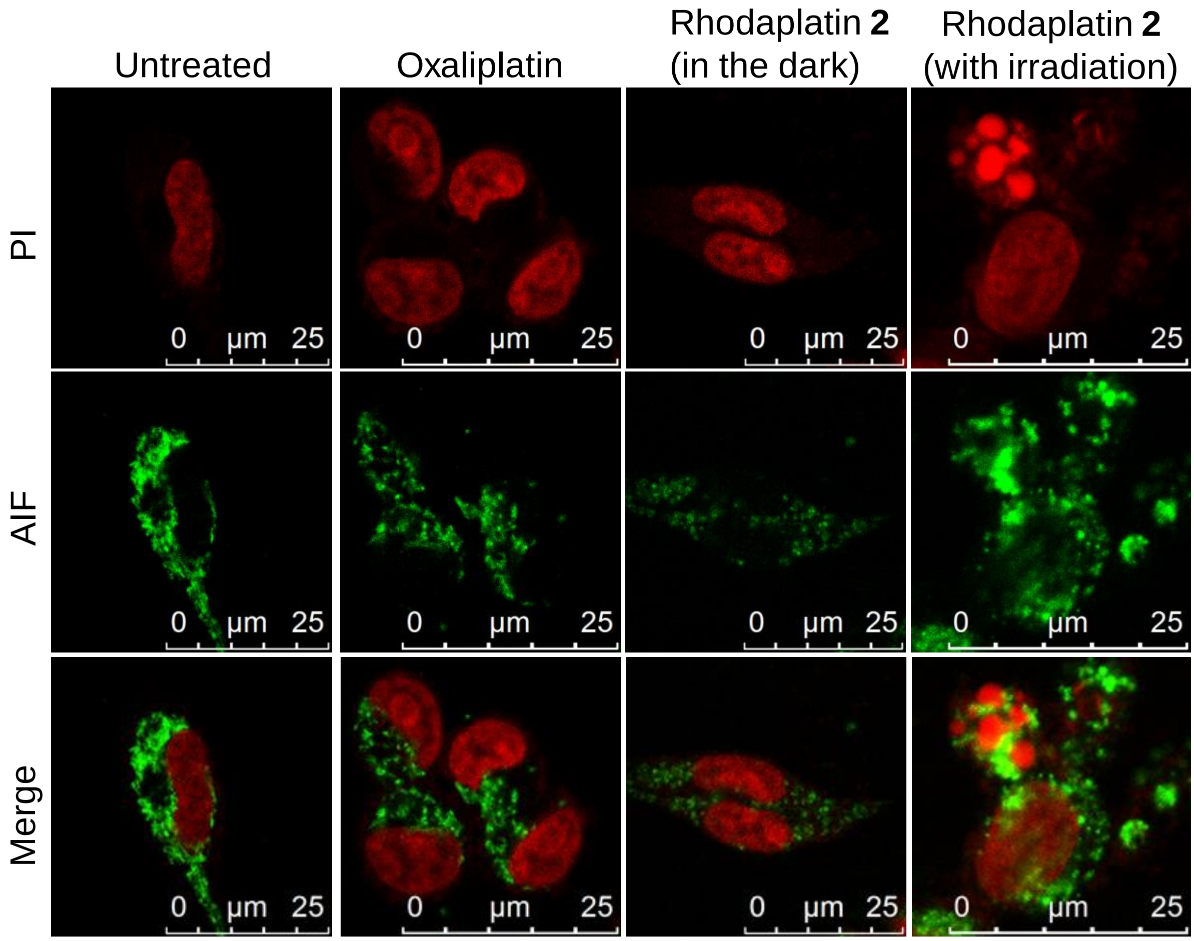

Figure S54. The cellular distribution of AIF in A2780cisR cells after different treatments. Cells were treated with complex (10 μM) for 6 h, then the culture medium was replaced with drug-free fresh medium, cells were irradiated with or without visible light (400-760 nm, 4 mW cm^{-2}) for 30 min, then culture for another 12 h.

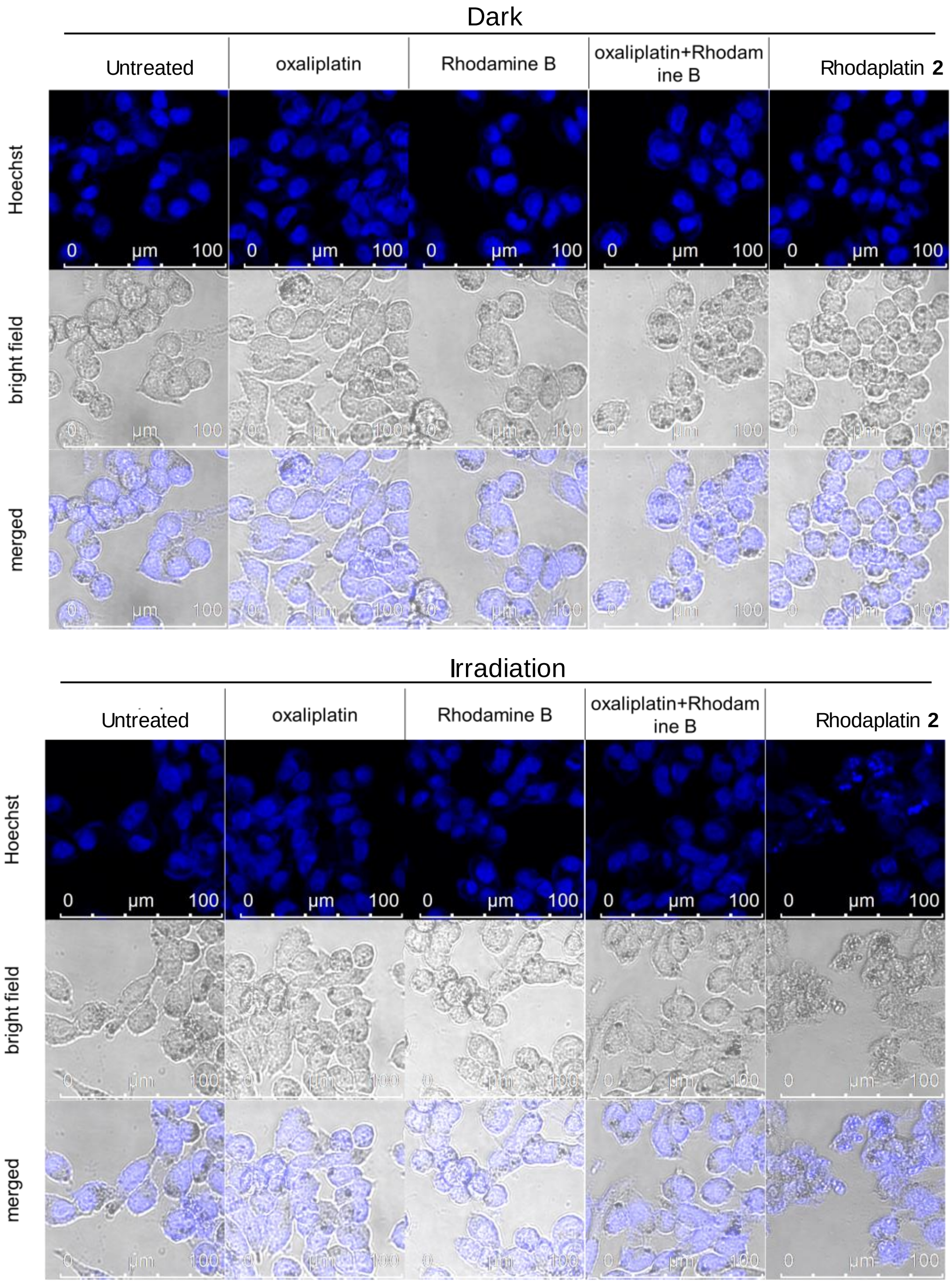

Figure S55. The nuclei morphology of A2780cisR cells after different treatments. Cells were treated with compounds (10 μM) for 6 h, then the culture medium was replaced with drug-free fresh medium, and cells were irradiated without (top) or with (bottom) visible light (400-760 nm, 4 mW cm^{-2}) for 30 min, and cultured for another 18 h.

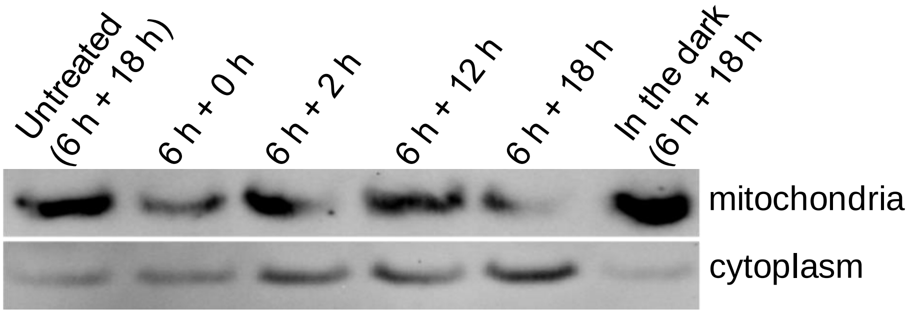

Figure S56. The expression level of cytochrome c in mitochondria and cytoplasm of treated A2780cisR cells. Cells were treated with rhodaplatin **2** (10 µM) for 6 h, then the medium was replaced with fresh drug-free medium, and cells were irradiated with visible light (400-760 nm, 4 mW cm^{-2}) or kept in the dark for 30 min, and cultured for another 0, 2, 12, or 18 h.

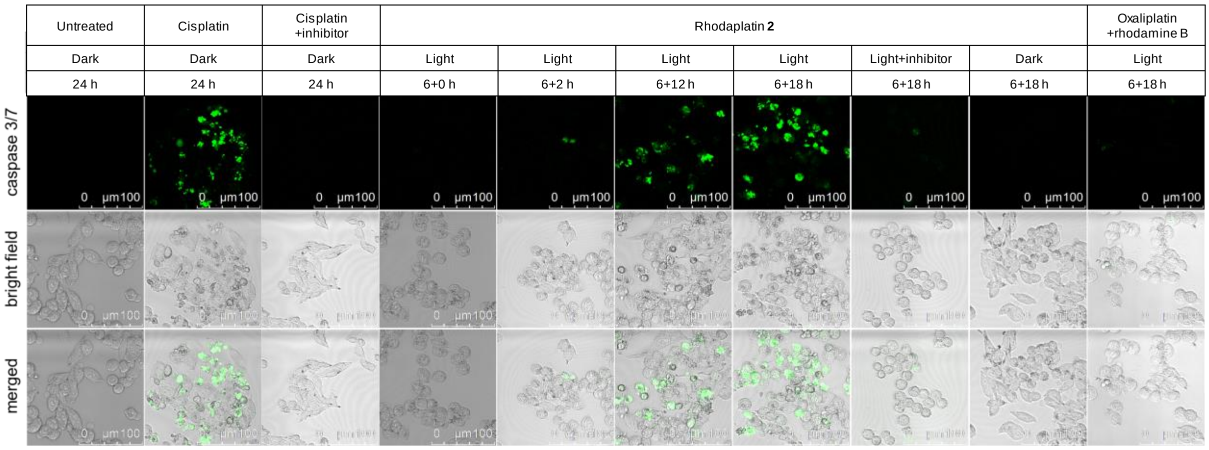

Figure S57. The activation of caspase 3/7 in A2780cisR cells. For the positive control group, cells were treated with cisplatin (50 µM) for 24 h. For rhodaplatin **2** or oxaliplatin + rhodamine B treated groups, cells were treated with rhodaplatin **2** (10 µM) or oxaliplatin (10 µM) + rhodamine B (10 µM) for 6 h, then the culture medium was replaced with drug-free fresh medium, cells were irradiated with or without visible light (400-760 nm, 4 mW cm^{-2}) for 30 min, and cultured for another 0, 2, 12, or 18 h. For cells treated with the inhibitor, cells were pretreated with Z-VAD-FMK (10 µM) for 30 min, before treated with Pt complexes. The green fluorescence indicates the activation of caspase 3/7.

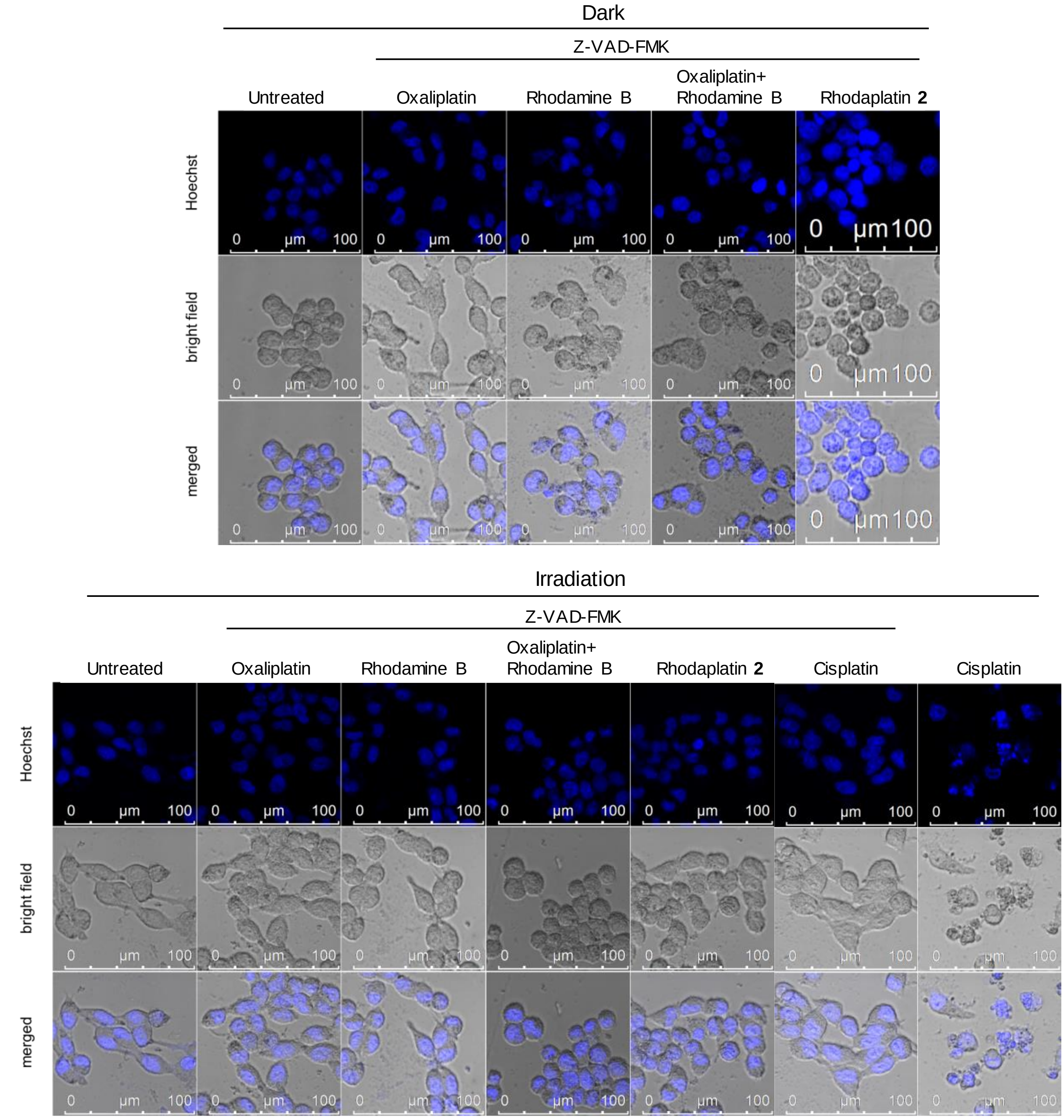

Figure S58. The nuclei morphology of A2780cisR cells after different treatments. For the positive control group, cells were treated with cisplatin (50 μM) for 24 h. In other groups, cells were treated with complex (10 μM) for 6 h, then culture medium was replaced with drug-free fresh medium, cells were irradiated without (top) or with (bottom) visible light (400-760 nm, 4 mW cm^{-2}) for 30 min. Cells were cultured for another 18 h, then stained with Hoechst (10 ng mL^{-1}) for 10 min. For cells treated with Z-VAD-FMK, cells were pretreated with Z-VAD-FMK (10 μM) for 30 min, then treated with different complexes.

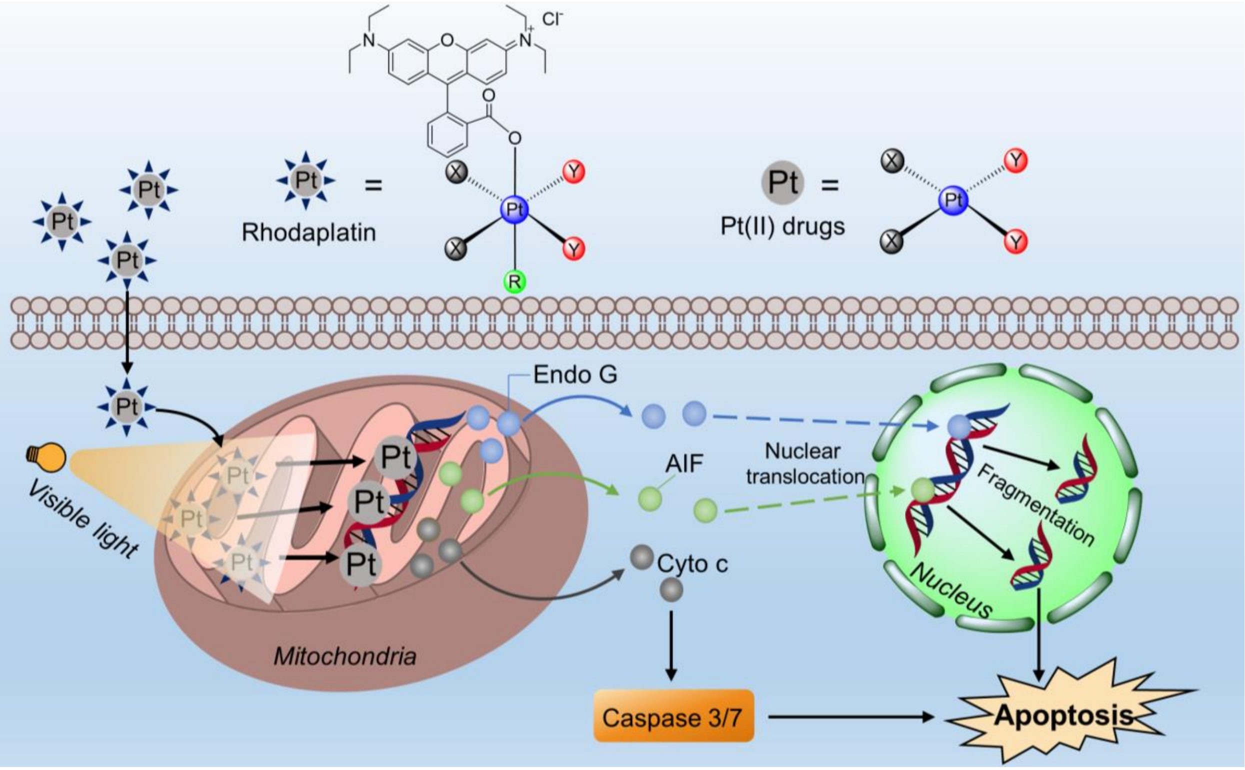

Figure S59. Schematic representation summarizing the cellular mechanism of action of rhodaplatin upon photoactivation.

References

1. Hall, M. D.; Dillon, C. T.; Zhang, M.; Beale, P.; Cai, Z.; Lai, B.; Stampfl, A. P.; Hambley, T. W., The cellular distribution and oxidation state of platinum (II) and platinum (IV) antitumour complexes in cancer cells. *J. Biol. Inorg. Chem.* **2003,** *8*, 726-732.
2. Yao, H.; Xu, Z.; Li, C.; Tse, M.-K.; Tong, Z.; Zhu, G., Synthesis and Cytotoxic Study of a Platinum (IV) Anticancer Prodrug with Selectivity toward Luteinizing Hormone-Releasing Hormone (LHRH) Receptor-Positive Cancer Cells. *Inorg. Chem.* **2019,** *58*, 11076-11084.
3. Zhang, J. Z.; Wexselblatt, E.; Hambley, T. W.; Gibson, D., Pt (IV) analogs of oxaliplatin that do not follow the expected correlation between electrochemical reduction potential and rate of reduction by ascorbate. *Chem. Commun.* **2012,** *48*, 847-849.
4. Renny, J. S.; Tomasevich, L. L.; Tallmadge, E. H.; Collum, D. B., Method of continuous variations: applications of job plots to the study of molecular associations in organometallic chemistry. *Angew. Chem. Int. Ed.* **2013,** *52*, 11998-12013.
5. Thordarson, P., Determining association constants from titration experiments in supramolecular chemistry. *Chem. Soc. Rev.* **2011,** *40*, 1305-1323.
6. Kudisch, M.; Lim, C.-H.; Thordarson, P.; Miyake, G. M., Energy Transfer to Ni-Amine Complexes in Dual Catalytic, Light-Driven C–N Cross-Coupling Reactions. *J. Am. Chem. Soc.* **2019,** *141*, 19479-19486.
7. Denis, M.; Qin, L.; Turner, P.; Jolliffe, K. A.; Goldup, S. M., A Fluorescent Ditopic Rotaxane Ion ‐ Pair Host. *Angew. Chem. Int. Ed.* **2018,** *57*, 5315-5319.
8. Frisch, M.; Trucks, G.; Schlegel, H. B.; Scuseria, G. E.; Robb, M. A.; Cheeseman, J. R.; Scalmani, G.; Barone, V.; Mennucci, B.; Petersson, G., gaussian 09, Revision d. 01, Gaussian. *Inc., Wallingford CT* **2009,** *201*.

9. Becke, A. D., Becke's three parameter hybrid method using the LYP correlation functional. *J. Chem. Phys.* **1993,** *98*, 5648-5652.
10. Lee, C.; Yang, W.; Parr, R., Density-functional exchange-energy approximation with correct asymptotic behaviour. *Phys. Rev. B* **1988,** *37*, 785-789.
11. Vosko, S. H.; Wilk, L.; Nusair, M., Accurate spin-dependent electron liquid correlation energies for local spin density calculations: a critical analysis. *Can. J. Phys.* **1980,** *58*, 1200-1211.
12. Becke, A. D., Density-functional exchange-energy approximation with correct asymptotic behavior. *Phys. Rev. A* **1988,** *38*, 3098.
13. Stephens, P. J.; Devlin, F.; Chabalowski, C.; Frisch, M. J., Ab initio calculation of vibrational absorption and circular dichroism spectra using density functional force fields. *J. Phys. Chem.* **1994,** *98*, 11623-11627.
14. Hehre, W.; Radom, L., P. v R. Schleyer and JA Pople, Ab initio Molecular Orbital Theory. Wiley, New York: 1986.
15. Dolg, M.; Wedig, U.; Stoll, H.; Preuss, H., Energy ‐ adjusted abinitio pseudopotentials for the first row transition elements. *J. Chem. Phys.* **1987,** *86*, 866-872.
16. Tomasi, J.; Mennucci, B.; Cammi, R., Quantum mechanical continuum solvation models. *Chem. Rev.* **2005,** *105*, 2999-3094.
17. Weigend, F.; Ahlrichs, R., Balanced basis sets of split valence, triple zeta valence and quadruple zeta valence quality for H to Rn: Design and assessment of accuracy. *Phys. Chem. Chem. Phys.* **2005,** *7*, 3297-3305.
18. Barr, M. P.; Gray, S. G.; Hoffmann, A. C.; Hilger, R. A.; Thomale, J.; O'Flaherty, J. D.; Fennell, D. A.; Richard, D.; O'Leary, J. J.; O'Byrne, K. J., Generation and characterisation of cisplatin-resistant non-small cell lung cancer cell lines displaying a stem-like signature. *PLoS One* **2013,** *8*, e54193.
19. Vichai, V.; Kirtikara, K., Sulforhodamine B colorimetric assay for cytotoxicity screening. *Nat. Protoc.* **2006,** *1*, 1112.
20. Santos, J. H.; Meyer, J. N.; Mandavilli, B. S.; Van Houten, B., Quantitative PCR-based measurement of nuclear and mitochondrial DNA damage and repair in mammalian cells. In *DNA Repair Protocols*, Springer: 2006; pp 183-199.
21. Fan, X.; Hussien, R.; Brooks, G. A., H2O2-induced mitochondrial fragmentation in C2C12 myocytes. *Free Radical Biol. Med.* **2010,** *49*, 1646-1654.
22. Buettner, G. R.; Jurkiewicz, B. A., Ascorbate free radical as a marker of oxidative stress: an EPR study. *Free Radical Biol. Med.* **1993,** *14*, 49-55.
23. Mah, L.; El-Osta, A.; Karagiannis, T., γH2AX: a sensitive molecular marker of DNA damage and repair. *Leukemia* **2010,** *24*, 679-686.
